# Synergistic Effect of Increased Total Protein Intake and Strength Training on Muscle Strength: A Dose-Response Meta-analysis of Randomized Controlled Trials

**DOI:** 10.1186/s40798-022-00508-w

**Published:** 2022-09-04

**Authors:** Ryoichi Tagawa, Daiki Watanabe, Kyoko Ito, Takeru Otsuyama, Kyosuke Nakayama, Chiaki Sanbongi, Motohiko Miyachi

**Affiliations:** 1 Nutrition and Food Function Research Department, Food Microbiology and Function Research Laboratories, R&D Division, Meiji Co., Ltd., 1-29-1 Nanakuni, Hachioji, Tokyo 192-0919 Japan; 2 Faculty of Sport Sciences, Waseda University, 2-579-15 Mikajima, Tokorozawa-city, Saitama 359-1192 Japan; 3 Department of Physical Activity Research, National Institute of Health and Nutrition, National Institutes of Biomedical Innovation, Health and Nutrition, 1-23-1 Toyama, Shinjuku-ku, Tokyo 162-8636 Japan

**Keywords:** Protein, Muscle strength, Resistance training, Dose-response relationship, Spline curve

## Abstract

**Background:**

Protein supplementation augments muscle strength gain during resistance training. Although some studies focus on the dose-response relationship of total protein intake to muscle mass or strength, the detailed dose-response relationship between total protein intake and muscle strength increase is yet to be clarified, especially in the absence of resistance training.

**Objective:**

We aimed to assess the detailed dose-response relationship between protein supplementation and muscle strength, with and without resistance training.

**Design:**

Systematic review with meta-analysis.

**Data Sources:**

PubMed and Ichushi-Web (last accessed on March 23, 2022).

**Eligibility Criteria:**

Randomized controlled trials investigating the effects of protein intake on muscle strength.

**Synthesis Methods:**

A random-effects model and a spline model.

**Results:**

A total of 82 articles were obtained for meta-analyses, and data from 69 articles were used to create spline curves. Muscle strength increase was significantly augmented only with resistance training (MD 2.01%, 95% CI 1.09–2.93) and was not augmented if resistance training was absent (MD 0.13%, 95% CI − 1.53 to 1.79). In the dose-response analysis using a spline model, muscle strength increase with resistance training showed a dose-dependent positive association with total protein intake, which is 0.72% (95% CI 0.40–1.04%) increase in muscle strength per 0.1 g/kg body weight [BW]/d increase in total protein intake up to 1.5 g/kg BW/d, but no further gains were observed thereafter.

**Conclusion:**

Concurrent use of resistance training is essential for protein supplementation to improve muscle strength. This study indicates that 1.5 g/kg BW/d may be the most appropriate amount of total protein intake for maintaining and augmenting muscle strength along with resistance training.

**Supplementary Information:**

The online version contains supplementary material available at 10.1186/s40798-022-00508-w.

## Key Points

- Protein supplementation improves muscle strength when combined with resistance training, irrespective of age, sex, baseline protein intake, added protein intake, or body part.
- With resistance training, muscle strength increases at the rate of 0.72% (95% CI 0.40–1.04%) per 0.1 g/kg body weight [BW]/d increase in total protein intake up to 1.5 g/kg BW/d, but no further gains are achieved thereafter.
- Muscle strength cannot be increased by protein supplementation only without resistance training.

## Introduction

All bodily movements require muscle strength [1]. Among athletes, high muscle strength affects sprint speed [2–4], agility [3, 4], jumping ability [3, 4], marathon race speed [5], aerobic endurance [3], improvement in various sport skills [4], and a reduction in injury risk [4]. Muscle strength is known to decrease with aging [6], with low muscle strength being an important factor limiting independence and autonomy among older adults [7–13]. In addition, low muscle strength increases the risk of diseases such as osteoporosis [14], low back pain [15, 16], diabetes mellitus [17], and depression [18].

Physical activity and nutrition play a crucial role in muscle strength [19, 20]. The effectiveness of resistance training in muscle strength across different age and sex groups has been confirmed in several meta-analyses [21–25]. The effect of nutrition, especially protein intake, on muscle strength has been extensively investigated in many studies [26]. Protein has a strong nutritional value for muscle because it is a major structural element of the skeletal muscle [27] and plays a key role in the promotion of muscle protein synthesis through the mammalian target of the rapamycin signaling pathway [28]. Moreover, protein intake and resistance training have also been reported to have additive and synergistic effects in the promotion of protein synthesis and muscular hypertrophy [29, 30].

Several meta-analyses showed that supplemental protein intake led to muscle strength increases in resistance training practitioners [31, 32]. However, there is no general consensus on whether supplemental protein intake would lead to muscle strength increase in people who do not perform resistance training [31–37]. Furthermore, although there are a few studies regarding the dose-response relationship of total protein intake to muscle mass [32, 38, 39] or strength [39], the detailed dose-response relationship between total protein intake and muscle strength increase is yet to be clarified, especially in the absence of resistance training. This study aimed to clarify the detailed dose-response relationship between protein supplementation and muscle strength with and without resistance training. Cross-sectional studies [40, 41] and longitudinal studies [42, 43] have shown that muscle strength is closely correlated with muscle mass. Our previous meta-analysis reported that there is a dose-response relationship between total protein intake and muscle mass increase [43]. In view of the above, we hypothesized that the effect of total protein intake on muscle strength is exerted in a dose-response manner regardless of the presence or absence of resistance training and that such an effect can be further increased through implementation of resistance training.

## Methods

### Study Design and Protocol

The study protocol of this systematic review and meta-analysis was uploaded to the UMIN Clinical Trials Registry (study ID UMIN000039285). This study aligned with the Preferred Reporting Items for Systematic Reviews and Meta-Analyses (Additional file 1: PRISMA) code [44]. We accessed the PubMed and Ichushi-Web (online academic article repository in Japan) databases up to March 23, 2022, for randomized controlled trials (RCTs) investigating the effects of protein intake on muscle strength and published in English or Japanese. The search phrases and parameters are listed in Additional file 2: Table S1. Manual searches were also conducted on studies included in other meta-analyses.

### Study Identification and Data Extraction

The titles and abstracts of all the articles retrieved were independently screened by two authors (R.T. and K.I.). Eligibility was evaluated using the criteria illustrated in the following section, and any disagreements between the two authors were resolved through discussions. Articles deemed eligible and those warranting no decision during primary screening underwent secondary screening to make a final decision on eligibility according to their full text. Information on characteristics of participants, intervention conditions, and the target results were retrieved from the eligible articles during secondary screening.

For trials consisting of more than one intervention group, each group was considered a separate trial. For trials measuring the muscle strength of more than one body part or contraction mode of the same participant, the separate data were combined to one outcome with weighted mean value. Measurements taken midway through the intervention period were omitted, and only one pre- and post-intervention result was analyzed. The corresponding authors of the eligible articles were contacted when information needed to create a forest plot was not available. When numerical data were unavailable from the corresponding author, but the data were available as graphs, numerical values were retrieved using the web-based tool WebPlotDigitizer, version 4.1 (Ankit Rohatgi; Pacifica, CA) [45]. Secondary screening and information retrieval were carried out by five authors (K.I., T.O., R.T., C.S., and K.N.), while the studies were verified by two authors (K.I. and T.O.). We used Microsoft Excel, Version 2016 (Microsoft Corporation, Redmond, WA) and EndNote, version 9 (Clarivate, Philadelphia, PA) for screening and data extraction.

### Eligibility

RCTs on the effects of protein intake on muscle strength, where supplemented protein intakes differed between study groups, were included in the analysis. The population, intervention, comparison, outcome, and study design criteria were employed to determine study eligibility (Table 1). The sample population was narrowed down to study participants without any serious injury or illness (e.g., human immunodeficiency virus infection, cancer, chronic renal failure, terminal illness, or diseases that adversely impact physical activity). The protein intervention timeframe was set at ≥ 2 weeks, based on a previous study that found this period was an adequate length of time for protein intake to enhance lean body mass [46] and so that crucial research information from all potentially eligible RCTs could be collected. Supplemental protein intake (g/d or g/kg body weight [BW]/d) was the value determined before the intervention. Trials with muscular hypertrophic agents (e.g., leucine, β-hydroxy-β-methyl butyrate, creatine or vitamin D), energy restriction or overfeeding were excluded, after consulting previous meta-analyses [31, 32, 34, 35, 39]. For trials with two or more control groups, the group with the same energy intake and larger gap in the supplemented protein intake was included as a control in the analysis. Trials with between-group comparisons for different conditions other than nutrition (such as exercise) were excluded.

**Table 1:** PICOS criteria for study inclusion

Parameter	Inclusion criterion
Population	Adult participants (not injured or critically ill)
Intervention	Supplementary protein intake for ≥ 2 weeks
Comparator	Placebo or no intervention
Outcome	Muscle strength
Study design	Randomized controlled trial

### Outcome Measures

The present meta-analysis assessed muscle strength. For muscle strength, two values were recorded: intragroup percentage change in muscle strength and the intergroup difference in muscle strength percentage changes between an intervention group and a control group. The former was used to evaluate the intragroup effect of total protein intake (the sum of supplemented protein intake and dietary protein intake). The latter was used to evaluate the intergroup effect of supplemented protein intake excluding the effect of conditions other than nutrition.

### Quality Assessment

Two authors (K.I. and T.O.) independently reviewed the quality of the eligible articles using the Cochrane risk-of-bias tool [47]. Discrepancies were resolved through a discussion with a third author (R.T.). We excluded articles with high risk of bias in at least two out of seven domains. Publication bias was assessed visually using a funnel plot. We categorized the quality of evidence for analyses of all studies, studies without resistance training or studies with resistance training as high, moderate, low, or very low according to the Grading of Recommendations Assessment Development and Evaluation (GRADE) system [48].

### Statistical Analysis

This meta-analysis evaluated the mean change in muscle strength and the standard deviation (SD) of change (SD_change_). In studies where SD_change_ was not described, but SD_baseline_ and SD_final_ were known, SD_change_ was computed using the following formula [49]:

$${\text{SD}}_{{{\text{change}}}} = \, \surd ( {{\text{SD}}_{{{\text{baseline}}}}^{{2}} + {\text{ SD}}_{{{\text{final}}}}^{{2}} - { 2 } \times {\text{ Corr }} \times {\text{ SD}}_{{{\text{baseline}}}} \times {\text{ SD}}_{{{\text{final}}}} } )$$

In studies where all information for SD before the intervention (SD_baseline_), SD after the intervention (_final_), and SD_change_ were available, the correlation coefficient (Corr) was computed using the following equation:

$${\text{Corr }} = \, ( {{\text{SD}}_{{{\text{baseline}}}}^{{2}} + {\text{ SD}}_{{{\text{final}}}}^{{2}} - {\text{ SD}}_{{{\text{change}}}}^{{2}} } )/( {{2 } \times {\text{ SD}}_{{{\text{baseline}}}} \times {\text{ SD}}_{{{\text{final}}}} } )$$

In cases where the information necessary for analysis was unavailable, we requested it from the corresponding author of the article (11 responses from 22 requests were obtained). We used Corr = 0.96 for handgrip strength, Corr = 0.89 for arm muscle strength, Corr = 0.85 for leg muscle strength, Corr = 0.97 for breast muscle strength, Corr = 0.89 for back muscle strength, Corr = 0.96 for total muscle strength (used when only the sum of several muscle strengths was available). The studies used for calculations are listed in Additional file 2: Table S2.

The effect of supplemental protein intake on muscle strength percentage change, with the control group as reference, was analyzed using point estimation and projected as a forest plot of the point estimates of the MD and 95% CI. All analyses were stratified by the presence or absence of resistance training. To evaluate the influence of age (< 60 or ≥ 60 years), sex (male or female), baseline protein intake (< 1.0 or ≥ 1.0 g/kg BW/d), added protein intake (< 0.5 or ≥ 0.5 g/kg BW/d), intervention duration (< 3 or ≥ 3 months), contraction type (isokinetic, isometric, or isotonic), and body part (upper or lower body), subgroup analyses were also performed. All the above analyses were performed using a random-effects model under the assumption that trial errors were included because the selected trials employed various conditions and were not confined to specific sex, age, or exercise conditions. Statistical heterogeneity was evaluated using inconsistency index (*I*^2^) [50] and *χ*^2^ test as a guide to estimate dispersion for all analyses.

In addition, the spline model was employed in the analysis of the dose-response relationship between total protein intake and muscle strength percentage change from baseline. Stratification according to the existence or absence of resistance training was also performed, and the results were presented as the effect size and 95% CI. The mean effect size, along with the corresponding 95% CI for a percentage gain in muscle strength, was estimated for every 0.1 g/kg BW/d increase in total protein intake above 1.5 g/kg BW/d using the spline models. We set the value to 1.5 g/kg BW/d in the analysis with resistance training and 1.3 /kg BW/d without resistance training as this is the inflexion point based on which total protein intake is correlated with muscle strength. During these evaluations, when the 95% CI of the magnitude of effect did not reach 0, the *P* value was estimated as < 0.05. In contrast, when the 95% CI of the extent of the resulting outcome reached 0, the *P* value was estimated as ≥ 0.05.

All statistical analyses were performed using Review Manager (RevMan), version 5.4 (NordicCochrane Centre; Cochrane Collaboration, Copenhagen, Denmark) and Stata/M.P., version 15.0 (StataCorp L.P.; College Station, TX). A two-sided *P* value of < 0.05 was considered statistically significant.

## Results

### Study Selection

**Fig. 1:**
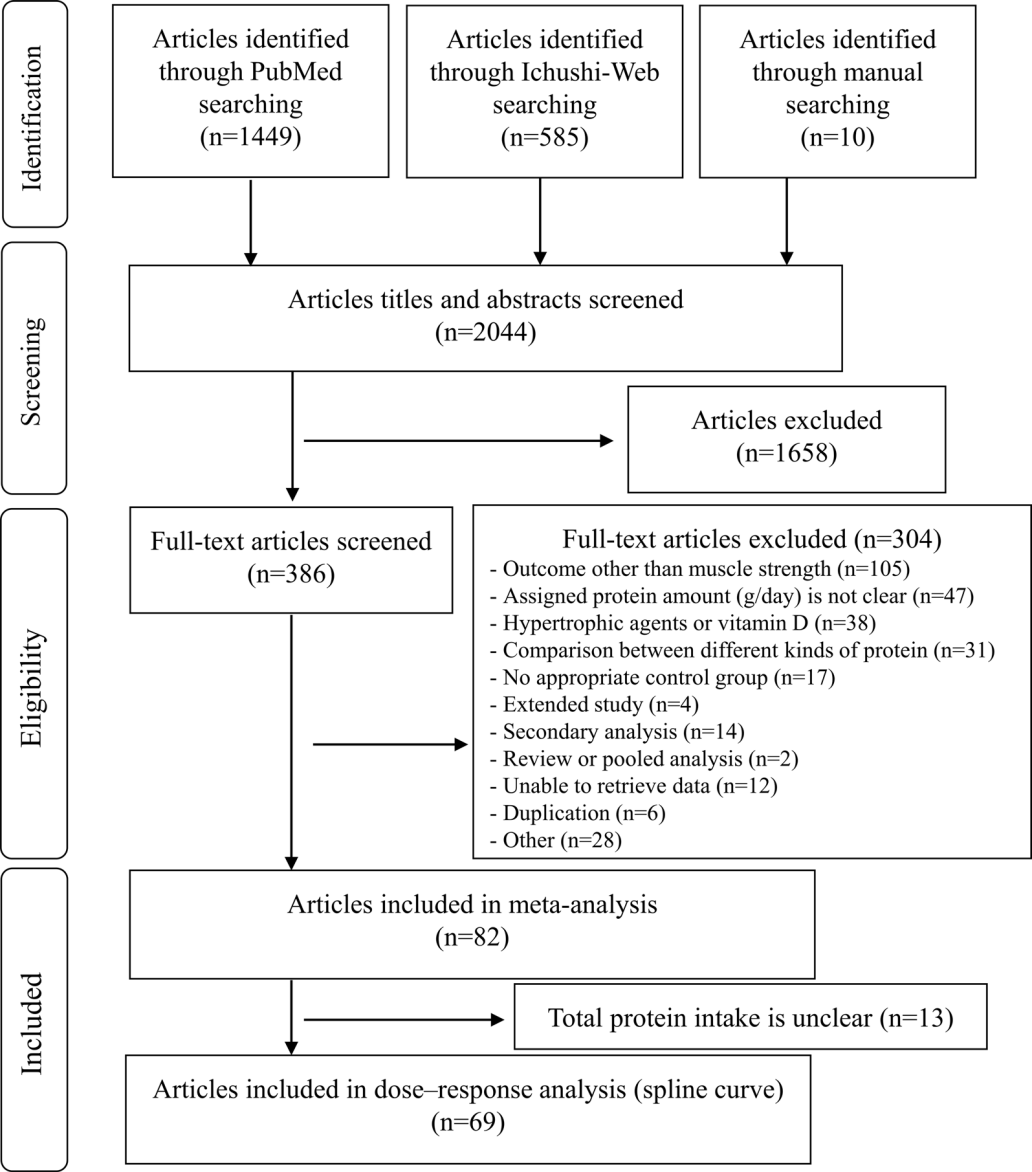
Flow diagram of the literature search process

The results of the literature search are presented in Fig. 1. Of the 2044 articles initially screened, 386 were reviewed after primary screening of their titles and abstracts. Among them, 82 articles involving 112 intervention groups and 3940 participants were included after a secondary screening of their full text. In total, 59 studies involving 82 intervention groups and 2440 participants described resistance training, while, 24 studies involving 30 intervention groups and 1500 participants did not include resistance training. One study included trials both with and without resistance training. The 82 articles were subsequently utilized to create a forest plot to assess the effects of supplemental protein intake on muscle strength percentage changes with reference to the control groups. We used 69 articles to create spline models to examine the correlation between total protein intake and muscle strength percentage changes from baseline.

### Study Characteristics

Table 2 gives a summary of the characteristics of the included studies. Total protein intake ranged from 0.79 to 3.80 g/kg BW/d (mean, 1.49 g/kg BW/d) in the intervention groups and from 0.69 to 2.00 g/kg BW/d (mean, 1.09 g/kg BW/d) in the control groups. The differences in supplemental protein intake between the intervention and control groups ranged from 0.04 to 2.06 g/kg BW/d (mean, 0.38 g/kg BW/d). There were 78 RCTs in which protein-rich test food/beverage was supplemented along with the usual meals and 4 RCTs in which the content of the meal itself was altered. The intervention period ranged from 3 weeks to 2 years, with an average of 22.0 weeks. A total of 1859 participants were female, while 2073 participants were male. The sex of the remaining 8 participants was unknown (data not available). The participants were aged between 19 and 87 years, with an average of 55.6 years. The participant and intervention characteristics are shown in Additional file 3: Tables S3–S6.

**Table 2:** Summary of the characteristics of the included studies

	RT	Non-RT	All
Number of studies	59	24	82
Number of participants	2440	1500	3940
Age (years)	45.5	72.1	55.6
Number of participants aged 19–59	1461	81	1542
Number of participants aged 60–87	923	1389	2312
Sex (men %)	62	38	53
Race (Caucasian %)	88	63	78
Height (cm)	171	164	168
Number of participants (149–169 cm)	768	1022	1790
Number of participants (170–185 cm)	1460	329	1789
Body weight (kg)	78.8	68.1	74.8
Number of participants (46–74 kg)	672	977	1649
Number of participants (75–102 kg)	1631	395	2026
Body mass index (kg/m^2^)	26.6	25.4	26.1
Number of participants (20.6–24.9 kg/m^2^)	768	516	1284
Number of participants (25.0–37.9 kg/m^2^)	1597	963	2560
Energy intake (kcal/day)	2141	1752	2035
Number of participants (1298–1999 kcal/day)	561	418	979
Number of participants (2000–3539 kcal/day)	819	100	919
Baseline protein intake (g/kg BW/d)	0.39	0.37	0.38
Number of participants (0.60–0.99 g/kg BW/d)	561	314	875
Number of participants (1.00–2.10 g/kg BW/d)	1235	962	2197
Added protein intake (g/kg BW/d) (intervention groups)	0.39	0.37	0.38
Number of participants (0.04–0.49 g/kgBW/d)	1036	582	1618
Number of participants (0.50–2.06 g/kgBW/d)	274	212	486
Total protein intake (g/kg BW/d) (intervention groups)	1.50	1.46	1.49
Number of participants (0.79–1.49 g/kgBW/d)	708	393	1101
Number of participants (1.50–3.80 g/kgBW/d)	472	311	783
Trial periods (weeks)	15.1	33.3	22.0
Number of participants (3 weeks to 2.9 months)	1845	719	2564
Number of participants (3 months to 2 years)	595	781	1376
Habitual training (%)	13	0	8
Body part			
Upper body (%)	64	83	72
Lower body (%)	78	57	70
Contraction type			
Isokinetic (%)	15	14	15
Isometric (%)	37	96	59
Isotonic (%)	82	13	55

Data are shown as the mean values weighted by the number of participants

*RT* resistance training, *BW* body weight

### Risk of Bias

There were four articles (4.7%) with high risk of bias in at least two domains and they were excluded from our analyses. Specific data are shown in Additional file 4: Fig. S1. Publication bias was not detected in the analysis of all studies, but detected in analyses of studies without resistance training, above 60 years old or < 1.0 g/kg BW/d baseline protein intake, based on visual inspection of funnel plots. Funnel plots for each sub-analysis are shown in Additional file 4: Fig. S2.

### Meta-analysis

The meta-analysis of the trials for 112 intervention conditions revealed a significant effect of protein intake on an increase in muscle strength percentage, with a mean difference (MD) of 1.40% (95% CI 0.55–2.24; Table 3, Additional file 4: Fig. S3) in muscle strength increase between the intervention and control groups. When stratified according to the presence or absence of resistance training, muscle strength percentage increase was significantly increased only with resistance training (MD: 2.01%, 95% CI 1.09–2.93) and not without resistance training (MD: 0.13%, 95% CI − 1.53 to 1.79), irrespective of age, sex, baseline protein intake, added protein intake, or body part. Meanwhile, a subgroup analysis according to intervention duration showed that muscle strength did not significantly increase with protein intake in long trials (≥ 3 months), irrespective of resistance training. Regarding contraction type, only isotonic strength was significantly augmented by protein intake in the overall trials or trials with resistance training. There were dose-response effects of added protein intake on muscle strength with resistance training. Although muscle strength with resistance training was significantly augmented even when the added protein intake was below 0.5 g/kg BW/d (MD: 1.22%, 95%CI: 0.41 to 2.03), a significantly larger increase in muscle strength was achieved when added protein intake was above 0.5 g/kg BW/d (MD: 4.29%, 95%CI 1.99–6.60), which is 3.5-fold the less protein intake. For statistical heterogeneity, the I^2^ statistic was 32%, 16%, and 49% for the overall trials, trials with resistance training, and trials without resistance training, respectively, and the *χ*^2^ tests demonstrated significant heterogeneity for overall trials and trials without resistance training (*P* < 0.01).

**Table 3:** Subgroup analyses of the effects of protein intake on muscle strength

	RT			Non-RT			All		
	*N*/study	MD (95% CI) (%)	*I*^2^ (%)	*N*/study	MD (95% CI) (%)	*I*^2^ (%)	*N*/study	MD (95% CI) (%)	*I*^2^ (%)
All	2440/59	2.01 (1.09, 2.93)	16	1500/24	0.13 (− 1.53, 1.79)	49	3940/82	1.40 (0.55, 2.24)	32
Subgroup analysis									
Age (years)									
< 60	1461/37	2.35 (1.00, 3.69)	27	81/2	0.95 (− 4.97, 6.86)	46	1542/39	2.25 (0.95, 3.54)	27
≥ 60	923/22	1.38 (0.39, 2.36)	0	1389/21	0.20 (− 1.65, 2.04)	53	2312/42	0.64 (− 0.47, 1.75)	35
Sex									
Female	403/13	1.54 (0.46, 2.62)	0	292/5	2.48 (− 5.43, 10.39)	74	695/18	1.38 (− 0.28, 3.04)	28
Male	1078/33	2.07 (0.71, 3.43)	9	105/3	− 0.95 (− 3.51, 1.60)	0	1183/35	1.64 (0.37, 2.91)	13
Baseline protein intake (g/kg BW/d)									
< 1.0	561/12	1.46 (0.45, 2.48)	0	314/6	− 0.26 (− 2.56, 2.03)	20	875/18	1.06 (− 0.01, 2.13)	11
≥ 1.0	1235/31	2.11 (0.76, 3.46)	0	962/13	− 0.81 (− 3.00, 1.38)	51	2197/43	0.93 (− 0.35, 2.21)	32
Added protein intake (g/kg BW/d)^b^									
< 0.5	1849/45	1.22 (0.41, 2.03)	0	1056/18	1.38 (− 0.95, 3.71)	57	2905/63	1.10 (0.17, 2.03)	22
≥ 0.5	538/14	4.29 (1.99, 6.60)	40	356/6	− 1.64 (− 3.52, 0.25)	0	894/19	2.36 (0.35, 4.37)	55
Trial periods (months)^a^									
< 3	1845/50	2.27 (1.23, 3.30)	19	719/15	0.95 (− 1.56, 3.46)	53	2564/64	1.90 (0.91, 2.90)	31
≥ 3	595/9	0.66 (− 1.22, 2.54)	0	781/9	− 0.67 (− 2.84, 1.50)	44	1376/18	− 0.05 (− 1.52, 1.43)	26
Contraction type									
Isokinetic	370/7	0.71 (− 4.69, 6.11)	59	217/3	− 1.11 (− 5.25, 3.03)	45	587/10	− 0.03 (− 3.53, 3.47)	54
Isometric	892/18	0.92 (− 0.33, 2.17)	7	1439/22	0.26 (− 1.70, 2.23)	60	2331/39	0.33 (− 0.91, 1.56)	47
Isotonic	1989/49	2.30 (1.25, 3.36)	21	191/6	0.57 (− 1.99, 3.13)	0	2180/54	2.13 (1.16, 3.11)	19
Body part									
Upper body	1570/44	1.39 (0.25, 2.54)	52	1252/20	0.15 (− 1.65, 1.95)	59	2822/63	0.99 (0.01, 1.98)	56
Lower body	1898/50	2.77 (1.46, 4.07)	39	853/15	0.40 (− 1.82, 2.62)	24	2751/64	2.21 (1.07, 3.34)	38

*RT* resistance training, *MD* mean difference, *BW* body weight

^a^Statistically significant difference subgroups in the analysis of all trials (*P* < 0.05)

^b^Statistically significant difference subgroups in the analysis of trials with RT (*P* < 0.05)

### Dose-Response Analyses with Spline Models

**Fig. 2:**
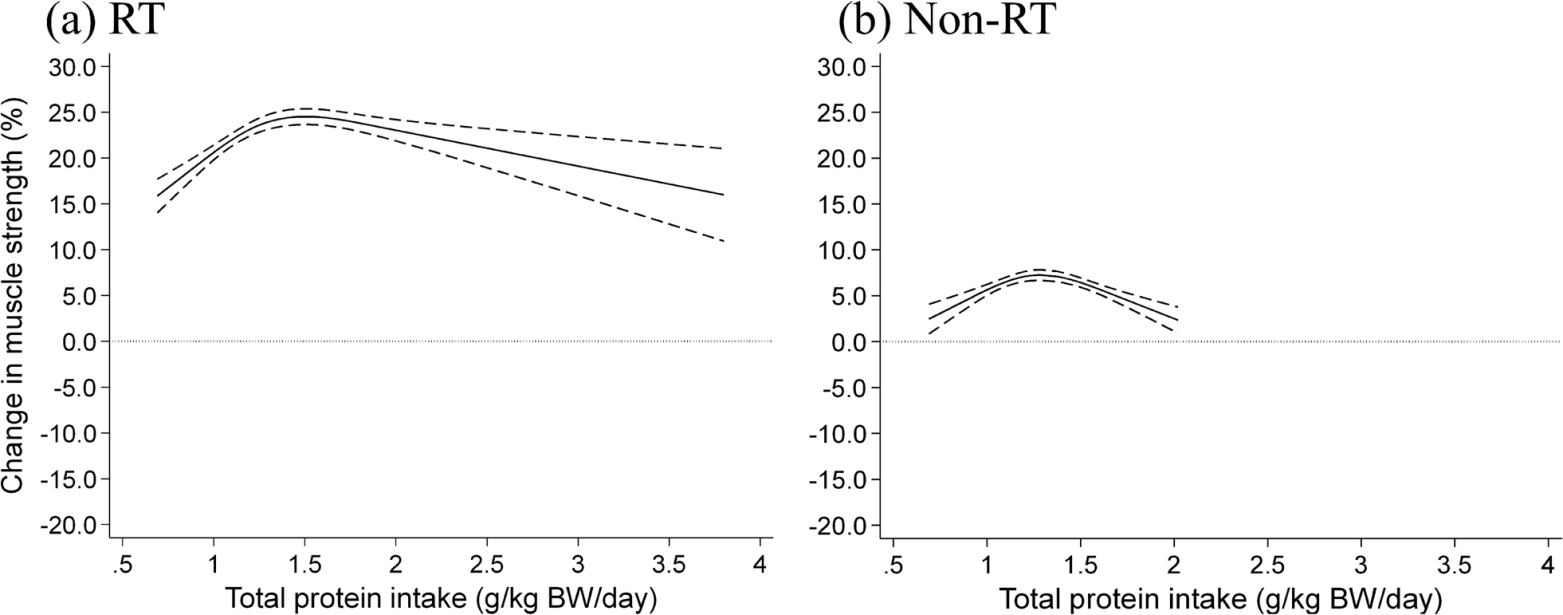
Dose-response relationship between total protein intake and change in muscle strength. Spline curves illustrating the associations between total protein intake and change% in muscle strength for **a** trials with RT, and **b** trials without RT. The solid line and dashed line represent the mean change% in muscle strength and 95% CI, respectively. Abbreviations: RT, resistance training; BW, body weight

The results of the dose-response analyses using spline curves are presented in Fig. 2. The percentage change in muscle strength gradually increased with total protein intake and peaked at approximately 1.5 g/kg BW/d with resistance training (Fig. 2a). Muscle strength with resistance training increased by 0.72% (95% CI 0.40–1.04%) per 0.1 g/kg BW/d increase in protein intake up to 1.5 g/kg BW/d, but no further gains were observed thereafter. Without resistance training, there was only a fractional increase in muscle strength, which increased slightly with total protein intake up to 1.3 g/kg BW/d and gradually vanished after that (Fig. 2b).

### GRADE

Table 4 shows the GRADE assessment for our analyses. The overall quality of the evidence was graded as high for the analysis of all studies and the analysis of studies with resistance training, and graded as moderate for the analysis of studies without resistance training due to possible publication bias.

**Table 4:** Summary of the GRADE assessment

	Study design	Risk of bias	Inconsistency	Indirectness	Imprecision	Publication bias	Absolute effect (95%CI) (%)	Quality
With RT	RCT	Not serious	Not serious	Not serious	Not serious	Not serious	2.01 (1.09, 2.93)	⨁⨁⨁⨁ High
Without RT	RCT	Not serious	Not serious	Not serious	Not serious	Serious	0.13 (− 1.53, 1.79)	⨁⨁⨁◯ Moderate
All	RCT	Not serious	Not serious	Not serious	Not serious	Not serious	1.40 (0.55, 2.24)	⨁⨁⨁⨁ High

*RT* resistance training

## Discussion

### Major Findings

This study found the following three major findings. First, protein intake leads to a significant improvement in muscle strength only when combined with resistance training. Second, the spline model results showed that 1.5 g/kg BW/d total protein intake with resistance training is required to achieve the optimal effect on muscle strength. To the best of our knowledge, while a few studies have investigated the dose-response relationship of total protein intake to muscle mass [32, 38, 39] or strength [39], this study is the first meta-analysis to quantitatively assess the detailed dose-response relationship between total protein intake and muscle strength in both training and non-training study participants.

### Clinical Significance of the Study

In addition to its correlation with good physical abilities such as high balance skills, low fall risk [51, 52], and excellent athletic performance [2, 53], high muscle strength is also known as an important indicator of physical fitness associated with high bone density [54]; lung function [55]; and low risk of depressive disorder [56], diabetes mellitus [17], and death [57]. Notably, physical activity guidelines actively encourage muscular strengthening activity to promote good health [58–63]. Thus, long-term maintenance/improvement of muscle strength is recommended not only for individuals with active lifestyles, such as athletes, but also for those with normal physical activity.

Several meta-analyses have confirmed the effectiveness of resistance training in increasing muscle strength across age and sex groups [21–25]. There have also been several meta-analyses on the effect of protein intake on muscle strength [31–37]. However, the results have been conflicting as to the association between protein intake and muscle strength according to factors such as age [31, 33–36] and presence or absence of resistance training [31–33].

In the present study, we used a stratification model to determine the effects of protein supplementation on muscle strength. For people who perform resistance training for several months, a further mean increase of 2% in muscle strength with protein supplementation, as revealed in our analysis, is clinically important given the fact that muscle strength decrease with aging is less than 1%/year for young adults [64] and less than 3%/year for older people [64, 65]. The dose-response relationship was also evaluated using a spline model, and the results showed that the effect of protein supplementation varies greatly according to the presence or absence of resistance training. These findings provide a new understanding of the effects and limitations of protein intake on muscle strength.

### Importance of Resistance Training for Muscle Strength

The current findings confirm that protein supplementation significantly increases muscle strength only when the supplementation is combined with resistance training. The above findings are in contrast with our initial hypothesis and show that supplemental protein intake alone has no effect on muscle strength and needs to be combined with resistance training. These results are consistent with those of a large-scale RCT that examined the individual impact of resistance training and protein intake on muscle strength [66].

Muscle strength augmentation is the result of changes in the muscle structure such as muscular hypertrophy [67, 68], neural adaptations such as increased mobilization of motor units [67, 69, 70], and metabolic adaptations of anaerobic energy [71–73]. Resistance training was found to initiate all these three mechanisms [67–73]. Meanwhile, supplemental protein intake was found to affect only muscular hypertrophy [29, 30, 74, 75] or play a minor role in both neural and metabolic adaptations [76, 77]. Although the detailed mechanisms have not yet been elucidated, it is thought that protein intake by itself is not sufficient to induce neural and metabolic adaptations. This may be one of the reasons that protein intake alone could not effectively improve muscle strength. The idea that muscle mass alone cannot account for all the changes in muscle strength is supported by previous reviews and meta-analyses, which reported that resistance training has a more profound effect on muscle strength than on muscle mass [24, 25, 78]. Further, a cross-sectional study showed that muscle mass accounts for only 11–40% of muscle strength in older adults [79] and longitudinal studies have shown that muscle strength declined faster than did muscle mass in older adults [40, 80].

### Types of Muscle Strength Measurement/Assessment and Effects of Protein Intake

The results of muscle strength measurement based on stratified analysis established the synergistic effect of resistance training and protein intake on isotonic strength. However, protein intervention had no effect on isometric and isokinetic strength. This is thought to be due to the following two reasons. First, although a supplemental dose of protein is known to affect overall muscle mass in the whole body, the lack of effectiveness of protein intervention on isometric and isokinetic strength might be due to the higher degree of muscle mass mobilization required under isotonic movement during multi-joint tasks (e.g., squat, deadlift, bench press exercises). Meanwhile, a lower degree of muscle mass mobilization is required in isometric strength (e.g., grip strength) and isometric movement during single-joint tasks. Second, because resistance training consists almost exclusively of isotonic exercises requiring the use of training equipment such as free weights and weight machines, it is likely that effects on muscle strength are visible only in isotonic strength measurements.

### Dose-Response Relationship Between Total Protein Intake and Muscle Strength

The spline model results showed that muscle strength with resistance training increased proportionally at the rate of 0.72% (95% CI 0.40–1.04%) per 0.1 g/kg BW/d increase in total protein intake up to 1.5 g/kg BW/d, but no further gains are achieved thereafter. This result resembles that of a previous meta-analysis [39] to some degree, in which the effects of total protein intake on bench press strength were not different between below and above 1.6 g/kg BW/d. However, further researches are needed to better understand the mechanism regulating the effectiveness of total protein intake at 1.5 g/kg BW/d for muscle strength.

### Generalizability of Results

Our literature review identified two meta-analyses [31, 32] that investigated the relationship between protein intake and muscle strength, and calculated the effect size as the MD. The effect sizes of protein intake on muscle strength in combination with resistance training were 2.01% (95% CI 1.09–2.93) for all measurements and 4.43% (95% CI 2.27–6.58) for leg press in the present study. These are smaller than those of previous meta-analysis studies, which were 4.12% (95% CI: 1.06 to 7.16) for all measurements [32] and 7.74% (95% CI 3.67–11.87) for leg press [31]. Our meta-analysis includes numerous articles, which leads to differences in study characteristics from the previous meta-analyses. These discrepancies of effect sizes may be partially attributable to the added protein intake, which is around 70–85% of those of previous studies or to the age of participants in the present study, which is more than 10 years higher than that of subjects of the previous studies (on average), because our analyses showed that the effect of protein intake on muscle strength in combination with resistance training is significantly higher with more added protein intake than less added protein intake, and the mean value was 1.7 times larger for younger than for older adults. Moreover, the dose-response relationship between total protein intake and muscle strength augmentation in the present study resembles the result of the previous meta-analysis in that gain in muscle mass with increasing total protein intake begins to plateau at similar total protein intake (1.5 or 1.6 g/kg BW/d) [32]. There were no major differences between our findings and the results of previous meta-analyses.

### Adverse Effects of Excess Protein Intake

Although the beneficial role of protein intake in maintaining and enhancing muscle strength is well documented, the potential adverse effects of excess protein intake also need to be considered. High protein intake during pregnancy has been reported to increase the risk of small-for-gestational-age births [81] and neonatal death [82]. Conflicting results were reported regarding adverse effects of protein intake on renal function [83, 84]. Particularly, high protein intake from animal sources other than milk is associated with lower renal function in individuals with mild renal impairment [85]. Thus, it is important, especially for the above at-risk population, to consume a moderate amount of protein to maintain nitrogen balance and avoid the risks associated with excessive protein intake.

### Strengths and Limitations of the Study

The present study has several strengths. First, unlike many meta-analyses that evaluated the effect of protein intake on muscle strength increase only in combination with resistance training, our study highlights the differences in the effect of protein intake between presence and absence of resistance training. Second, the present study provides evidence to further understand and determine the detailed quantitative relationship between total protein intake and muscle strength increase using the spline model, a topic that had not been adequately explored in the past. However, this study also has several limitations. First, the studies included in this study were limited to those published in English and Japanese. Second, studies were only sourced from PubMed and Ichushi-Web database. As such, other relevant studies may have been missed out. However, we believe that this had limited impact because of the larger number of papers included in the current study compared to previous studies. Third, the data were highly heterogeneous as the studies were diverse. As such, differences in specific conditions should be considered when interpreting the results of this study. Finally, although using our spline model provides evidence to further understand and determine the detailed quantitative relationship between total protein intake and muscle strength increase, it is a data-oriented approach and does not consider the physiology of protein and its utilization. Thus, the physiological mechanism behind the quantitative relationship should be further studied for complete understanding.

## Conclusion

Concurrent use of resistance training is essential for protein supplementation to improve muscle strength. The effect becomes higher with more total protein intake up to 1.5 g/kg BW/d, but no further gains are achieved thereafter.

## Supplementary Information

**Additional file 1. **PRISMA 2020 Checklist.

**Additional file 2.** Search strategy for PubMed and Ichushi-Web, Studies used to calculate the correlation coefficient for SD_change_.

**Additional file 3**. Summary of characteristics of included studies, Summary of nutrition surveys, Summary of assigned protein amounts and differences between groups, Summary of conditions of the studies’ interventions.

**Additional file 4. **Risk-of-bias assessment, Funnel plots of studies with or without resistance training for changes in muscle strength, Forest plot assessing the effect of protein supplementation on changes in muscle strength.

## Data Availability

The datasets generated and analyzed during the current systematic review and meta-analysis are available from the corresponding author on reasonable request.
