# Supplementary material for: Synergistic Effect of Increased Total Protein Intake and Strength Training on Muscle Strength: A Dose-Response Meta-analysis of Randomized Controlled Trials
Source: Sports Med Open. 2022 Sep 4;8:110. doi: 10.1186/s40798-022-00508-w (PMC9441410; doi:10.1186/s40798-022-00508-w)
Supplement: Supplementary file 2 — Additional file 2. Search strategy for PubMed and Ichushi-Web, Studies used to calculate the correlation coefficient for SDchange. [file 40798_2022_508_MOESM2_ESM.docx]

**Additional file 2**

**Synergistic effect of increased total protein intake and strength training on muscle strength:**

**A dose–response meta-analysis from randomized controlled trials**

Ryoichi Tagawa^1 #^

Daiki Watanabe^2, 3 #^

Kyoko Ito^1^

Takeru Otsuyama^1^

Kyosuke Nakayama^1^

Chiaki Sanbongi^1^

Motohiko Miyachi^2, 3 *^

^1^ Nutrition and Food Function Research Department, Food Microbiology and Function Research Laboratories, R&D Division, Meiji Co., Ltd., 1-29-1 Nanakuni, Hachioji, Tokyo 192-0919, Japan

^2^ Faculty of Sport Sciences, Waseda University, 2-579-15 Mikajima, Tokorozawa-city, Saitama 359-1192, Japan

^3^ Department of Physical Activity Research, National Institute of Health and Nutrition, National Institutes of Biomedical Innovation, Health and Nutrition, 1-23-1 Toyama, Shinjuku-ku, Tokyo 162-8636, Japan

# RT and DW equally contributed to the manuscript and are joint first authors.

* Corresponding Author: Motohiko Miyachi

Email: cardiovascular0327@mac.com

| *Supplementary Table S1* **Search strategy for PubMed and Ichushi-Web** | |
| --- | --- |
| Last search performed: March 23rd, 2022. Total number of citations: 2034 | |
|  |  |
| **Database** | **Search criteria** |
| **PubMed** | (protein*[Title] OR whey*[Title] OR casein*[Title] OR glycinin*[Title] OR conglycinin*[Title] OR gliadin*[Title] OR glutenin*[Title] OR gluten*[Title] OR albumin*[Title] OR ovalbumin*[Title]) |
|  | AND (random*) |
|  | AND (man or men or woman or women or subject or subjects or participant or participants or volunteer or volunteers or recruit or recruits) |
|  | AND (muscle or strength or fat) |
|  | NOT (review[Title] OR meta-analysis[Title] OR meta-regression[Title]) |
| **Ichushi-Web** | protein (original words were in Japanese) |
|  | AND (muscle or fat) (original words were in Japanese) |
|  | **Limit:** |
|  | Language: English or Japanese |
|  | Article types: Original article AND Randomized controlled trial AND Clinical trial |

*Supplementary Table S2* **Studies used to calculate the correlation coefficient for SD_change_**

| **Muscle strength** | **Studies** |
| --- | --- |
| handgrip strength | Alemán-Mateo (2014) [1], Mori (2014) [2], Thomson (2016) [3], Niccoli (2017) [4], Bartholomae (2019) [5], Drotningsvik (2019) [6], ten Haaf (2019) [7], Krull (2020) [8], Li (2021) [9], Ullevig (2021) [10] |
| arm muscle strength | Erskine (2012) [11], Nabuco (2018) [12], Krull (2020) [8] |
| leg muscle strength | Hoffman (2007) [13], Hulmi (2009) [14], Verdijk (2009) [15], Farup (2014) [16], Mori (2014) [2], Hulmi (2015) [17], Reidy (2016) [18], Taylor (2016) [19], Thomson (2016) [3], Niccoli (2017) [4], Rossato (2017) [20], Nabuco (2018) [12], Orsatti (2018) [21], Bartholomae (2019) [5], Jonvik (2019) [22], ten Haaf (2019) [7] , Krull (2020) [8] |
| breast muscle strength | Hoffman (2007) [13], Hulmi (2009) [14], Weisgarber (2012) [23], Taylor (2016) [19], Nabuco (2018) [12], Rossato (2017) [20], Orsatti (2018) [21], Sharp (2018) [24], Krull (2020) [8] |
| back muscle strength | Orsatti (2018) [21], Sharp (2018) [24], Krull (2020) [8] |
| total muscle strength | Iglay (2007) [25], Snijders (2015) [26], Gaffney (2018) [27], Nabuco (2018) [12] |

References

1. Alemán-Mateo H, Carreón VR, Macías L, et al. Nutrient-rich dairy proteins improve appendicular skeletal muscle mass and physical performance, and attenuate the loss of muscle strength in older men and women subjects: a single-blind randomized clinical trial. Clin Interv Aging 2014; 9: 1517-1525

2. Mori H, Niwa M. Effect of nutritional care and whey protein supplementation on the body composition and physical function in older adults after combined resistance and aerobic exercise. Jpn J Nutr Diet 2014; 72: 12-20

3. Thomson RL, Brinkworth GD, Noakes M, et al. Muscle strength gains during resistance exercise training are attenuated with soy compared with dairy or usual protein intake in older adults: a randomized controlled trial. Clin Nutr 2016; 35: 27-33

4. Niccoli S, Kolobov A, Bon T, et al. Whey protein supplementation improves rehabilitation outcomes in hospitalized geriatric patients: a double blinded, randomized controlled trial. J Nutr Gerontol Geriatr 2017; 36: 149-165

5. Bartholomae E, Incollingo A, Vizcaino M, et al. Mung bean protein supplement improves muscular strength in healthy, underactive vegetarian adults. Nutrients 2019; 11: 2423

6. Drotningsvik A, Oterhals Å, Flesland O, et al. Fish protein supplementation in older nursing home residents: a randomised, double-blind, pilot study. Pilot Feasibility Stud 2019; 5: 35

7. ten Haaf DSM, Eijsvogels TMH, Bongers C, et al. Protein supplementation improves lean body mass in physically active older adults: a randomized placebo-controlled trial. J Cachexia Sarcopenia Muscle 2019; 10: 298-310

8. Krull MR, Howell CR, Partin RE, et al. Protein Supplementation and Resistance Training in Childhood Cancer Survivors. Med Sci Sports Exerc 2020; 52: 2069-2077

9. Li C, Meng H, Wu S, Fang A, Liao G, Tan X, et al. Daily Supplementation With Whey, Soy, or Whey-Soy Blended Protein for 6 Months Maintained Lean Muscle Mass and Physical Performance in Older Adults With Low Lean Mass. Journal of the Academy of Nutrition and Dietetics. 2021 Jun;121(6):1035-48.e6.

10. Ullevig SL, Zuniga K, Austin Lobitz C, Santoyo A, Yin Z. Egg protein supplementation improved upper body muscle strength and protein intake in community-dwelling older adult females who attended congregate meal sites or adult learning centers: A pilot randomized controlled trial. Nutrition and health. 2021 Nov 3:2601060211051592.

11. Erskine RM, Fletcher G, Hanson B, et al. Whey protein does not enhance the adaptations to elbow flexor resistance training. Med Sci Sports Exerc 2012; 44: 1791-1800

12. Nabuco HCG, Tomeleri CM, Fernandes RR, et al. Effect of whey protein supplementation combined with resistance training on body composition, muscular strength, functional capacity, and plasma-metabolism biomarkers in older women with sarcopenic obesity: A randomized, double-blind, placebo-controlled trial. Clin Nutr ESPEN 2019; 32: 88-95

13. Hoffman JR, Ratamess NA, Kang J, et al. Effects of protein supplementation on muscular performance and resting hormonal changes in college football players. J Sports Sci Med 2007; 6: 85-92

14. Hulmi JJ, Kovanen V, Selänne, H, et al. Acute and long-term effects of resistance exercise with or without protein ingestion on muscle hypertrophy and gene expression. Amino Acids 2009; 37: 297-308

15. Verdijk LB, Jonkers RA, Gleeson BG, et al. Protein supplementation before and after exercise does not further augment skeletal muscle hypertrophy after resistance training in elderly men. Am J Clin Nutr 2009; 89: 608-616

16. Farup J, Rahbek SK, Vendelbo MH, et al. Whey protein hydrolysate augments tendon and muscle hypertrophy independent of resistance exercise contraction mode. Scand J Med Sci Sports 2014; 24: 788-798

17. Hulmi JJ, Laakso M, Mero AA, et al. The effects of whey protein with or without carbohydrates on resistance training adaptations. J Int Soc Sports Nutr 2015; 12: 48

18. Reidy PT, Borack MS, Markofski MM, et al. Protein supplementation has minimal effects on muscle adaptations during resistance exercise training in young men: a double-blind randomized clinical trial. J Nutr 2016; 146: 1660-1669

19. Taylor LW, Wilborn C, Roberts MD, et al. Eight weeks of pre- and postexercise whey protein supplementation increases lean body mass and improves performance in Division III collegiate female basketball players. Appl Physiol Nutr Metab 2016; 41: 249-254

20. Rossato LT, Nahas PC, de Branco FMS, et al. Higher protein intake does not improve lean mass gain when compared with RDA recommendation in postmenopausal women following resistance exercise protocol: a randomized clinical trial. Nutrients 2017; 9: 1007

21. Orsatti FL, Maestá N, de Oliveira EP, et al. Adding soy protein to milk enhances the effect of resistance training on muscle strength in postmenopausal women. J Diet Suppl 2018; 15: 140-152

22. Jonvik KL, Paulussen KJM, Danen SL, et al. Protein supplementation does not augment adaptations to endurance exercise training. Med Sci Sports Exerc 2019; 51: 2041-2049

23. Weisgarber KD, Candow DG, Vogt ES. Whey protein before and during resistance exercise has no effect on muscle mass and strength in untrained young adults. Int J Sport Nutr Exerc Metab 2012; 22: 463-469

24. Sharp MH, Lowery RP, Shields KA, et al. The effects of beef, chicken, or whey protein after workout on body composition and muscle performance. J Strength Cond Res 2018; 32: 2233-2242

25. Iglay HB, Thyfault JP, Apolzan JW, et al. Resistance training and dietary protein: effects on glucose tolerance and contents of skeletal muscle insulin signaling proteins in older persons. Am J Clin Nutr 2007; 85: 1005-1013

26. Snijders T, Res PT, Smeets JS, et al. Protein ingestion before sleep increases muscle mass and strength gains during Prolonged resistance-type exercise training in healthy young men. J Nutr 2015; 145: 1178-1184

27. Gaffney KA, Lucero A, Stoner L, et al. Nil whey protein effect on glycemic control after intense mixed-mode training in type 2 diabetes. Med Sci Sports Exerc 2018; 50: 11-17
