# Supplementary material for: Synergistic Effect of Increased Total Protein Intake and Strength Training on Muscle Strength: A Dose-Response Meta-analysis of Randomized Controlled Trials
Source: Sports Med Open. 2022 Sep 4;8:110. doi: 10.1186/s40798-022-00508-w (PMC9441410; doi:10.1186/s40798-022-00508-w)
Supplement: Supplementary file 3 — Additional file 3. Summary of characteristics of included studies, Summary of nutrition surveys, Summary of assigned protein amounts and differences between groups, Summary of conditions of the studies’ interventions. [file 40798_2022_508_MOESM3_ESM.docx]

**Additional file 3**

**Synergistic effect of increased total protein intake and strength training on muscle strength:**

**A dose–response meta-analysis from randomized controlled trials**

Ryoichi Tagawa^1 #^

Daiki Watanabe^2, 3 #^

Kyoko Ito^1^

Takeru Otsuyama^1^

Kyosuke Nakayama^1^

Chiaki Sanbongi^1^

Motohiko Miyachi^2, 3 *^

^1^ Nutrition and Food Function Research Department, Food Microbiology and Function Research Laboratories, R&D Division, Meiji Co., Ltd., 1-29-1 Nanakuni, Hachioji, Tokyo 192-0919, Japan

^2^ Faculty of Sport Sciences, Waseda University, 2-579-15 Mikajima, Tokorozawa-city, Saitama 359-1192, Japan

^3^ Department of Physical Activity Research, National Institute of Health and Nutrition, National Institutes of Biomedical Innovation, Health and Nutrition, 1-23-1 Toyama, Shinjuku-ku, Tokyo 162-8636, Japan

# RT and DW equally contributed to the manuscript and are joint first authors.

* Corresponding Author: Motohiko Miyachi

Email: cardiovascular0327@mac.com

*Supplementary Table S3* **Summary of characteristics of included studies**

|  | **Percent male** | | **Race with largest number** | | **Age** | | **Height** | | **Weight** | | **BMI** | | **Health state** | | **Frequency of exercise before intervention** | |
| --- | --- | --- | --- | --- | --- | --- | --- | --- | --- | --- | --- | --- | --- | --- | --- | --- |
| **Author and Year** | **(%)** |  | | **(years)** | | **(cm)** | | **(kg)** | | **(kg/m^2^)** | |  | | **Resistance** | | **Other** |
| **Castaneda (1995) [1]** | **0** | **Caucasian** | | **72.0** | | **158.8** | | **66.0** | | **26.4** | | **Healthy** | | **Unclear** | | **Low** |
| **Rankin (2004) [2]*** | **100** | **Caucasian** | | **20.5** | | **179.7** | | **78.0** | | **24.2** | | **Healthy** | | **Low** | | **Unclear** |
| **Candow (2006) (Post-RT) [3]*** | **100** | **Caucasian** | | **66.5** | | **173.0** | | **85.3** | | **28.5** | | **Healthy** | | **Low** | | **Unclear** |
| **Candow (2006) (Pre-RT) [3]*** | **100** | **Caucasian** | | **63.3** | | **176.0** | | **87.5** | | **28.2** | | **Healthy** | | **Low** | | **Unclear** |
| **Candow (2006) (Soy) [4]*** | **33** | **Caucasian** | | **22.5** | | **169.3** | | **71.8** | | **25.1** | | **Healthy** | | **Low** | | **Unclear** |
| **Candow (2006) (Whey) [4]*** | **33** | **Caucasian** | | **24.0** | | **170.5** | | **69.3** | | **23.8** | | **Healthy** | | **Low** | | **Unclear** |
| **Kerksick (2006) [5]*** | **100** | **Caucasian** | | **31.0** | | **179.2** | | **84.0** | | **26.2** | | **Healthy** | | **High** | | **Unclear** |
| **Cribb (2007) [6]*** | **100** | **Others** | | **24.0** | | **181.0** | | **70.0** | | **21.4** | | **Healthy** | | **High** | | **Unclear** |
| **Hartman (2007) (Milk) [7]*** | **100** | **Caucasian** | | **-** | | **177.0** | | **78.8** | | **25.1** | | **Healthy** | | **Low** | | **High** |
| **Hartman (2007)**  **(Soy) [7]*** | **100** | **Caucasian** | | **-** | | **179.0** | | **83.3** | | **26.0** | | **Healthy** | | **Low** | | **High** |
| **Hoffman (2007) [8]*** | **100** | **Caucasian** | | **20.3** | | **182.0** | | **93.9** | | **28.3** | | **Healthy** | | **High** | | **Athlete** |
| **Iglay (2007) [9]*** | **44** | **Caucasian** | | **61.0** | | **171.1** | | **78.2** | | **26.7** | | **Healthy** | | **Low** | | **Unclear** |
| **Hoffman (2009) (AM/PM) [10]*** | **100** | **Caucasian** | | **19.6** | | **183.4** | | **102.3** | | **30.4** | | **Healthy** | | **High** | | **Athlete** |
| **Hoffman (2009) (Pre/Post-RT) [10]*** | **100** | **Caucasian** | | **19.9** | | **183.4** | | **95.1** | | **28.3** | | **Healthy** | | **High** | | **Athlete** |
| **Hulmi (2009) [11]*** | **100** | **Causcasian** | | **25.2** | | **182.2** | | **76.1** | | **22.9** | | **Healthy** | | **Low** | | **High** |
| **Shinkai (2009) (Fallers) [12]*** | **18** | **Japanese** | | **78.3** | | **152.9** | | **50.6** | | **22.1** | | **Healthy** | | **Unclear** | | **Unclear** |
| **Shinkai (2009) (Geriatric syndromes) [12]*** | **20** | **Japanese** | | **79.1** | | **148.8** | | **46.3** | | **21.0** | | **Frailty or Sarcopenia** | | **Low** | | **Low** |
| **Verdijk (2009) [13]*** | **100** | **Caucasian** | | **72.0** | | **173.0** | | **79.2** | | **26.5** | | **Healthy** | | **Low** | | **Low** |
| **Bemben (2010) [14]*** | **100** | **Caucasian** | | **58.2** | | **175.6** | | **88.3** | | **28.6** | | **Healthy** | | **Low** | | **Unclear** |
| **Josse (2010) [15]*** | **0** | **Caucasian** | | **23.2** | | **165.8** | | **72.0** | | **26.2** | | **Healthy** | | **Low** | | **High** |
| **Arazi (2011) [16]** | **100** | **Others** | | **21.3** | | **174.0** | | **73.0** | | **24.1** | | **Healthy** | | **High** | | **Unclear** |
| **Deibert (2011) [17]** | **100** | **Caucasian** | | **55.9** | | **178.0** | | **91.2** | | **28.4** | | **Healthy** | | **Low** | | **Low** |
| **Alemán-Mateo (2012) [18]** | **40** | **Others** | | **75.4** | | **161.4** | | **68.7** | | **26.5** | | **Frailty or Sarcopenia** | | **Unclear** | | **Unclear** |
| **Erskine (2012) [19]*** | **100** | **Caucasian** | | **23.1** | | **175.9** | | **74.9** | | **24.2** | | **Healthy** | | **Low** | | **Unclear** |
| **Farnfield (2012)**  **(Old) [20]*** | **100** | **Caucasian** | | **68.1** | | **173.1** | | **82.6** | | **27.5** | | **Healthy** | | **Low** | | **Unclear** |
| **Farnfield (2012) (Young) [20]*** | **100** | **Caucasian** | | **20.5** | | **178.5** | | **70.8** | | **22.2** | | **Healthy** | | **Low** | | **Unclear** |
| **Hida (2012) [21]*** | **0** | **Japanese** | | **-** | | **165.1** | | **60.6** | | **22.2** | | **Healthy** | | **Unclear** | | **Athlete** |
| **Tieland (2012) [22]*** | **41** | **Caucasian** | | **78.0** | | **165.0** | | **73.9** | | **27.0** | | **Frailty or Sarcopenia** | | **Low** | | **Low** |
| **Tieland (2012) [23]*** | **35** | **Caucasian** | | **78.0** | | **166.0** | | **79.5** | | **28.7** | | **Frailty or Sarcopenia** | | **Unclear** | | **Unclear** |
| **Weinheimer (2012) (20g) [24]*** | **41** | **Caucasian** | | **47.0** | | **170.0** | | **87.8** | | **30.4** | | **Healthy** | | **Low** | | **Sedentary** |
| **Weinheimer (2012) (40g) [24]*** | **32** | **Caucasian** | | **46.0** | | **170.0** | | **84.7** | | **29.4** | | **Healthy** | | **Low** | | **Sedentary** |
| **Weinheimer (2012) (60g) [24]*** | **40** | **Caucasian** | | **50.0** | | **170.0** | | **89.4** | | **30.7** | | **Healthy** | | **Low** | | **Sedentary** |
| **Weisgarber (2012) [25]*** | **56** | **Caucasian** | | **24.5** | | **172.5** | | **88.3** | | **29.7** | | **Healthy** | | **Low** | | **Unclear** |
| **Arnarson (2013) [26]*** | **42** | **Caucasian** | | **73.3** | | **169.0** | | **81.0** | | **28.1** | | **High risk of metabolic syndrome** | | **Unclear** | | **Unclear** |
| **Chalé (2013) [27]*** | **40** | **Caucasian** | | **78.0** | | **164.2** | | **73.0** | | **27.0** | | **Healthy** | | **Low** | | **Low** |
| **Herda (2013) [28]*** | **100** | **Caucasian** | | **21.0** | | **180.2** | | **76.5** | | **23.6** | | **Healthy** | | **High** | | **High** |
| **Leenders (2013) (Female) [29]*** | **0** | **Caucasian** | | **72.0** | | **162.0** | | **63.3** | | **24.2** | | **Healthy** | | **Low** | | **High** |
| **Leenders (2013) (Male) [29]*** | **100** | **Caucasian** | | **70.0** | | **176.0** | | **84.0** | | **27.2** | | **Healthy** | | **Low** | | **High** |
| **Volek (2013) (Soy) [30]*** | **50** | **Caucasian** | | **24.0** | | **170.5** | | **72.0** | | **24.8** | | **Healthy** | | **Low** | | **Unclear** |
| **Volek (2013) (Whey) [30]*** | **68** | **Caucasian** | | **22.8** | | **171.8** | | **74.1** | | **25.1** | | **Healthy** | | **Low** | | **Unclear** |
| **Alemán-Mateo (2014) [31]** | **50** | **Others** | | **70.8** | | **160.0** | | **70.7** | | **26.9** | | **Healthy** | | **Unclear** | | **Unclear** |
| **Babault (2014) (Casein) [32]** | **100** | **Caucasian** | | **22.2** | | **181.0** | | **77.7** | | **23.7** | | **Healthy** | | **Low** | | **Low** |
| **Babault (2014) (Whey) [32]** | **100** | **Caucasian** | | **22.5** | | **177.0** | | **70.8** | | **22.7** | | **Healthy** | | **Low** | | **Low** |
| **Farup (2014) [33]** | **100** | **Caucasian** | | **23.7** | | **182.1** | | **78.3** | | **23.6** | | **Healthy** | | **Low** | | **High** |
| **Figueroa (2014) (Casein) [34]** | **0** | **Caucasian** | | **31.0** | | **162.0** | | **102.4** | | **37.9** | | **Healthy** | | **Low** | | **Low** |
| **Figueroa (2014) (Whey) [34]** | **0** | **Caucasian** | | **28.0** | | **168.0** | | **95.5** | | **34.3** | | **Healthy** | | **Low** | | **Low** |
| **Gryson (2014) [35]** | **100** | **Caucasian** | | **60.9** | | **175.1** | | **82.1** | | **26.8** | | **Healthy** | | **Low** | | **Low** |
| **Mori (2014) [36]*** | **25** | **Japanese** | | **66.1** | | **158.5** | | **57.2** | | **22.8** | | **Healthy** | | **Unclear** | | **Unclear** |
| **Negro (2014) [38]*** | **67** | **Caucasian** | | **23.7** | | **170.0** | | **67.8** | | **23.5** | | **Healthy** | | **Low** | | **Low** |
| **Babaul (2015) (Pea) [38]** | **100** | **Caucasian** | | **22.0** | | **-** | | **-** | | **23.1** | | **Healthy** | | **Low** | | **Low** |
| **Babaul (2015) (Whey) [38]** | **100** | **Caucasian** | | **22.1** | | **-** | | **-** | | **23.0** | | **Healthy** | | **Low** | | **Low** |
| **Hulmi (2015) [39]*** | **100** | **Caucasian** | | **31.4** | | **181.0** | | **83.8** | | **25.6** | | **Healthy** | | **Low** | | **High** |
| **Ormsbee (2015) (Casein) [40]*** | **0** | **Caucasian** | | **30.0** | | **166.1** | | **100.8** | | **36.5** | | **Healthy** | | **Low** | | **Low** |
| **Ormsbee (2015) (Whey) [40]*** | **0** | **Caucasian** | | **29.3** | | **165.7** | | **94.5** | | **34.4** | | **Healthy** | | **Low** | | **Low** |
| **Snijders (2015) [41]*** | **100** | **Caucasian** | | **23.0** | | **182.0** | | **76.9** | | **23.2** | | **Healthy** | | **Unclear** | | **High** |
| **Zhu (2015) [42]*** | **0** | **Caucasian** | | **74.2** | | **159.7** | | **66.8** | | **26.1** | | **Healthy** | | **Unclear** | | **Unclear** |
| **Maltais (2016) [43]*** | **100** | **Caucasian** | | **68.0** | | **172.4** | | **76.7** | | **25.8** | | **Frailty or Sarcopenia** | | **Low** | | **Low** |
| **Paoli (2016) [44]*** | **100** | **Caucasian** | | **24.9** | | **182.0** | | **79.5** | | **24.0** | | **Healthy** | | **Low** | | **High** |
| **Reidy (2016) (Blend) [45]*** | **100** | **Caucasian** | | **24.0** | | **179.0** | | **78.0** | | **24.4** | | **Healthy** | | **Low** | | **Low** |
| **Reidy (2016) (Whey) [45]*** | **100** | **Caucasian** | | **24.0** | | **178.0** | | **81.8** | | **25.8** | | **Healthy** | | **Low** | | **Low** |
| **Taylor (2016) [46]*** | **0** | **Caucasian** | | **20.0** | | **170.0** | | **66.0** | | **22.8** | | **Healthy** | | **Unclear** | | **Athlete** |
| **Thomson (2016) (Dairy) [47]*** | **46** | **Caucasian** | | **61.3** | | **169.3** | | **79.4** | | **27.7** | | **Healthy** | | **Low** | | **Low** |
| **Thomson (2016)**  **(Soy) [47]*** | **45** | **Caucasian** | | **61.7** | | **169.5** | | **79.0** | | **27.5** | | **Healthy** | | **Low** | | **Low** |
| **Hwang (2017) [48]*** | **100** | **Caucasian** | | **21.0** | | **178.9** | | **80.5** | | **25.1** | | **Healthy** | | **High** | | **Unclear** |
| **Niccoli (2017) [49]*** | **32** | **Caucasian** | | **81.8** | | **165.0** | | **66.0** | | **24.2** | | **Frailty or Sarcopenia** | | **Unclear** | | **Unclear** |
| **Ottestad (2017) [50]*** | **29** | **Caucasian** | | **76.9** | | **167.6** | | **77.5** | | **27.6** | | **Frailty or Sarcopenia** | | **Unclear** | | **Unclear** |
| **Rossato (2017) [51]*** | **0** | **Others** | | **63.4** | | **155.0** | | **67.6** | | **28.1** | | **Healthy** | | **Low** | | **Low** |
| **Vorup (2017) [52]*** | **46** | **Caucasian** | | **69.0** | | **167.0** | | **72.5** | | **26.0** | | **Healthy** | | **Low** | | **Low** |
| **Bhasin (2018) [53]*** | **100** | **Caucasian** | | **73.5** | | **174.2** | | **90.2** | | **29.6** | | **Healthy** | | **Unclear** | | **Low** |
| **Mitchell (2018) [54]*** | **100** | **Caucasian** | | **51.5** | | **177.5** | | **87.0** | | **27.5** | | **Healthy** | | **Unclear** | | **Unclear** |
| **Nobuco (2018) (Post-RT) [55]*** | **0** | **Others** | | **66.2** | | **161.0** | | **65.4** | | **25.3** | | **Healthy** | | **Unclear** | | **Unclear** |
| **Nobuco (2018) (Pre-RT) [55]*** | **0** | **Others** | | **67.5** | | **162.0** | | **69.0** | | **26.4** | | **Healthy** | | **Unclear** | | **Unclear** |
| **Orsatti (2018) [56]*** | **0** | **Others** | | **56.8** | | **160.0** | | **68.4** | | **27.5** | | **Healthy** | | **Low** | | **Low** |
| **Park (2018) (1.2g/kg) [57]*** | **35** | **Others** | | **77.3** | | **156.0** | | **59.7** | | **24.4** | | **Frailty or Sarcopenia** | | **Unclear** | | **Unclear** |
| **Park (2018) (1.5g/kg) [57]*** | **30** | **Others** | | **76.8** | | **154.0** | | **56.3** | | **23.7** | | **Frailty or Sarcopenia** | | **Unclear** | | **Unclear** |
| **Sharp (2018)**  **(Beef) [58]*** | **50** | **Caucasian** | | **22.0** | | **170.0** | | **70.1** | | **24.3** | | **Healthy** | | **High** | | **Unclear** |
| **Sharp (2018) (Chicken) [58]*** | **45** | **Caucasian** | | **21.0** | | **169.0** | | **74.5** | | **26.1** | | **Healthy** | | **High** | | **Unclear** |
| **Sharp (2018)**  **(Whey) [58]*** | **50** | **Caucasian** | | **19.0** | | **171.5** | | **74.6** | | **25.4** | | **Healthy** | | **High** | | **Unclear** |
| **Sugihara (2018) [59]*** | **0** | **Others** | | **67.4** | | **154.8** | | **61.3** | | **25.6** | | **Healthy** | | **High** | | **Unclear** |
| **Bartholomae (2019) [60]*** | **8** | **Caucasian** | | **31.2** | | **165.3** | | **65.5** | | **24.0** | | **Healthy** | | **Low** | | **Low** |
| **Centner (2019) [61]** | **100** | **Caucasian** | | **61.7** | | **177.0** | | **84.9** | | **27.0** | | **Healthy** | | **Low** | | **Low** |
| **Drotningsvik (2019) [62]*** | **33** | **Caucasian** | | **84.0** | | **-** | | **69.4** | | **-** | | **Frailty or Sarcopenia** | | **Unclear** | | **Unclear** |
| **Jonvik (2019) [63]*** | **100** | **Caucasian** | | **26.0** | | **183.0** | | **79.7** | | **23.8** | | **Healthy** | | **Low** | | **Unclear** |
| **Nabuco (2018) [64]*** | **0** | **Others** | | **68.0** | | **153.0** | | **61.6** | | **26.4** | | **Frailty or Sarcopenia** | | **High** | | **Low** |
| **Nahas (2019) [65]*** | **0** | **Others** | | **64.7** | | **155.0** | | **67.1** | | **27.5** | | **Healthy** | | **Low** | | **Unclear** |
| **ten Haaf (2019) [66]*** | **81** | **Caucasian** | | **69.0** | | **176.4** | | **84.6** | | **27.2** | | **Healthy** | | **Low** | | **High** |
| **Atherton (2020) [67]*** | **100** | **Caucasian** | | **70.5** | | **-** | | **90.1** | | **-** | | **Healthy** | | **Low** | | **High** |
| **Björkman (2020) [68]** | **30** | **Caucasian** | | **83.6** | | **-** | | **-** | | **25.3** | | **Frailty or Sarcopenia** | | **Unclear** | | **Unclear** |
| **Duarte (2020) [69]*** | **-** | **Caucasian** | | **25.0** | | **171.0** | | **69.7** | | **23.8** | | **Healthy** | | **High** | | **Unclear** |
| **Dulac (2020) (Casein) [70]*** | **100** | **Caucasian** | | **69.0** | | **171.1** | | **76.1** | | **26.0** | | **Healthy** | | **Low** | | **Low** |
| **Dulac (2020) (Whey) [70]*** | **100** | **Caucasian** | | **68.3** | | **170.9** | | **78.0** | | **26.7** | | **Healthy** | | **Low** | | **Low** |
| **Forbes (2020) (WPC Female) [71]*** | **0** | **Caucasian** | | **27.0** | | **169.9** | | **65.1** | | **22.6** | | **Healthy** | | **High** | | **High** |
| **Forbes (2020) (WPC Male) [71]*** | **100** | **Caucasian** | | **26.0** | | **179.0** | | **77.6** | | **24.2** | | **Healthy** | | **High** | | **High** |
| **Forbes (2020) (WPI Female) [71]*** | **0** | **Caucasian** | | **27.0** | | **169.9** | | **65.4** | | **22.7** | | **Healthy** | | **High** | | **High** |
| **Forbes (2020) (WPI Male) [71]*** | **100** | **Caucasian** | | **26.0** | | **179.0** | | **78.9** | | **24.6** | | **Healthy** | | **High** | | **High** |
| **Krull (2020) [72]*** | **55** | **Others** | | **33.0** | | **-** | | **81.4** | | **-** | | **Healthy** | | **Unclear** | | **Unclear** |
| **Kim(2021)(Evening)[73]*** | **0** | **Japanese** | | **70.3** | | **156.0** | | **52.4** | | **21.7** | | **Healthy** | | **Low** | | **Low** |
| **Kim(2021)(Morning)[73]*** | **0** | **Japanese** | | **69.0** | | **155.0** | | **55.7** | | **23.3** | | **Healthy** | | **Low** | | **Low** |
| **Griffen (2022) (with RT) [74]*** | **100** | **Caucasian** | | **68.0** | | **174.0** | | **80.9** | | **26.6** | | **Healthy** | | **Low** | | **Unclear** |
| **Griffen (2022) (without RT) [74]*** | **100** | **Caucasian** | | **66.0** | | **176.0** | | **78.0** | | **25.0** | | **Healthy** | | **Low** | | **Unclear** |
| **Sexton(2021)(Female)[75]*** | **0** | **Caucasian** | | **22.0** | | **171.0** | | **66.6** | | **22.8** | | **Healthy** | | **Low** | | **Unclear** |
| **Sexton(2021)(Male)[75]*** | **100** | **Caucasian** | | **21.0** | | **177.0** | | **76.0** | | **24.3** | | **Healthy** | | **Low** | | **Unclear** |
| **Ullevig(2021)[76]*** | **0** | **Caucasian** | | **72.9** | | **156.2** | | **80.0** | | **32.8** | | **Frailty or Sarcopenia** | | **Unclear** | | **Unclear** |
| **McKenna(2021)[77]*** | **50** | **Caucasian** | | **49.0** | | **172.0** | | **81.3** | | **27.6** | | **Healthy** | | **Low** | | **Unclear** |
| **Li(2021)(Blend)[78]*** | **55** | **Others** | | **70.0** | | **162.0** | | **54.1** | | **20.6** | | **Healthy** | | **Unclear** | | **Unclear** |
| **Li(2021)(Soy)[78]*** | **48** | **Others** | | **69.0** | | **160.0** | | **54.0** | | **21.2** | | **Healthy** | | **Unclear** | | **Unclear** |
| **Li(2021)(Whey)[78]*** | **52** | **Others** | | **71.0** | | **159.0** | | **54.9** | | **21.8** | | **Healthy** | | **Unclear** | | **Unclear** |
| **Mertz(2021)(Collagen)[79]*** | **54** | **Caucasian** | | **70.4** | | **172.0** | | **75.1** | | **25.4** | | **Healthy** | | **Low** | | **Unclear** |
| **Mertz(2021)(Whey)[79]*** | **56** | **Caucasian** | | **70.3** | | **173.0** | | **75.0** | | **25.2** | | **Healthy** | | **Low** | | **Unclear** |
| **Nygård(2021)[80]*** | **33** | **Caucasian** | | **73.4** | | **168.0** | | **72.2** | | **25.5** | | **Healthy** | | **Low** | | **Unclear** |
| **Lamb(2020)[81]*** | **60** | **Caucasian** | | **60.0** | | **171.7** | | **84.9** | | **27.8** | | **Healthy** | | **Low** | | **Unclear** |
| **Boutry-Regard(2020)[82]** | **20** | **Japanese** | | **78.0** | | **153.0** | | **49.7** | | **21.3** | | **Frailty or Sarcopenia** | | **Unclear** | | **Unclear** |
|  | | | | | | | | | | | | | | | | |

**All trials were used to create a forest plot to evaluate the effect of added protein intakes on muscle strength percentage changes compared with control groups; Studies marked with asterisk were also used to create spline models to evaluate the relationship between total protein intake and muscle strength percentage changes from baselines.**

*Supplementary Table S4* **Summary of nutrition surveys**

|  | **Protein intake** | | | | | | **Energy intake** | | | | | |
| --- | --- | --- | --- | --- | --- | --- | --- | --- | --- | --- | --- | --- |
|  | **before intervention** | | **during intervention**  **(not include supplementation)** | | **during intervention (include supplementation)** | | **before intervention** | | **during intervention**  **(not include supplementation)** | | **during intervention (include supplementation)** | |
| **Author and Year** | **(g/kg /day)** | **(g/day)** | **(g/kg /day)** | **(g/day)** | **(g/kg /day)** | **(g/day)** | **(kcal/kg /day)** | **(kcal/day)** | **(kcal/kg /day)** | **(kcal/day)** | **(kcal/kg /day)** | **(kcal/day)** |
| **Castaneda (1995) [1]** | **-** | **-** | **-** | **-** | **-** | **-** | **27.6** | **1819** | **-** | **-** | **-** | **-** |
| **Rankin (2004) [2]*** | **1.20** | **93.6** | **1.30** | **101.4** | **1.39** | **108.4** | **31.9** | **2488** | **34.4** | **2683** | **-** | **-** |
| **Candow (2006) (Post-RT) [3]*** | **-** | **-** | **-** | **-** | **1.26** | **107.5** | **-** | **-** | **-** | **-** | **28.8** | **2453** |
| **Candow (2006) (Pre-RT) [3]*** | **-** | **-** | **-** | **-** | **1.40** | **122.5** | **-** | **-** | **-** | **-** | **26.6** | **2330** |
| **Candow (2006) (Soy) [4]*** | **-** | **-** | **-** | **-** | **1.80** | **129.2** | **-** | **-** | **-** | **-** | **41.7** | **2997** |
| **Candow (2006) (Whey) [4]*** | **-** | **-** | **-** | **-** | **1.75** | **121.3** | **-** | **-** | **-** | **-** | **44.3** | **3071** |
| **Kerksick (2006) [5]*** | **2.10** | **176.4** | **-** | **-** | **2.38** | **199.5** | **30.8** | **2587** | **-** | **-** | **34.4** | **2892** |
| **Cribb (2007) [6]*** | **2.10** | **176.4** | **1.50** | **126.0** | **2.69** | **225.6** | **40.8** | **3427** | **39.9** | **3352** | **-** | **-** |
| **Hartman (2007) (Milk) [7]*** | **1.60** | **112.0** | **1.65** | **115.5** | **2.95** | **206.5** | **41.6** | **2912** | **39.8** | **2786** | **-** | **-** |
| **Hartman (2007) (Soy) [7]*** | **1.40** | **111.0** | **1.80** | **147.5** | **2.12** | **172.5** | **38.0** | **3011** | **39.2** | **3191** | **-** | **-** |
| **Hoffman (2007) [8]*** | **1.20** | **105.0** | **1.65** | **142.0** | **1.95** | **167.0** | **37.5** | **3107** | **35.6** | **2976** | **-** | **-** |
| **Iglay (2007) [9]*** | **-** | **-** | **-** | **-** | **2.00** | **188.0** | **-** | **-** | **-** | **-** | **32.7** | **3072** |
| **Hoffman (2009) (AM/PM) [10]*** | **1.40** | **144.0** | **-** | **-** | **2.28** | **229.0** | **28.4** | **2860** | **-** | **-** | **28.6** | **2862** |
| **Hoffman (2009) (Pre/Post-RT) [10]*** | **1.80** | **175.0** | **-** | **-** | **2.16** | **204.0** | **32.3** | **3060** | **-** | **-** | **28.6** | **2738** |
| **Hulmi (2009) [11]*** | **1.40** | **106.5** | **1.60** | **121.8** | **1.71** | **130.3** | **32.6** | **2486** | **34.1** | **2474** | **-** | **-** |
| **Shinkai (2009) (Fallers) [12]*** | **1.70** | **86.0** | **1.84** | **93.0** | **1.93** | **97.6** | **40.9** | **2068** | **42.1** | **2131** | **-** | **-** |
| **Shinkai (2009) (Geriatric syndromes) [12]*** | **1.97** | **91.0** | **1.92** | **89.0** | **2.02** | **93.6** | **46.7** | **2163** | **45.8** | **2120** | **-** | **-** |
| **Verdijk (2009) [13]*** | **1.10** | **87.1** | **1.10** | **87.1** | **1.21** | **95.7** | **28.1** | **2223** | **28.4** | **2247** | **-** | **-** |
| **Bemben (2010) [14]*** | **0.95** | **84.1** | **-** | **-** | **1.04** | **85.6** | **22.8** | **2010** | **-** | **-** | **20.8** | **1835** |
| **Josse (2010) [15]*** | **0.97** | **70.0** | **-** | **-** | **1.25** | **90.0** | **29.5** | **2125** | **-** | **-** | **31.7** | **2280** |
| **Arazi (2011) [16]** | **-** | **-** | **-** | **-** | **-** | **-** | **-** | **-** | **-** | **-** | **-** | **-** |
| **Deibert (2011) [17]** | **-** | **-** | **-** | **-** | **-** | **-** | **-** | **-** | **-** | **-** | **-** | **-** |
| **Alemán-Mateo (2012) [18]** | **-** | **-** | **-** | **-** | **-** | **-** | **-** | **-** | **-** | **-** | **-** | **-** |
| **Erskine (2012) [19]*** | **1.28** | **95.3** | **1.33** | **100.0** | **1.56** | **117.0** | **33.1** | **2482** | **33.1** | **2480** | **34.3** | **2572** |
| **Farnfield (2012) (Old) [20]*** | **-** | **-** | **-** | **-** | **1.55** | **128.0** | **-** | **-** | **-** | **-** | **-** | **-** |
| **Farnfield (2012) (Young) [20]*** | **-** | **-** | **-** | **-** | **1.85** | **131.0** | **-** | **-** | **-** | **-** | **-** | **-** |
| **Hida (2012) [21]*** | **1.08** | **65.4** | **-** | **-** | **1.23** | **74.5** | **35.4** | **2147** | **-** | **-** | **33.0** | **1997** |
| **Tieland (2012) [22]*** | **1.00** | **78.0** | **1.05** | **71.0** | **1.46** | **101.0** | **26.2** | **1936** | **23.9** | **1769** | **-** | **-** |
| **Tieland (2012) [23]*** | **1.00** | **77.7** | **1.00** | **78.9** | **1.30** | **103.4** | **25.9** | **2055** | **24.4** | **1936** | **-** | **-** |
| **Weinheimer (2012) (20g) [24]*** | **0.99** | **87.0** | **0.90** | **78.5** | **1.13** | **98.5** | **24.8** | **2175** | **20.8** | **1828** | **25.2** | **2211** |
| **Weinheimer (2012) (40g) [24]*** | **1.12** | **94.0** | **0.96** | **82.0** | **1.43** | **122.0** | **27.9** | **2366** | **23.1** | **1960** | **27.8** | **2354** |
| **Weinheimer (2012) (60g) [24]*** | **0.97** | **86.0** | **0.93** | **81.5** | **1.63** | **141.5** | **24.9** | **2223** | **20.9** | **1864** | **25.3** | **2259** |
| **Weisgarber (2012) [25]*** | **-** | **-** | **-** | **-** | **1.37** | **121.2** | **-** | **-** | **-** | **-** | **24.7** | **2183** |
| **Arnarson (2013) [26]*** | **1.00** | **81.0** | **-** | **-** | **1.06** | **85.9** | **21.4** | **1724** | **-** | **-** | **22.1** | **1787** |
| **Chalé (2013) [27]*** | **0.97** | **71.0** | **0.88** | **64.0** | **1.22** | **89.0** | **22.5** | **1639** | **20.1** | **1464** | **23.3** | **1702** |
| **Herda (2013) [28]*** | **1.40** | **104.7** | **1.50** | **112.5** | **2.00** | **152.5** | **36.2** | **2767** | **37.5** | **2869** | **39.6** | **3029** |
| **Leenders (2013) (Female) [29]*** | **1.20** | **78.7** | **1.20** | **78.7** | **1.44** | **93.7** | **31.0** | **1960** | **31.7** | **2079** | **-** | **-** |
| **Leenders (2013) (Male) [29]*** | **1.10** | **92.6** | **1.00** | **84.2** | **1.18** | **99.2** | **28.5** | **2390** | **28.7** | **2414** | **-** | **-** |
| **Volek (2013) (Soy) [30]*** | **1.27** | **86.2** | **-** | **-** | **1.36** | **97.0** | **28.2** | **2032** | **-** | **-** | **28.4** | **2044** |
| **Volek (2013) (Whey) [30]*** | **1.27** | **92.5** | **-** | **-** | **1.37** | **101.1** | **28.5** | **2111** | **-** | **-** | **28.5** | **2109** |
| **Alemán-Mateo (2014) [31]** | **-** | **-** | **-** | **-** | **-** | **-** | **-** | **-** | **-** | **-** | **-** | **-** |
| **Babault (2014) (Casein) [32]** | **-** | **-** | **-** | **-** | **-** | **-** | **-** | **-** | **-** | **-** | **-** | **-** |
| **Babault (2014) (Whey) [32]** | **-** | **-** | **-** | **-** | **-** | **-** | **-** | **-** | **-** | **-** | **-** | **-** |
| **Farup (2014) [33]** | **-** | **-** | **-** | **-** | **-** | **-** | **-** | **-** | **-** | **-** | **-** | **-** |
| **Figueroa (2014) (Casein) [34]** | **-** | **-** | **-** | **-** | **-** | **-** | **-** | **-** | **-** | **-** | **-** | **-** |
| **Figueroa (2014) (Whey) [34]** | **-** | **-** | **-** | **-** | **-** | **-** | **-** | **-** | **-** | **-** | **-** | **-** |
| **Gryson (2014) [35]** | **-** | **-** | **-** | **-** | **-** | **-** | **-** | **-** | **-** | **-** | **-** | **-** |
| **Mori (2014) [36]*** | **0.91** | **51.7** | **0.95** | **54.3** | **1.06** | **60.7** | **33.8** | **1934** | **34.2** | **1959** | **-** | **-** |
| **Negro (2014) [37]*** | **1.00** | **67.8** | **-** | **-** | **1.13** | **76.4** | **-** | **-** | **-** | **-** | **-** | **-** |
| **Babault (2015) (Pea) [38]** | **-** | **-** | **-** | **-** | **-** | **-** | **-** | **-** | **-** | **-** | **-** | **-** |
| **Babault (2015) (Whey) [38]** | **-** | **-** | **-** | **-** | **-** | **-** | **-** | **-** | **-** | **-** | **-** | **-** |
| **Hulmi (2015) [39]*** | **1.50** | **125.7** | **-** | **-** | **1.62** | **135.7** | **29.6** | **2483** | **-** | **-** | **-** | **-** |
| **Ormsbee (2015) (Casein) [40]*** | **0.86** | **87.0** | **0.73** | **74.0** | **1.03** | **104.0** | **23.4** | **2360** | **18.5** | **1861** | **-** | **-** |
| **Ormsbee (2015) (Whey) [40]*** | **0.86** | **81.0** | **0.99** | **94.0** | **1.31** | **124.0** | **19.0** | **1796** | **20.1** | **1904** | **-** | **-** |
| **Snijders (2015) [41]*** | **1.30** | **99.0** | **1.40** | **106.0** | **1.90** | **146.1** | **19.4** | **2749** | **35.4** | **2725** | **-** | **-** |
| **Zhu (2015) [42]*** | **1.20** | **76.0** | **-** | **-** | **1.65** | **106.0** | **25.4** | **1697** | **-** | **-** | **-** | **-** |
| **Maltais (2016) [43]*** | **1.04** | **79.8** | **-** | **-** | **1.11** | **85.3** | **30.3** | **2326** | **-** | **-** | **-** | **-** |
| **Paoli (2016) [44]*** | **-** | **-** | **-** | **-** | **1.96** | **155.9** | **-** | **-** | **-** | **-** | **28.0** | **2227** |
| **Reidy (2016) (Blend) [45]*** | **1.33** | **101.0** | **-** | **-** | **1.61** | **125.5** | **31.5** | **2460** | **-** | **-** | **30.3** | **2365** |
| **Reidy (2016) (Whey) [45]*** | **1.29** | **102.0** | **-** | **-** | **1.59** | **130.5** | **30.4** | **2490** | **-** | **-** | **31.6** | **2585** |
| **Taylor (2016) [46]*** | **1.39** | **92.0** | **-** | **-** | **1.71** | **112.6** | **29.6** | **1951** | **-** | **-** | **-** | **-** |
| **Thomson (2016) (Dairy) [47]*** | **-** | **-** | **1.06** | **82.7** | **1.42** | **109.6** | **-** | **-** | **-** | **-** | **25.2** | **2004** |
| **Thomson (2016) (Soy) [47]*** | **-** | **-** | **1.08** | **81.3** | **1.45** | **108.8** | **-** | **-** | **-** | **-** | **24.5** | **1934** |
| **Hwang (2017) [48]*** | **1.26** | **101.7** | **1.31** | **105.5** | **1.52** | **121.9** | **23.9** | **1923** | **25.2** | **2028** | **-** | **-** |
| **Niccoli (2017) [49]*** | **-** | **-** | **-** | **-** | **1.48** | **97.7** | **-** | **-** | **-** | **-** | **24.3** | **1603** |
| **Ottestad (2017) [50]*** | **1.00** | **77.5** | **-** | **-** | **1.40** | **103.6** | **21.3** | **1649** | **-** | **-** | **24.1** | **1864** |
| **Rossato (2017) [51]*** | **0.82** | **53.2** | **-** | **-** | **1.18** | **77.4** | **20.9** | **1412** | **-** | **-** | **22.2** | **1502** |
| **Vorup (2017) [52]*** | **1.20** | **87.0** | **-** | **-** | **1.20** | **91.0** | **27.8** | **2018** | **-** | **-** | **25.3** | **2125** |
| **Bhasin (2018) [53]*** | **0.72** | **64.9** | **-** | **-** | **1.18** | **106.0** | **-** | **-** | **-** | **-** | **26.4** | **2378** |
| **Mitchell (2018) [54]*** | **1.10** | **93.6** | **-** | **-** | **1.27** | **112.7** | **28.0** | **2434** | **-** | **-** | **29.0** | **2522** |
| **Nobuco (2018) (Post-RT) [55]*** | **0.94** | **61.5** | **0.98** | **64.1** | **1.46** | **95.2** | **22.4** | **1465** | **23.3** | **1524** | **26.7** | **1743** |
| **Nobuco (2018) (Pre-RT) [55]*** | **0.92** | **63.5** | **0.96** | **65.9** | **1.38** | **95.2** | **22.9** | **1580** | **23.2** | **1597** | **26.4** | **1818** |
| **Orsatti (2018) [56]*** | **1.00** | **68.4** | **-** | **-** | **1.37** | **93.4** | **20.7** | **1416** | **-** | **-** | **-** | **-** |
| **Park (2018) (1.2g/kg) [57]*** | **0.77** | **45.2** | **-** | **-** | **1.18** | **69.9** | **20.4** | **1216** | **-** | **-** | **23.3** | **1392** |
| **Park (2018) (1.5g/kg) [57]*** | **0.80** | **44.8** | **-** | **-** | **1.37** | **76.4** | **21.8** | **1224** | **-** | **-** | **24.6** | **1386** |
| **Sharp (2018) (Beef) [58]*** | **-** | **-** | **-** | **-** | **2.20** | **154.3** | **-** | **-** | **-** | **-** | **35.4** | **2483** |
| **Sharp (2018) (Chicken) [58]*** | **-** | **-** | **-** | **-** | **2.10** | **156.5** | **-** | **-** | **-** | **-** | **34.3** | **2556** |
| **Sharp (2018) (Whey) [58]*** | **-** | **-** | **-** | **-** | **2.20** | **164.1** | **-** | **-** | **-** | **-** | **34.6** | **2581** |
| **Sugihara (2018) [59]*** | **0.85** | **52.7** | **-** | **-** | **1.40** | **89.3** | **24.4** | **1493** | **-** | **-** | **27.0** | **1678** |
| **Bartholomae (2019) [60]*** | **0.76** | **46.1** | **0.68** | **44.3** | **0.95** | **62.3** | **-** | **-** | **-** | **-** | **-** | **-** |
| **Centner (2019) [61]** | **-** | **-** | **-** | **-** | **-** | **-** | **-** | **-** | **-** | **-** | **-** | **-** |
| **Drotningsvik (2019) [62]*** | **0.98** | **68.0** | **-** | **-** | **1.05** | **73.2** | **22.0** | **1527** | **-** | **-** | **-** | **-** |
| **Jonvik (2019) [63]*** | **1.21** | **96.0** | **1.09** | **86.9** | **1.59** | **124.5** | **31.3** | **2495** | **-** | **-** | **32.3** | **2572** |
| **Nabuco (2018) [64]*** | **0.93** | **57.3** | **1.00** | **61.6** | **1.24** | **76.6** | **25.0** | **1539** | **25.7** | **1585** | **-** | **-** |
| **Nahas (2019) [65]*** | **0.76** | **49.2** | **-** | **-** | **1.17** | **72.0** | **19.0** | **1273** | **-** | **-** | **21.8** | **1463** |
| **ten Haaf (2019) [66]*** | **0.86** | **72.8** | **0.92** | **77.8** | **1.29** | **108.8** | **22.7** | **1919** | **21.8** | **1841** | **-** | **-** |
| **Atherton (2020) [67]*** | **0.60** | **58.8** | **-** | **-** | **0.79** | **75.9** | **-** | **-** | **-** | **-** | **-** | **-** |
| **Björkman (2020) [68]** | **1.10** | **-** | **-** | **-** | **-** | **-** | **-** | **1641** | **-** | **-** | **-** | **-** |
| **Duarte (2020) [69]*** | **-** | **-** | **1.32** | **92.0** | **1.74** | **121.6** | **-** | **-** | **23.4** | **1632** | **-** | **-** |
| **Dulac (2020) (Casein) [70]*** | **1.34** | **102.0** | **1.22** | **92.9** | **1.62** | **123.2** | **30.1** | **2292** | **31.7** | **2413** | **-** | **-** |
| **Dulac (2020) (Whey) [70]*** | **1.40** | **109.2** | **1.24** | **96.7** | **1.62** | **126.7** | **31.8** | **2483** | **28.2** | **2200** | **-** | **-** |
| **Forbes (2020) (WPC Female) [71]*** | **1.20** | **78.1** | **-** | **-** | **3.20** | **208.3** | **36.3** | **2361** | **-** | **-** | **44.8** | **2915** |
| **Forbes (2020) (WPC Male) [71]*** | **1.40** | **108.6** | **-** | **-** | **3.80** | **294.9** | **36.0** | **2792** | **-** | **-** | **45.6** | **3539** |
| **Forbes (2020) (WPI Female) [71]*** | **1.20** | **78.5** | **-** | **-** | **3.20** | **209.3** | **36.1** | **2361** | **-** | **-** | **44.6** | **2915** |
| **Forbes (2020) (WPI Male) [71]*** | **1.40** | **110.5** | **-** | **-** | **3.80** | **299.8** | **35.4** | **2792** | **-** | **-** | **44.9** | **3539** |
| **Krull (2020) [72]*** | **0.90** | **73.3** | **-** | **-** | **1.20** | **97.7** | **-** | **-** | **-** | **-** | **-** | **-** |
| **Kim(2021)**  **(Evening)**  **[73]*** | **1.05** | **55.0** | **-** | **-** | **-** | **-** | **32.8** | **1718** | **-** | **-** | **-** | **-** |
| **Kim(2021)**  **(Morning)**  **[73]*** | **1.36** | **75.5** | **-** | **-** | **-** | **-** | **36.0** | **2004** | **-** | **-** | **-** | **-** |
| **Griffen (2022) (with RT) [74]*** | **1.03** | **81.0** | **-** | **-** | **1.61** | **128.0** | **24.3** | **1964** | **-** | **-** | **27.2** | **2199** |
| **Griffen (2022) (without RT) [74]*** | **1.03** | **81.0** | **-** | **-** | **1.62** | **127.0** | **25.2** | **1964** | **-** | **-** | **25.6** | **1996** |
| **Sexton(2021)**  **(Female)[75]*** | **1.04** | **69.0** | **-** | **-** | **1.37** | **91.0** | **22.4** | **1495** | **-** | **-** | **24.1** | **1607** |
| **Sexton(2021)**  **(Male)[75]*** | **1.55** | **118.0** | **-** | **-** | **1.68** | **128.0** | **29.8** | **2262** | **-** | **-** | **32.0** | **2433** |
| **Ullevig(2021)**  **[76]*** | **0.80** | **60.6** | **-** | **-** | **1.06** | **81.0** | **18.8** | **1506** | **-** | **-** | **19.1** | **1525** |
| **McKenna**  **(2021)[77]*** | **1.12** | **91.0** | **-** | **-** | **-** | **-** | **27.3** | **2220** | **-** | **-** | **27.7** | **2250** |
| **Li(2021)**  **(Blend)[78]*** | **1.14** | **61.1** | **1.19** | **64.3** | **1.49** | **80.2** | **27.1** | **1465** | **27.9** | **1508** | **29.3** | **1583** |
| **Li(2021)**  **(Soy)[78]*** | **1.11** | **59.6** | **1.17** | **63.2** | **1.51** | **79.3** | **27.5** | **1483** | **27.6** | **1493** | **28.9** | **1563** |
| **Li(2021)**  **(Whey)[78]*** | **1.14** | **62.7** | **1.08** | **59.5** | **1.39** | **75.3** | **28.4** | **1558** | **26.1** | **1431** | **27.6** | **1513** |
| **Mertz(2021)**  **(Collagen)**  **[79]*** | **1.20** | **90.1** | **-** | **-** | **-** | **-** | **25.9** | **1948** | **-** | **-** | **-** | **-** |
| **Mertz(2021)**  **(Whey)[79]*** | **1.10** | **82.5** | **-** | **-** | **-** | **-** | **27.2** | **2038** | **-** | **-** | **-** | **-** |
| **Nygård(2021)**  **[80]*** | **1.10** | **79.4** | **-** | **-** | **-** | **-** | **26.4** | **1809** | **-** | **-** | **-** | **-** |
| **Lamb(2020)**  **[81]*** | **1.12** | **95.0** | **-** | **-** | **1.40** | **119.0** | **24.8** | **2106** | **-** | **-** | **25.7** | **2181** |
| **Boutry-Regard(2020)**  **[82]** | **-** | **-** | **-** | **-** | **-** | **-** | **-** | **-** | **-** | **-** | **-** | **-** |

**All trials were used to create a forest plot to evaluate the effect of added protein intakes on muscle strength percentage changes compared with control groups; Studies marked with asterisk were also used to create spline models to evaluate the relationship between total protein intake and muscle strength percentage changes from baselines.**

*Supplementary Table S5* **Summary of assigned protein amounts and differences between groups**

|  | **Assigned protein amount** | | | | | | | | **Difference in protein amount between groups** | | | | | | | | | |
| --- | --- | --- | --- | --- | --- | --- | --- | --- | --- | --- | --- | --- | --- | --- | --- | --- | --- | --- |
| **Author  and Year** | **(g/kg/ dose)** | **(g/ dose)** | **(g/kg/ exercise day)** | **(g/ exercise day)** | **(g/kg/ day)** | **(g/day)** | **(g/kg)** | **(g)** | **(g/kg/ dose)** | **(g/ dose)** | **(g/kg/ exercise day)** | **(g/ exercise day)** | **(g/kg/ day)** | **(g/day)** | **(g/kg)** | **(g)** | |  |
| **Castaneda (1995) [1]** | **-** | **-** | **-** | **-** | **0.92** | **60.7** | **58.0** | **3825** | **-** | **-** | **-** | **-** | **0.47** | **29.9** | **29.6** | **1881** | |  |
| **Rankin (2004) [2]*** | **0.21** | **16.4** | **0.21** | **16.4** | **0.09** | **7.0** | **6.3** | **491** | **0.21** | **16.4** | **0.21** | **16.4** | **0.09** | **7.0** | **6.3** | **491** | |  |
| **Candow (2006) (Post-RT) [3]*** | **0.30** | **25.6** | **0.30** | **25.6** | **0.13** | **11.0** | **10.8** | **921** | **0.30** | **25.6** | **0.30** | **25.6** | **0.13** | **11.0** | **10.8** | **921** | |  |
| **Candow (2006) (Pre-RT) [3]*** | **0.30** | **26.3** | **0.30** | **26.3** | **0.13** | **11.3** | **10.8** | **945** | **0.30** | **26.3** | **0.30** | **26.3** | **0.13** | **11.3** | **10.8** | **945** | |  |
| **Candow (2006) (Soy) [4]*** | **0.40** | **28.7** | **1.20** | **86.2** | **1.20** | **86.2** | **52.2** | **3619** | **0.40** | **28.7** | **1.20** | **86.2** | **1.20** | **86.2** | **52.2** | **3619** | |  |
| **Candow (2006) (Whey) [4]*** | **0.40** | **27.7** | **1.20** | **83.2** | **1.20** | **83.2** | **50.4** | **3493** | **0.40** | **27.7** | **1.20** | **83.2** | **1.20** | **83.2** | **50.4** | **3493** | |  |
| **Kerksick (2006) [5]*** | **0.57** | **48.0** | **0.57** | **48.0** | **0.57** | **48.0** | **40.0** | **3360** | **0.57** | **48.0** | **0.57** | **48.0** | **0.57** | **48.0** | **40.0** | **3360** | |  |
| **Cribb (2007) [6]*** | **0.43** | **30.3** | **1.30** | **91.0** | **1.30** | **91.0** | **100.1** | **7007** | **0.43** | **30.3** | **1.30** | **91.0** | **1.30** | **91.0** | **100.1** | **7007** | |  |
| **Hartman (2007) (Milk) [7]*** | **0.22** | **17.5** | **0.44** | **35.0** | **0.32** | **25.0** | **26.6** | **2100** | **0.22** | **17.5** | **0.44** | **35.0** | **0.32** | **25.0** | **26.6** | **2100** | |  |
| **Hartman (2007) (Soy) [7]*** | **0.21** | **17.5** | **0.42** | **35.0** | **0.30** | **25.0** | **25.2** | **2100** | **0.21** | **17.5** | **0.42** | **35.0** | **0.30** | **25.0** | **25.2** | **2100** | |  |
| **Hoffman (2007) [8]*** | **0.45** | **42.0** | **0.89** | **84.0** | **0.70** | **66.0** | **59.0** | **5544** | **0.43** | **40.0** | **0.85** | **80.0** | **0.67** | **62.9** | **56.3** | **5280** | |  |
| **Iglay (2007) [9]*** | **-** | **-** | **1.60** | **125.1** | **1.60** | **125.1** | **134.4** | **10510** | **-** | **-** | **0.80** | **65.3** | **0.80** | **65.3** | **67.2** | **5484** | |  |
| **Hoffman (2009) (AM/PM) [10]*** | **-** | **-** | **0.82** | **84.0** | **0.82** | **84.0** | **57.5** | **5880** | **-** | **-** | **0.82** | **84.0** | **0.82** | **84.0** | **57.5** | **5880** | |  |
| **Hoffman (2009) (Pre/Post-RT) [10]*** | **-** | **-** | **0.88** | **84.0** | **0.88** | **84.0** | **57.5** | **5880** | **-** | **-** | **0.88** | **84.0** | **0.88** | **84.0** | **57.5** | **5880** | |  |
| **Hulmi (2009) [11]*** | **0.20** | **15.0** | **0.39** | **30.0** | **0.11** | **8.6** | **16.6** | **1260** | **0.20** | **15.0** | **0.39** | **30.0** | **0.11** | **8.6** | **16.6** | **1260** | |  |
| **Shinkai (2009) (Fallers) [12]*** | **0.16** | **8.0** | **0.16** | **8.0** | **0.09** | **4.6** | **7.6** | **384** | **0.16** | **8.0** | **0.16** | **8.0** | **0.09** | **4.6** | **7.6** | **384** | |  |
| **Shinkai (2009) (Geriatric syndromes) [12]*** | **0.17** | **8.0** | **0.17** | **8.0** | **0.10** | **4.6** | **8.3** | **384** | **0.17** | **8.0** | **0.17** | **8.0** | **0.10** | **4.6** | **8.3** | **384** | |  |
| **Verdijk (2009) [13]*** | **0.13** | **10.0** | **0.25** | **20.0** | **0.11** | **8.6** | **9.1** | **720** | **0.13** | **10.0** | **0.25** | **20.0** | **0.11** | **8.6** | **9.1** | **720** | |  |
| **Bemben (2010) [14]*** | **0.40** | **35.0** | **0.40** | **35.0** | **0.17** | **15.0** | **14.3** | **1260** | **0.40** | **35.0** | **0.40** | **35.0** | **0.17** | **15.0** | **14.3** | **1260** | |  |
| **Josse (2010) [15]*** | **0.25** | **18.0** | **0.50** | **36.0** | **0.36** | **25.7** | **30.0** | **2160** | **0.25** | **18.0** | **0.50** | **36.0** | **0.36** | **25.7** | **30.0** | **2160** | |  |
| **Arazi (2011) [16]** | **0.60** | **43.8** | **1.80** | **131.4** | **1.80** | **131.4** | **100.8** | **7359** | **0.60** | **43.8** | **1.80** | **131.4** | **1.80** | **131.4** | **100.8** | **7359** | |  |
| **Deibert (2011) [17]** | **0.29** | **26.7** | **0.29** | **26.7** | **0.29** | **26.7** | **24.6** | **2243** | **0.29** | **26.7** | **0.29** | **26.7** | **0.29** | **26.7** | **24.6** | **2243** | |  |
| **Alemán-Mateo (2012) [18]** | **0.08** | **5.2** | **-** | **-** | **0.23** | **15.7** | **20.9** | **1434** | **0.08** | **5.2** | **-** | **-** | **0.23** | **15.7** | **20.9** | **1434** | |  |
| **Erskine (2012) [19]*** | **0.27** | **20.0** | **0.53** | **40.0** | **0.23** | **17.1** | **19.2** | **1436** | **0.27** | **20.0** | **0.53** | **40.0** | **0.23** | **17.1** | **19.2** | **1436** | |  |
| **Farnfield (2012) (Old) [20]*** | **0.32** | **26.6** | **0.32** | **26.6** | **0.14** | **11.4** | **11.6** | **958** | **0.32** | **26.6** | **0.32** | **26.6** | **0.14** | **11.4** | **11.6** | **958** | |  |
| **Farnfield (2012) (Young) [20]*** | **0.38** | **26.6** | **0.38** | **26.6** | **0.16** | **11.4** | **13.5** | **958** | **0.38** | **26.6** | **0.38** | **26.6** | **0.16** | **11.4** | **13.5** | **958** | |  |
| **Hida (2012) [21]*** | **0.25** | **15.0** | **0.25** | **15.0** | **0.25** | **15.0** | **13.9** | **840** | **0.24** | **14.7** | **0.24** | **14.7** | **0.24** | **14.7** | **13.6** | **823** | |  |
| **Tieland (2012) [22]*** | **0.20** | **15.0** | **-** | **-** | **0.41** | **30.0** | **68.2** | **5040** | **0.20** | **15.0** | **-** | **-** | **0.41** | **30.0** | **68.2** | **5040** | |  |
| **Tieland (2012) [23]*** | **0.19** | **15.0** | **0.38** | **30.0** | **0.38** | **30.0** | **63.4** | **5040** | **0.19** | **15.0** | **0.38** | **30.0** | **0.38** | **30.0** | **63.4** | **5040** | |  |
| **Weinheimer (2012) (20g) [24]*** | **0.11** | **10.0** | **0.23** | **20.0** | **0.23** | **20.0** | **57.4** | **5040** | **0.11** | **10.0** | **0.23** | **20.0** | **0.23** | **20.0** | **57.4** | **5040** | |  |
| **Weinheimer (2012) (40g) [24]*** | **0.24** | **20.0** | **0.47** | **40.0** | **0.47** | **40.0** | **119.0** | **10080** | **0.24** | **20.0** | **0.47** | **40.0** | **0.47** | **40.0** | **119.0** | **10080** | |  |
| **Weinheimer (2012) (60g) [24]*** | **0.34** | **30.0** | **0.67** | **60.0** | **0.67** | **60.0** | **169.1** | **15120** | **0.34** | **30.0** | **0.67** | **60.0** | **0.67** | **60.0** | **169.1** | **15120** | |  |
| **Weisgarber (2012) [25]*** | **-** | **-** | **0.30** | **26.5** | **0.17** | **15.1** | **9.6** | **848** | **-** | **-** | **0.30** | **26.5** | **0.17** | **15.1** | **9.6** | **848** | |  |
| **Arnarson (2013) [26]*** | **0.25** | **20.0** | **0.25** | **20.0** | **0.11** | **8.6** | **8.9** | **720** | **0.25** | **20.0** | **0.25** | **20.0** | **0.11** | **8.6** | **8.9** | **720** | |  |
| **Chalé (2013) [27]*** | **0.27** | **20.0** | **0.55** | **40.0** | **0.55** | **40.0** | **100.1** | **7308** | **0.27** | **20.0** | **0.55** | **40.0** | **0.55** | **40.0** | **100.1** | **7308** | |  |
| **Herda (2013) [28]*** | **0.26** | **20.0** | **0.78** | **60.0** | **0.37** | **28.6** | **20.9** | **1600** | **0.26** | **20.0** | **0.78** | **60.0** | **0.37** | **28.6** | **20.9** | **1600** | |  |
| **Leenders (2013) (Female) [29]*** | **0.24** | **15.0** | **0.24** | **15.0** | **0.24** | **15.0** | **39.8** | **2520** | **0.24** | **15.0** | **0.24** | **15.0** | **0.24** | **15.0** | **39.8** | **2520** | |  |
| **Leenders (2013) (Male) [29]*** | **0.18** | **15.0** | **0.18** | **15.0** | **0.18** | **15.0** | **30.0** | **2520** | **0.18** | **15.0** | **0.18** | **15.0** | **0.18** | **15.0** | **30.0** | **2520** | |  |
| **Volek (2013) (Soy) [30]*** | **0.34** | **24.5** | **0.34** | **24.5** | **0.34** | **24.5** | **93.3** | **6714** | **0.33** | **23.7** | **0.33** | **23.7** | **0.33** | **23.7** | **90.2** | **6495** | |  |
| **Volek (2013) (Whey) [30]*** | **0.30** | **22.5** | **0.30** | **22.5** | **0.30** | **22.5** | **83.2** | **6166** | **0.29** | **21.7** | **0.29** | **21.7** | **0.29** | **21.7** | **80.2** | **5947** | |  |
| **Alemán-Mateo (2014) [31]** | **0.09** | **6.0** | **-** | **-** | **0.26** | **18.1** | **21.5** | **1522** | **0.09** | **6.0** | **-** | **-** | **0.26** | **18.1** | **21.5** | **1522** | |  |
| **Babault (2014) (Casein) [32]** | **0.13** | **10.0** | **0.39** | **30.0** | **0.31** | **24.3** | **21.9** | **1700** | **0.13** | **10.0** | **0.39** | **30.0** | **0.31** | **24.3** | **21.9** | **1700** | |  |
| **Babault (2014) (Whey) [32]** | **0.12** | **9.8** | **0.25** | **19.5** | **0.10** | **7.7** | **8.2** | **644** | **0.12** | **9.8** | **0.25** | **19.5** | **0.10** | **7.7** | **8.2** | **644** | |  |
| **Farup (2014) [33]** | **0.29** | **30.0** | **0.29** | **30.0** | **0.29** | **30.0** | **8.2** | **840** | **0.29** | **30.0** | **0.29** | **30.0** | **0.29** | **30.0** | **8.2** | **840** | |  |
| **Figueroa (2014) (Casein) [34]** | **0.31** | **30.0** | **0.31** | **30.0** | **0.31** | **30.0** | **8.8** | **840** | **0.31** | **30.0** | **0.31** | **30.0** | **0.31** | **30.0** | **8.8** | **840** | |  |
| **Figueroa (2014) (Whey) [34]** | **0.12** | **10.0** | **0.12** | **10.0** | **0.12** | **10.0** | **13.6** | **1120** | **0.07** | **6.0** | **0.07** | **6.0** | **0.07** | **6.0** | **8.0** | **672** | |  |
| **Gryson (2014) [35]** | **0.12** | **9.8** | **0.25** | **19.5** | **0.10** | **7.7** | **8.2** | **644** | **0.12** | **9.8** | **0.25** | **19.5** | **0.10** | **7.7** | **8.2** | **644** | |  |
| **Mori (2014) [36]*** | **0.39** | **22.3** | **0.39** | **22.3** | **0.11** | **6.4** | **7.0** | **401** | **0.39** | **22.3** | **0.39** | **22.3** | **0.11** | **6.4** | **7.0** | **401** | |  |
| **Negro (2014) [37]*** | **0.29** | **20.0** | **0.29** | **20.0** | **0.13** | **8.6** | **7.1** | **480** | **0.29** | **20.0** | **0.29** | **20.0** | **0.13** | **8.6** | **7.1** | **480** | |  |
| **Babault (2015) (Pea) [38]** | **-** | **26.6** | **-** | **53.3** | **-** | **53.3** | **-** | **4476** | **-** | **25.0** | **-** | **49.9** | **-** | **49.9** | **-** | **4195** | |  |
| **Babault (2015) (Whey) [38]** | **-** | **25.7** | **-** | **51.3** | **-** | **51.3** | **-** | **4309** | **-** | **24.0** | **-** | **48.0** | **-** | **48.0** | **-** | **4029** | |  |
| **Hulmi (2015) [39]*** | **0.36** | **30.0** | **0.36** | **30.0** | **0.12** | **10.0** | **10.0** | **840** | **0.36** | **30.0** | **0.36** | **30.0** | **0.12** | **10.0** | **10.0** | **840** | |  |
| **Ormsbee (2015) (Casein) [40]*** | **0.30** | **30.0** | **0.30** | **30.0** | **0.30** | **30.0** | **8.3** | **840** | **0.30** | **30.0** | **0.30** | **30.0** | **0.30** | **30.0** | **8.3** | **840** | |  |
| **Ormsbee (2015) (Whey) [40]*** | **0.32** | **30.0** | **0.32** | **30.0** | **0.32** | **30.0** | **8.9** | **840** | **0.32** | **30.0** | **0.32** | **30.0** | **0.32** | **30.0** | **8.9** | **840** | |  |
| **Snijders (2015) [41]*** | **0.36** | **27.5** | **0.36** | **27.5** | **0.36** | **27.5** | **30.0** | **2310** | **0.36** | **27.5** | **0.36** | **27.5** | **0.36** | **27.5** | **30.0** | **2310** | |  |
| **Zhu (2015) [42]*** | **0.45** | **30.0** | **-** | **-** | **0.45** | **30.0** | **328.2** | **21924** | **0.42** | **27.9** | **-** | **-** | **0.42** | **27.9** | **305.2** | **20389** | |  |
| **Maltais (2016) [43]*** | **0.18** | **13.5** | **0.18** | **13.5** | **0.08** | **5.8** | **6.4** | **487** | **0.17** | **12.9** | **0.17** | **12.9** | **0.07** | **5.5** | **6.1** | **465** | |  |
| **Paoli (2016) [44]*** | **-** | **-** | **-** | **-** | **1.80** | **143.1** | **68.5** | **8014** | **-** | **-** | **-** | **-** | **0.95** | **75.5** | **36.1** | **4229** | |  |
| **Reidy (2016) (Blend) [45]*** | **0.28** | **21.9** | **0.28** | **21.9** | **0.28** | **21.9** | **23.6** | **1840** | **0.28** | **21.9** | **0.28** | **21.9** | **0.28** | **21.9** | **23.6** | **1840** | |  |
| **Reidy (2016) (Whey) [45]*** | **0.26** | **21.5** | **0.26** | **21.5** | **0.26** | **21.5** | **22.1** | **1806** | **0.26** | **21.5** | **0.26** | **21.5** | **0.26** | **21.5** | **22.1** | **1806** | |  |
| **Taylor (2016) [46]*** | **0.36** | **24.0** | **0.73** | **48.0** | **0.31** | **20.6** | **17.5** | **1152** | **0.36** | **24.0** | **0.73** | **48.0** | **0.31** | **20.6** | **17.5** | **1152** | |  |
| **Thomson (2016) (Dairy) [47]*** | **-** | **-** | **0.34** | **27.0** | **0.34** | **27.0** | **28.6** | **2268** | **-** | **-** | **0.34** | **27.0** | **0.34** | **27.0** | **28.6** | **2268** | |  |
| **Thomson (2016) (Soy) [47]*** | **-** | **-** | **0.34** | **27.0** | **0.34** | **27.0** | **28.7** | **2268** | **-** | **-** | **0.34** | **27.0** | **0.34** | **27.0** | **28.7** | **2268** | |  |
| **Hwang (2017) [48]*** | **0.31** | **25.0** | **0.31** | **25.0** | **0.20** | **16.4** | **14.3** | **1150** | **0.31** | **25.0** | **0.31** | **25.0** | **0.20** | **16.4** | **14.3** | **1150** | |  |
| **Niccoli (2017) [49]*** | **-** | **-** | **-** | **-** | **0.36** | **24.0** | **15.9** | **636** | **-** | **-** | **-** | **-** | **0.36** | **24.0** | **15.9** | **636** | |  |
| **Ottestad (2017) [50]*** | **0.26** | **20.0** | **-** | **-** | **0.52** | **40.0** | **43.4** | **3360** | **0.26** | **20.0** | **-** | **-** | **0.52** | **40.0** | **43.4** | **3360** | |  |
| **Rossato (2017) [51]*** | **-** | **-** | **-** | **-** | **1.20** | **81.1** | **120.8** | **5678** | **-** | **-** | **-** | **-** | **0.40** | **25.9** | **38.6** | **1814** | |  |
| **Vorup (2017) [52]*** | **0.25** | **18.0** | **0.50** | **36.0** | **0.14** | **10.3** | **11.9** | **864** | **0.21** | **15.0** | **0.41** | **30.0** | **0.12** | **8.6** | **10.0** | **720** | |  |
| **Bhasin (2018) [53]*** | **-** | **-** | **-** | **-** | **1.30** | **117.3** | **0.0** | **0** | **-** | **-** | **-** | **-** | **0.50** | **41.0** | **0.0** | **0** | |  |
| **Mitchell (2018) [54]*** | **0.23** | **20.0** | **0.23** | **20.0** | **0.23** | **20.0** | **9.7** | **840** | **0.23** | **20.0** | **0.23** | **20.0** | **0.23** | **20.0** | **9.7** | **840** | |  |
| **Nobuco (2018) (Post-RT) [55]*** | **0.41** | **27.1** | **0.42** | **27.4** | **0.18** | **11.7** | **15.1** | **986** | **0.41** | **26.8** | **0.41** | **26.8** | **0.18** | **11.5** | **14.8** | **965** | |  |
| **Nobuco (2018) (Pre-RT) [55]*** | **0.39** | **27.1** | **0.40** | **27.4** | **0.17** | **11.7** | **14.3** | **986** | **0.39** | **26.8** | **0.39** | **26.8** | **0.17** | **11.5** | **14.0** | **965** | |  |
| **Orsatti (2018) [56]*** | **0.37** | **25.0** | **0.37** | **25.0** | **0.37** | **25.0** | **40.9** | **2800** | **0.37** | **25.0** | **0.37** | **25.0** | **0.37** | **25.0** | **40.9** | **2800** | |  |
| **Park (2018) (1.2g/kg) [57]*** | **-** | **-** | **-** | **-** | **1.20** | **71.7** | **100.8** | **6021** | **-** | **-** | **-** | **-** | **0.40** | **24.7** | **33.6** | **2074** | |  |
| **Park (2018) (1.5g/kg) [57]*** | **-** | **-** | **-** | **-** | **1.50** | **84.4** | **126.0** | **7091** | **-** | **-** | **-** | **-** | **0.70** | **37.4** | **58.8** | **3145** | |  |
| **Sharp (2018) (Beef) [58]*** | **0.66** | **46.0** | **0.66** | **46.0** | **0.66** | **46.0** | **36.7** | **2576** | **0.66** | **46.0** | **0.66** | **46.0** | **0.66** | **46.0** | **36.7** | **2576** | |  |
| **Sharp (2018) (Chicken) [58]*** | **0.62** | **46.0** | **0.62** | **46.0** | **0.62** | **46.0** | **34.6** | **2576** | **0.62** | **46.0** | **0.62** | **46.0** | **0.62** | **46.0** | **34.6** | **2576** | |  |
| **Sharp (2018) (Whey) [58]*** | **0.62** | **46.0** | **0.62** | **46.0** | **0.62** | **46.0** | **34.5** | **2576** | **0.62** | **46.0** | **0.62** | **46.0** | **0.62** | **46.0** | **34.5** | **2576** | |  |
| **Sugihara (2018) [59]*** | **0.44** | **27.1** | **0.44** | **27.1** | **0.19** | **11.6** | **4.0** | **243** | **0.44** | **26.8** | **0.44** | **26.8** | **0.19** | **11.5** | **3.8** | **232** | |  |
| **Bartholomae (2019) [60]*** | **-** | **18.0** | **-** | **-** | **-** | **18.0** | **-** | **1008** | **-** | **14.0** | **-** | **-** | **0.21** | **14.0** | **-** | **784** | |  |
| **Centner (2019) [61]** | **0.18** | **15.0** | **0.18** | **15.0** | **0.18** | **15.0** | **9.9** | **840** | **0.18** | **15.0** | **0.18** | **15.0** | **0.18** | **15.0** | **9.9** | **840** | |  |
| **Drotningsvik (2019) [62]*** | **-** | **-** | **-** | **-** | **0.07** | **5.2** | **3.1** | **218** | **-** | **-** | **-** | **-** | **0.07** | **5.2** | **3.1** | **218** | |  |
| **Jonvik (2019) [63]*** | **0.36** | **28.7** | **0.72** | **57.4** | **0.51** | **41.0** | **43.2** | **3444** | **0.35** | **28.1** | **0.71** | **56.2** | **0.50** | **40.1** | **42.3** | **3372** | |  |
| **Nabuco (2018) [64]*** | **0.57** | **35.0** | **0.57** | **35.0** | **0.24** | **15.0** | **20.5** | **1260** | **0.57** | **35.0** | **0.57** | **35.0** | **0.24** | **15.0** | **20.5** | **1260** | |  |
| **Nahas (2019) [65]*** | **-** | **-** | **1.20** | **80.5** | **1.20** | **80.5** | **84.0** | **5636** | **-** | **-** | **0.40** | **26.5** | **0.40** | **26.5** | **28.0** | **1856** | |  |
| **ten Haaf (2019) [66]*** | **0.18** | **15.5** | **0.37** | **31.0** | **0.37** | **31.0** | **30.8** | **2604** | **0.18** | **15.0** | **0.35** | **29.9** | **0.35** | **29.9** | **29.7** | **2512** | |  |
| **Atherton (2020) [67]*** | **0.44** | **40.0** | **0.44** | **40.0** | **0.19** | **17.1** | **13.3** | **1200** | **0.23** | **20.0** | **0.23** | **20.0** | **0.10** | **8.6** | **7.0** | **600** | |  |
| **Björkman (2020) [68]** | **-** | **9.6** | **-** | **19.2** | **-** | **19.2** | **-** | **7016** | **-** | **8.9** | **-** | **17.7** | **-** | **17.7** | **-** | **6468** | |  |
| **Duarte (2020) [69]*** | **0.42** | **29.6** | **0.42** | **29.6** | **0.42** | **29.6** | **35.6** | **2485** | **0.41** | **28.8** | **0.41** | **28.8** | **0.41** | **28.8** | **34.8** | **2423** | |  |
| **Dulac (2020) (Casein) [70]*** | **0.13** | **10.1** | **0.40** | **30.3** | **0.40** | **30.3** | **33.4** | **2545** | **0.13** | **10.1** | **0.40** | **30.3** | **0.40** | **30.3** | **33.4** | **2545** | |  |
| **Dulac (2020) (Whey) [70]*** | **0.13** | **10.0** | **0.38** | **30.0** | **0.38** | **30.0** | **32.3** | **2520** | **0.13** | **10.0** | **0.38** | **30.0** | **0.38** | **30.0** | **32.3** | **2520** | |  |
| **Forbes (2020) (WPC Female) [71]*** | **2.00** | **130.2** | **2.00** | **130.2** | **1.71** | **111.6** | **72.0** | **4687** | **2.00** | **130.2** | **2.00** | **130.2** | **1.71** | **111.6** | **72.0** | **4687** | |  |
| **Forbes (2020) (WPC Male) [71]*** | **2.40** | **186.2** | **2.40** | **186.2** | **2.06** | **159.6** | **86.4** | **6705** | **2.40** | **186.2** | **2.40** | **186.2** | **2.06** | **159.6** | **86.4** | **6705** | |  |
| **Forbes (2020) (WPI Female) [71]*** | **2.00** | **130.8** | **2.00** | **130.8** | **1.71** | **112.1** | **72.0** | **4709** | **2.00** | **130.8** | **2.00** | **130.8** | **1.71** | **112.1** | **72.0** | **4709** | |  |
| **Forbes (2020) (WPI Male) [71]*** | **2.40** | **189.4** | **2.40** | **189.4** | **2.06** | **162.3** | **86.4** | **6817** | **2.40** | **189.4** | **2.40** | **189.4** | **2.06** | **162.3** | **86.4** | **6817** | |  |
| **Krull (2020) [72]*** | **-** | **-** | **0.26** | **21.0** | **0.26** | **21.0** | **43.3** | **3528** | **-** | **-** | **0.26** | **21.0** | **0.26** | **21.0** | **43.3** | **3528** | |  |
| **Kim**  **(2021)**  **(Evening)[73]*** | **0.19** | **10** | **-** | **-** | **0.19** | **10** | **16** | **840** | **0.19** | **10** | **-** | **-** | **0.19** | **10** | **16** | **840** | |  |
| **Kim**  **(2021)**  **(Morning)[73]*** | **0.18** | **10** | **-** | **-** | **0.18** | **10** | **15** | **840** | **0.18** | **10** | **-** | **-** | **0.18** | **10** | **15** | **840** | |  |
| **Griffen (2022) (with RT) [74]*** | **0.31** | **25** | **0.62** | **50** | **0.62** | **50** | **52** | **4200** | **0.31** | **25** | **0.62** | **50** | **0.62** | **50** | **52** | **4200** | |  |
| **Griffen (2022) (without RT) [74]*** | **0.32** | **25** | **-** | **-** | **0.64** | **50** | **54** | **4200** | **0.32** | **25** | **-** | **-** | **0.64** | **50** | **54** | **4200** | |  |
| **Sexton**  **(2021)**  **(Female)**  **[75]*** | **0.45** | **30** | **0.45** | **30** | **0.45** | **30** | **32** | **2107** | **0.45** | **30** | **0.45** | **30** | **0.45** | **30** | **32** | **2100** | |  |
| **Sexton**  **(2021)**  **(Male)**  **[75]*** | **0.40** | **30** | **0.40** | **30** | **0.40** | **30** | **28** | **2107** | **0.39** | **30** | **0.39** | **30** | **0.39** | **30** | **28** | **2100** | |  |
| **Ullevig**  **(2021)**  **[76]*** | **0.26** | **21** | **-** | **-** | **0.26** | **21** | **47** | **3762** | **0.26** | **20.6** | **-** | **-** | **0.26** | **21** | **47** | **3762** | |  |
| **McKenna**  **(2021)**  **[77]*** | **-** | **-** | **-** | **-** | **1.60** | **130** | **112** | **9106** | **-** | **-** | **-** | **-** | **0.60** | **49** | **42** | **3415** | |  |
| **Li(2021)**  **(Blend)**  **[78]*** | **0.31** | **17** | **-** | **-** | **0.31** | **17** | **57** | **3064** | **0.31** | **17** | **-** | **-** | **0.31** | **17** | **57** | **3064** | |  |
| **Li(2021)**  **(Soy)[78]*** | **0.33** | **18** | **-** | **-** | **0.33** | **18** | **60** | **3214** | **0.33** | **18** | **-** | **-** | **0.33** | **18** | **60** | **3214** | |  |
| **Li(2021)**  **(Whey)**  **[78]*** | **0.29** | **16** | **-** | **-** | **0.29** | **16** | **53** | **2915** | **0.29** | **16** | **-** | **-** | **0.29** | **16** | **53** | **2915** | |  |
| **Mertz**  **(2021)**  **(Collagen)[79]*** | **0.27** | **20** | **-** | **-** | **0.53** | **40** | **195** | **14610** | **0.27** | **20** | **-** | **-** | **0.53** | **40** | **195** | **14610** |  |  |
| **Mertz**  **(2021)**  **(Whey)**  **[79]*** | **0.27** | **20** | **-** | **-** | **0.53** | **40** | **195** | **14610** | **0.27** | **20** | **-** | **-** | **0.53** | **40** | **195** | **14610** |  |  |
| **Nygård**  **(2021)**  **[80]*** | **-** | **-** | **-** | **-** | **0.04** | **3** | **15** | **1096** | **-** | **-** | **-** | **-** | **0.04** | **3** | **15** | **1096** | |  |
| **Lamb**  **(2020)**  **[81]*** | **0.41** | **35** | **0.41** | **35** | **0.41** | **35** | **22** | **1897** | **0.41** | **35** | **0.41** | **35** | **0.41** | **35** | **22** | **1897** | |  |
| **Boutry-Regard**  **(2020)**  **[82]** | **0.40** | **20** | **-** | **-** | **0.40** | **20** | **34** | **1680** | **0.40** | **20** | **-** | **-** | **0.40** | **20** | **34** | **1680** | |  |

**All trials were used to create a forest plot to evaluate the effect of added protein intakes on muscle strength percentage changes compared with control groups; Studies marked with asterisk were also used to create spline models to evaluate the relationship between total protein intake and muscle strength percentage changes from baselines.**

*Supplementary Table S6* **Summary of conditions of the studies’ interventions**

| **Author  and Year** | **Dietary  intervention** | **Control** | **Meal or  Supplementation** | **Protein  source** | **Energy  balance** | **Duration  (weeks)** | **Type of**  **Exercise** | **(times/ week)** |
| --- | --- | --- | --- | --- | --- | --- | --- | --- |
| **Castaneda (1995) [1]** | **protein 0.92 g/kg/d** | **protein 0.45 g/kg /d** | **Meal** | **Milk** | **Intake = Expenditure** | **9** | **Physical activity as usual** | **0** |
| **Rankin (2004) [2]*** | **protein 0.09 g/kg/d (protein 0.21 g/kg, carbohydrate 0.92 g/kg, fat 0.06 g/kg) at training days (3 d/wk)** | **protein 0 g/d (carbohydrate 1.25 g/kg, electrolyte) at training days (3 d/wk)** | **Supplementation** | **Milk** | **No intervention** | **10** | **Resistance exercise** | **3** |
| **Candow (2006) (Post-RT) [3]*** | **protein 0.3 g/kg/d [immediately before resistance training,**  **3 d/wk] placebo (maltodextrin, sucrose) [immediately after resistance training,**  **3 d/wk] Myoplex^®^ (WPC, WPI, calcium caseinate, milk protein isolate, sodium caseinate, egg albumin)** | **protein 0 g/kg/d [immediately before resistance training,**  **3 d/wk] placebo [immediately after resistance training,**  **3 d/wk] placebo** | **Supplementation** | **Mixture** | **No intervention** | **12** | **Resistance exercise** | **3** |
| **Candow (2006) (Pre-RT) [3]*** | **protein 0.3 g/kg/d [immediately before resistance training,**  **3 d/wk] Myoplex [immediately after resistance training,**  **3 d/wk] placebo** | **protein 0 g/kg/d [immediately before resistance training,**  **3 d/wk] placebo [immediately after resistance training,**  **3 d/wk] placebo** | **Supplementation** | **Mixture** | **No intervention** | **12** | **Resistance exercise** | **3** |
| **Candow (2006) (Soy) [4]*** | **protein 1.2 g/kg/d**  **soy protein 1.2 g/kg, sucrose 0.3 g/kg, consumed in three equal doses (i.e., 0.5 g/kg supplement powder in water before their training session, after their training sesson, and before going to bed)** | **protein 0 g/kg/d**  **maltodextrin 1.2 g/kg, sucrose 0.3 g/kg, consumed in three equal doses** | **Supplementation** | **Soy** | **No intervention** | **6** | **Resistance exercise** | **5.3** |
| **Candow (2006) (Whey) [4]*** | **protein 1.2 g/kg/d**  **whey protein 1.2 g/kg, sucrose 0.3 g/kg, consumed in three equal doses** | **protein 0 g/kg/d**  **maltodextrin 1.2 g/kg, sucrose 0.3 g/kg, consumed in three equal doses** | **Supplementation** | **Whey** | **No intervention** | **6** | **Resistance exercise** | **5.3** |
| **Kerksick (2006) [5]*** | **protein 48 g/d**  **whey 40 g, casein 8 g** | **protein 0 g/d carbohydrate 48 g** | **Supplementation** | **Mixture** | **No intervention** | **10** | **Resistance exercise** | **4** |
| **Cribb (2007) [6]*** | **protein 1.3 g/kg/d whey protein 1.3 g/kg, carbohydrate 0.08 g/kg, fat 0.02 g/kg** | **protein 0 g/kg/d**  **carbohydrate 1.3 g/kg** | **Supplementation** | **Whey** | **No intervention** | **11** | **Resistance exercise** | **3** |
| **Hartman (2007) (Milk) [7]*** | **protein 25 g/d (milk protein 17.5 g, carbohydrate 25.7 g, fat 0.4 g) ×2 times at training days (5 d/wk)** | **protein 0 g/d maltodextrin** | **Supplementation** | **Milk** | **No intervention** | **12** | **Resistance exercise** | **5** |
| **Hartman (2007) (Soy) [7]*** | **protein 25 g/d (soy protein 17.5 g, carbohydrate 25.7 g, fat 0.4 g, isoflavone free) ×2 times at training days (5 d/wk)** | **protein 0 g/d maltodextrin** | **Supplementation** | **Soy** | **No intervention** | **12** | **Resistance exercise** | **5** |
| **Hoffman (2007) [8]*** | **protein 66 g/d Metamyosyn® (protein 42 g, carbohydrate 18 g,**  **fat 3 g) × 2times at training days (4 d/wk) ×1time at non training day ※blend of milk protein concentrate, WPC, L-glutamine, dried egg white** | **protein 3 g/d placebo**  **(protein 2 g, carbohydrate 63 g,**  **fat 2 g) × 2times at training days (4 d/wk) ×1time at non training day** | **Supplementation** | **Mixture** | **No intervention** | **12** | **Resistance exercise** | **4** |
| **Iglay (2007) [9]*** | **protein 1.6 g/kg/d**  **more animal-based food (eggs, meats, daily)**  **egg:striated tissue (beef, poultry pork, fish): dairy = 25:20:15 % of total protein intake** | **protein 0.8 g/kg/d**  **egg:striated tissue (beef, poultry pork, fish):dairy = 5:25:15 % of total protein intake** | **Meal** | **Mixture** | **Intake = Expenditure** | **12** | **Resistance exercise** | **3** |
| **Hoffman (2009) (AM/PM) [10]*** | **protein 84 g/d (proprietary blend of protein 42 g (enzymatically hydrolyzed collagen protein isolate, WPI, casein protein isolate), carbohydrate 2 g)**  **× 2times/d** (**on awakening and in the evening)** | **protein 0 g/d**  **did not use any protein or other  nutritional supplement** | **Supplementation** | **Mixture** | **No intervention** | **10** | **Resistance exercise** | **4** |
| **Hoffman (2009) (Pre/Post-RT) [10]*** | **protein 84 g/d (proprietary blend of protein 42 g (enzymatically hydrolyzed collagen protein isolate, WPI, casein protein isolate), carbohydrate 2 g)**  **× 2times/d** (**immediately before and after workout at training days, on awakening and in the evening at non training days)** | **protein 0 g/d**  **did not use any protein or other  nutritional supplement** | **Supplementation** | **Mixture** | **No intervention** | **10** | **Resistance exercise** | **4** |
| **Hulmi (2009) [11]*** | **protein 8.6 g/d**  **whey protein 15 g**  **× 2times at training days (2 d/wk)** | **protein 0 g/d**  **non-energetic placebo** | **Supplementation** | **Whey** | **No intervention** | **21** | **Resistance exercise** | **2** |
| **Shinkai (2009) (Fallers) [12]*** | **protein 4.57 g/d**  **soy peptide 8 g, 4 d/wk** | **protein 0 g/d**  **abandon the use of a placebo** | **Supplementation** | **Soy** | **No intervention** | **12** | **Other exercise** | **2** |
| **Shinkai (2009) (Geriatric syndromes) [12]*** | **protein 4.57 g/d**  **soy peptide 8 g, 4 d/wk** | **protein 0 g/d**  **abandon the use of a placebo** | **Supplementation** | **Soy** | **No intervention** | **12** | **Other exercise** | **2** |
| **Verdijk (2009) [13]*** | **protein 8.6 g/d**  **casein hydrolysate 10 g ×2 times at training days (3 d/wk)** | **protein 0 g/d water only** | **Supplementation** | **Casein** | **No intervention** | **12** | **Resistance exercise** | **3** |
| **Bemben (2010) [14]*** | **protein 15 g/d**  **(whey protein 35 g,**  **sports drink), at training days (3 d/wk)** | **protein 0 g/d**  **sports drink, at training days (3 d/wk)** | **Supplementation** | **Whey** | **No intervention** | **12** | **Resistance exercise** | **3** |
| **Josse (2010) [15]*** | **protein 25.7 g/d fat free milk protein drink (protein 18 g) ×2 times at training days (5 d/wk)** | **protein 0 g/d maltodextrin** | **Supplementation** | **Milk** | **No intervention** | **12** | **Resistance exercise** | **5** |
| **Arazi (2011) [16]** | **protein 1.8 g/kg/d**  **whey protein** | **protein 0 g/kg/d**  **starch** | **Supplementation** | **Whey** | **No intervention** | **8** | **Resistance exercise** | **3** |
| **Deibert (2011) [17]** | **protein 26.7 g/d  commercially available soy–yogurt–honey 50 g (protein 26.7 g)** | **protein 0 g/d**  **abandon the use of a placebo** | **Supplementation** | **Soy** | **No intervention** | **12** | **Resistance exercise** | **2** |
| **Alemán-Mateo (2012) [18]** | **protein 15.7 g/d**  **ricotta cheese 210 g (protein15.7 g, fat 18.4 g, carbohydrates 10.4 g)** | **protein 0 g/d**  **abandon the use of a placebo** | **Supplementation** | **Whey** | **No intervention** | **13** | **No instructions** | **0** |
| **Erskine (2012) [19]*** | **protein 17.1 g /d (whey protein 20 g,**  **lactose 6.7 g) ×2 times at training days (3 d/wk)** | **protein 0 g/d**  **lactose 6.8 g ×2 times at training days (3 d/wk)** | **Supplementation** | **Whey** | **Intake = Expenditure** | **12** | **Resistance exercise** | **3** |
| **Farnfield (2012) (Old)**  **[20]*** | **protein 11.4 g/d**  **whey protein powder (beta-lactogloblin enriched WPI, total of amino acids 26.6 g) , at training days (3 d/wk)** | **protein 0 g/d**  **placebo (aspartame), at training days (3 d/wk)** | **Supplementation** | **Whey** | **No intervention** | **12** | **Resistance exercise** | **3** |
| **Farnfield (2012) (Young)**  **[20]*** | **protein 11.4 g/d**  **whey protein powder (beta-lactogloblin enriched WPI, total of amino acids 26.6 g) , at training days (3 d/wk)** | **protein 0 g/d**  **placebo (aspartame), at training days (3 d/wk)** | **Supplementation** | **Whey** | **No intervention** | **12** | **Resistance exercise** | **3** |
| **Hida (2012) [21]*** | **protein 15 g/d**  **egg white protein 15 g, carbohydrate 2.0 g,**  **fat 0.8 g** | **protein 0.3 g/d**  **maltodextrin 17.5 g,**  **fat 0.8 g** | **Supplementation** | **Egg** | **No intervention** | **8** | **No instructions** | **0** |
| **Tieland (2012) [22]*** | **protein 30 g /d (milk protein concentrate 15 g, lactose 7.1 g, fat**  **0.5 g) ×2 times/d** | **protein 0 g /d (lactose 7.1 g, fat 0.5 g) ×2 times/d** | **Supplementation** | **Milk** | **No intervention** | **24** | **No instructions** | **0** |
| **Tieland (2012) [23]*** | **protein 30 g /d (milk protein concentrate 15 g, lactose 7.1 g, fat 0.5 g) ×2 times/d** | **protein 0 g /d (lactose 7.1 g, fat 0.5 g) ×2 times/d** | **Supplementation** | **Milk** | **No intervention** | **24** | **Resistance exercise** | **2** |
| **Weinheimer (2012) (20g) [24]*** | **protein 20 g/d WPC, maltodextrin** | **protein 0 g/d**  **maltodextrin** | **Supplementation** | **Whey** | **No intervention** | **36** | **Resistance exercise & other exercise** | **3** |
| **Weinheimer (2012) (40g) [24]*** | **protein 40 g/d  WPC, maltodextrin** | **protein 0 g/d**  **maltodextrin** | **Supplementation** | **Whey** | **No intervention** | **36** | **Resistance exercise & other exercise** | **3** |
| **Weinheimer (2012) (60g) [24]*** | **protein 60 g/d  WPC, maltodextrin** | **protein 0 g/d**  **maltodextrin** | **Supplementation** | **Whey** | **No intervention** | **36** | **Resistance exercise & other exercise** | **3** |
| **Weisgarber (2012) [25]*** | **protein 0.17 g/kg/d WPI 0.3 g/kg**  **at training days (4 d/wk)** | **protein 0 g/kg/d**  **cornstarch  maltodextrin, sucrose at training days (4 d/wk)** | **Supplementation** | **Whey** | **No intervention** | **8** | **Resistance exercise** | **4** |
| **Arnarson (2013) [26]*** | **protein 8.6 g/d protein (sweet whey concentrate) 20 g, carbohydrates 20 g, fat**  **1 g, at training days**  **(3 d/wk)** | **protein 0 g/d carbohydrates 40 g, fat**  **1 g, at training days**  **(3 d/wk)** | **Supplementation** | **Whey** | **No intervention** | **12** | **Resistance exercise** | **3** |
| **Chalé (2013) [27]*** | **protein 40 g/d**  **(WPC 20 g, maltodextrin 25 g, fat 1 g) ×2 times/d** | **protein 0 g/d**  **(maltodextrin 45 g, fat**  **1 g) ×2 times/d** | **Supplementation** | **Whey** | **No intervention** | **26** | **Resistance exercise** | **3** |
| **Herda (2013) [28]*** | **protein 28.6 g/d WPC 20 g**  **× 2 times at training days (3 d/wk) ×1 time at non training day (4 d/wk)** | **protein 0 g/d maltodextrin 27 g**  **× 2 times at training days (3 d/wk) ×1 time at non training day (4 d/wk)** | **Supplementation** | **Whey** | **No intervention** | **8** | **Resistance exercise** | **3** |
| **Leenders (2013) (Female) [29]*** | **protein 15 g/d　 milk protein concentrate 15 g (80%casein 20% whey protein), fat 0.5 g, lactose 7.13 g** | **protein 0 g/d lactose 7.13 g** | **Supplementation** | **Milk** | **No intervention** | **24** | **Resistance exercise** | **3** |
| **Leenders (2013) (Male) [29]*** | **protein 15 g/d　 milk protein concentrate 15 g (80%casein 20% whey protein), fat 0.5 g, lactose 7.13 g** | **protein 0 g/d lactose 7.13 g** | **Supplementation** | **Milk** | **No intervention** | **24** | **Resistance exercise** | **3** |
| **Volek (2013) (Soy) [30]*** | **protein ~22 g/d soy isolate (isoﬂavone free)** | **protein 0 g/d**  **maltodextrin** | **Supplementation** | **Soy** | **No intervention** | **39** | **Resistance exercise** | **2.7** |
| **Volek (2013) (Whey) [30]*** | **protein ~22 g/d whey protein** | **protein 0 g/d**  **maltodextrin** | **Supplementation** | **Whey** | **No intervention** | **39** | **Resistance exercise** | **2.7** |
| **Alemán-Mateo (2014) [31]** | **protein 18.1 g/d**  **ricotta cheese 210 g (protein 18.1 g)** | **protein 0 g/d**  **abandon the use of a placebo** | **Supplementation** | **Whey** | **No intervention** | **12** | **No instructions** | **0** |
| **Babault (2014) (Casein) [32]** | **protein 24.3 g/d**  **(casein protein 10 g, sucrose 10.5 g, maltodextrine 7.5 g, lactose 1 g, soy lecithin**  **1 g), ×3 times at training day (3 d/wk), ×2 times at non taining day (4d/wk)** | **protein 0 g/d**  **(sucrose 10.5 g, maltodextrine 19.5 g),**  **×3 times at training day (3 d/wk), ×2 times at non taining day (4d/wk)** | **Supplementation** | **Casein** | **No intervention** | **10** | **Resistance exercise** | **3** |
| **Babault (2014) (Whey) [32]** | **protein 24.3 g/d**  **(native whey protein 10 g, sucrose 10.5 g, maltodextrine 7.5 g, lactose 1 g, soy lecithin**  **1 g), ×3 times at training day (3 d/wk), ×2 times at non taining day (4d/wk)** | **protein 0 g/d**  **(sucrose 10.5 g, maltodextrine 19.5 g),**  **×3 times at training day (3 d/wk), ×2 times at non taining day (4d/wk)** | **Supplementation** | **Whey** | **No intervention** | **10** | **Resistance exercise** | **3** |
| **Farup (2014) [33]** | **protein 7.7 g/d**  **(whey protein hydrolysate 19.5 g, glucose 19.5 g), at training day (33 d/84 d)** | **protein 0 g/d**  **glucose 39 g, at training day (33 d/84 d)** | **Supplementation** | **Whey** | **No intervention** | **12** | **Resistance exercise** | **2.8** |
| **Figueroa (2014) (Casein)[34]** | **protein 30 g/d**  **casein protein 30 g, carbohydrate 3 g,**  **fat 0.5 g** | **protein 0 g/d**  **maltodextrin 34 g,**  **fat 2 g** | **Supplementation** | **Casein** | **No intervention** | **4** | **Resistance exercise & other exercise** | **3** |
| **Figueroa (2014) (Whey)**  **[34]** | **protein 30 g/d**  **whey protein 30 g, carbohydrate 4 g,**  **fat 1.5 g** | **protein 0 g/d**  **maltodextrin 34 g,**  **fat 2 g** | **Supplementation** | **Whey** | **No intervention** | **4** | **Resistance exercise & other exercise** | **3** |
| **Gryson (2014) [35]** | **protein 10 g /d**  **milk protein (casein 80 % , whey20 % ) 10 g** | **protein 4 g/d**  **milk protein(casein 80 % , whey20 %) 4 g** | **Supplementation** | **Milk** | **No intervention** | **16** | **Resistance exercise & other exercise** | **3** |
| **Mori (2014) [36]*** | **protein 6.4 g/d**  **ｗhey protein 22.3g, at training day (2d/wk)** | **protein 0 g/d**  **non-caloric soft drink** | **Supplementation** | **Whey** | **No intervention** | **9** | **Resistance exercise & other exercise** | **2** |
| **Negro (2014) [37]*** | **protein 8.6 g/d lean beef (tinned meat) 135 g (protein 20 g, fat 1.7 g), at training day (3d/wk)** | **protein 0 g/d**  **abandon the use of a placebo** | **Supplementation** | **Meat** | **No intervention** | **8** | **Resistance exercise** | **3** |
| **Babaul (2015) (Pea) [38]** | **protein 53.3 g/d**  **vegetable pea protein isolate 25 g, fat-reduced cocoa (protein 1.6 g),**  **2 times/d** | **protein 3.3 g/d**  **maltodextrin, fat-reduced cocoa (protein 1.7 g), 2 times/d** | **Supplementation** | **Pea** | **No intervention** | **12** | **Resistance exercise** | **3** |
| **Babaul (2015) (Whey) [38]** | **protein 51.3 g/d**  **WPC 23.9 g, fat-reduced cocoa (protein 1.7 g),**  **2 times/d** | **protein 3.3 g/d**  **maltodextrin, fat-reduced cocoa (protein 1.7 g), 2 times/d** | **Supplementation** | **Whey** | **No intervention** | **12** | **Resistance exercise** | **3** |
| **Hulmi (2015) [39]*** | **protein 10 g/d**  **whey concentrate**  **(whey protein 30 g, lactose 5 g, fat < 1 g ),**  **at training days**  **(28 d/12 wk)** | **protein 0 g/d**  **maltodextrin 34.5 g,**  **at training days**  **(28 d/12 wk)** | **Supplementation** | **Whey** | **No intervention** | **12** | **Resistance exercise** | **2.3** |
| **Ormsbee (2015) (Casein) [40]*** | **protein 30 g/d micellar casein protein 30 g, carbohydrate 3 g,**  **fat 0.5 g** | **protein 0 g/d**  **maltodextrin 34 g, fat 2 g** | **Supplementation** | **Casein** | **No intervention** | **4** | **Resistance exercise & other exercise** | **3** |
| **Ormsbee (2015) (Whey) [40]*** | **protein 30 g/d whey protein (50% WPI, 50% WPC) 30 g, carbohydrate 4 g,**  **fat 1.5 g** | **protein 0 g/d**  **maltodextrin 34 g,**  **fat 2 g** | **Supplementation** | **Whey** | **No intervention** | **4** | **Resistance exercise & other exercise** | **3** |
| **Snijders (2015) [41]*** | **protein 27.5 g/d casein hydrolysate 13.75 g, casein 13.75 g, carbohydrate 15 g,**  **fat 0.1 g** | **protein 0 g/d noncaloric placebo beverage** | **Supplementation** | **Casein** | **No intervention** | **12** | **Resistance exercise** | **3** |
| **Zhu (2015) [42]*** | **protein 30 g/d**  **protein (skim milk + WPI) 30.1 g, carbohydrate 13.2 g,**  **fat 2.3 g** | **protein 2.1 g/d**  **protein 2.1 g, carbohydrate 42.3 g,**  **fat 2.0 g** | **Supplementation** | **Whey** | **No intervention** | **104** | **No instructions** | **0** |
| **Maltais (2016) [43]*** | **protein 5.8 g/d chocolate cow milk + milk powder (protein 13.5 g, carbohydrate 37.5 g, fat 3.8 g), at training days (3 d/wk)** | **protein 0.3 g/d**  **rice milk (protein 0.6 g, carbohydrate 59.5 g, fat 3.8 g) , at training days (3 d/wk)** | **Supplementation** | **Milk** | **No intervention** | **12** | **Resistance exercise** | **3** |
| **Paoli (2016) [44]*** | **protein 1.8 g/kg/d**  **whey protein diet (protein 28%, fats 25%, carbohydrates 47%) + whey protein 15-20 g, ×2 times at training day**  **(3 d/wk)** | **protein 0.85 g/kg/d**  **diet (protein 13%, fats 25%, carbohydrates 62%, the surplus of calories given by protein supplements was subsitituted by carbohydrates) + water with non-caloric sweetener, ×2 times at training day (3 d/wk)** | **Meal + Supplementation** | **Whey** | **No intervention** | **8** | **Resistance exercise** | **3** |
| **Reidy (2016) (Blend) [45]*** | **protein 22 g/d**  **soy-dairy protein blend (soy protein isolate 25%, WPI 25%, sodium caseinate 50%)** | **0 g protein/d**  **maltodextrin** | **Supplementation** | **Mixture** | **No intervention** | **12** | **Resistance exercise** | **3** |
| **Reidy (2016) (Whey) [45]*** | **protein 22 g/d  WPI** | **0 g protein/d maltodextrin** | **Supplementation** | **Whey** | **No intervention** | **12** | **Resistance exercise** | **3** |
| **Taylor (2016) [46]*** | **protein 20.6 g/d**  **whey protein 24 g, ×2 times at training days (3 d/wk)** | **protein 0 g/d**  **maltodextrin 24 g, ×2 times at training days (3 d/wk)** | **Supplementation** | **Whey** | **No intervention** | **8** | **Resistance exercise & other exercise** | **4** |
| **Thomson (2016) (Dairy) [47]*** | **protein > 1.2 g/kg/d**  **daily protein ~27 g/d**  **(reduced fat milk 475 g, no fat yoghurt 200g, syrup 20 ml)** | **protein < 1.2 g/kg/d**  **isocaloric carbohydrate foods (orange juice 500 ml, poly-joule 13 g,**  **2 biscuits)** | **Supplementation** | **Milk** | **Intake = Expenditure** | **12** | **Resistance exercise** | **3** |
| **Thomson (2016) (Soy) [47]*** | **protein > 1.2 g/kg/d**  **soy protein ~27 g/d**  **(soy milk 300 g, soy yoghurt 100 g, protein powder 20 g,**  **poly-joule 15 g)** | **protein < 1.2 g/kg/d**  **isocaloric carbohydrate foods (orange juice 500 ml, poly-joule 13 g,**  **2 biscuits)** | **Supplementation** | **Soy** | **Intake = Expenditure** | **12** | **Resistance exercise** | **3** |
| **Hwang (2017) [48]*** | **protein 16.4 g/d whey protein　 [RT・ReT] 14.3 g/d （25 g/d at training day (4d/wk), ×8wk [DT] 25 g/d, ×2wk** | **protein 0 g/d maltodextrin　 [RT・ReT] 14.3 g/d （25 g/d at training day (4d/wk), ×8wk [DT] 25 g/d, ×2wk** | **Supplementation** | **Whey** | **No intervention** | **10** | **Resistance exercise** | **3.2** |
| **Niccoli (2017) [49]*** | **protein 24 g/d**  **whey protein**  **9 g at breakfast (hot cereal), 7.5 g at lunch and dinner (milk products)** | **protein 0 g/d**  **at breakfast (hot cereal), at lunch and dinner (milk products) without whey protein** | **Meal + Supplementation** | **Whey** | **No intervention** | **4** | **Other exercise** | **-** |
| **Ottestad (2017) [50]*** | **protein 40 g/d**  **protein-enrich milk**  **(protein 5.1% (casein ~80%, whey ~20%), carbohydrate 4.9%, fat <0.1%), 2×400 ml/d** | **protein 0 g/d**  **isocaloric, non-nitrogenous carbohydrate drink**  **(sugar, xantan gum, maltodextrin, calcium), 2×400 ml/d** | **Supplementation** | **Milk** | **No intervention** | **12** | **No instructions** | **0** |
| **Rossato (2017) [51]*** | **protein ~1.2 g/kg/d high quality protein, (meat, fish, eggs, milk, other dairy products) were reccommended.**  **carbohydrate, lipids were 50%, 15% of total caloric value** | **protein ~0.8 g/kg/d carbohydrate, lipids were 50%, 25-30% of total caloric value lipids were 25-30% of total calories** | **Meal** | **Mixture** | **Intake = Expenditure** | **10** | **Resistance exercise** | **3** |
| **Vorup (2017) [52]*** | **protin 10.3 g/d**  **low fat milk + protein (protein 18 g, carbohydrate 28.6 g, fat 1.6, vitamin B_2,_ B_6_, B_12_/250 ml) ×2 times at training days (2 d/wk)** | **protin 1.7 g/d**  **apple or orange juice (protein 3 g, carbohydrate 47.5 g, fat 0.5 g/500 ml), ×2 times at training days (2 d/wk)** | **Supplementation** | **Milk** | **No intervention** | **12** | **Other exercise** | **2** |
| **Bhasin (2018) [53]*** | **protein 1.3 g/kg/d**  **packaged meals (protein 0.7 g/kg/d), discretionary foods (protein 0.1 g/kg/d), supplement (casein and whey protein, 0.5 g/kg/d protein)** | **protein 0.8 g/kg/d**  **packaged meals (protein 0.7 g/kg/d), discretionary foods (protein 0.1 g/kg/d), supplement (protein 0 g/kg/d )** | **Meal + Supplementation** | **Milk** | **No intervention** | **26** | **Physical activity as usual** | **0** |
| **Mitchell (2018) [54]*** | **protein 20 g/d**  **milk protein concentrate (casein 80%, whey protein 20%)** | **protein 0 g/d**  **maltodextrin** | **Supplementation** | **Milk** | **No intervention** | **6** | **Resistance exercise** | **1** |
| **Nobuco (2018) (Post-RT) [55]*** | **protein 11.7 g/d**  **[Pre] protein 0.3 g, carboohydrates 33.3 g　[Post] hydrolyzed whey protein 35 g (protein 27.1 g, carbohydrates 5.2 g, fat 0.2 g ), at training days (3 d/wk)** | **protein 0.26 g/d**  **[Pre] protein 0.3 g, carboohydrates 33.3 g [Post] protein 0.3 g, carboohydrates 33.3 g, at training days (3 d/wk)** | **Supplementation** | **Whey** | **No intervention** | **12** | **Resistance exercise** | **3** |
| **Nobuco (2018) (Pre-RT) [55]*** | **protein 11.7 g/d**  **[Pre-RT] hydrolyzed whey protein 35 g (protein 27.1 g , carbohydrates 5.2 g, fat 0.2 g )**  **[Post-RT] protein 0.3 g, carboohydrates 33.3 g, at training days (3 d/wk)** | **protein 0.26 g/d**  **[Pre-RT] protein 0.3 g, carboohydrates 33.3 g [Post-RT] protein 0.3 g, carboohydrates 33.3 g, at training days (3 d/wk)** | **Supplementation** | **Whey** | **No intervention** | **12** | **Resistance exercise** | **3** |
| **Orsatti (2018) [56]*** | **protein 25 g/d**  **soy protein** | **protein 0 g/d**  **maltodextrin** | **Supplementation** | **Soy** | **No intervention** | **16** | **Resistance exercise** | **2.2** |
| **Park (2018) (1.2g/kg) [57]*** | **protein 1.2 g/kg/d**  **combination 5 × 10 g packs: protein powders (fat 0.5 g, cocoa powder 0.2 g, whey protein 9.3 g /10 g pack) or placebo (fat 0.5 g, cocoa powder 0.2 g, maltodextrin 9.3 g/10 g pack) + protein powder** | **protein 0.8 g/kg/d**  **5 × 10 g packs placebo (fat 0.5 g, cocoa powder 0.2 g, maltodextrin 9.3 g/10 g pack)** | **Supplementation** | **Whey** | **No intervention** | **12** | **Physical activity as usual** | **0** |
| **Park (2018) (1.5g/kg) [57]*** | **protein 1.5 g/kg/d**  **combination 5 × 10 g packs: protein powders (fat 0.5 g, cocoa powder 0.2 g, whey protein 9.3 g/10 g pack) or placebo (fat 0.5 g, cocoa powder 0.2 g, maltodextrin 9.3 g/10 g pack) + protein powder** | **protein 0.8 g/kg/d**  **5 × 10 g packs placebo (fat 0.5 g, cocoa powder 0.2 g, maltodextrin 9.3 g/10 g pack)** | **Supplementation** | **Whey** | **No intervention** | **12** | **Physical activity as usual** | **0** |
| **Sharp (2018) (Beef) [58]*** | **protein 46 g/d**  **isolated beef protein** | **protein 0 g/d**  **maltodextrin** | **Supplementation** | **Meat** | **Intake = Expenditure** | **8** | **Resistance exercise & other exercise** | **5** |
| **Sharp (2018) (Chicken) [58]*** | **protein 46 g/d**  **hydrolysed chicken protein** | **protein 0 g/d**  **maltodextrin** | **Supplementation** | **Meat** | **Intake = Expenditure** | **8** | **Resistance exercise & other exercise** | **5** |
| **Sharp (2018) (Whey) [58]*** | **protein 46 g/d**  **WPC** | **protein 0 g/d**  **maltodextrin** | **Supplementation** | **Whey** | **Intake = Expenditure** | **8** | **Resistance exercise & other exercise** | **5** |
| **Sugihara (2018) [59]*** | **protein 15 g/d**  **hydrolyzed whey protein 35 g, at training days**  **(3 d/wk)** | **protein 0 g/d**  **maltodextrin 35 g, at training days (3 d/wk)** | **Supplementation** | **Whey** | **No intervention** | **12** | **Resistance exercise** | **3** |
| **Bartholomae (2019) [60]*** | **protein 18 g/d**  **egg-replacement patty manufactured from**  **mung bean** | **protein 4 g/d**  **control biscuit** | **Supplementation** | **Pea** | **No intervention** | **8** | **Physical activity as usual** | **0** |
| **Centner (2019) [61]** | **protein 15 g/d**  **collagen hydrolysate15 g** | **protein 0 g/d**  **silicon dioxide** | **Supplementation** | **Collagen** | **No intervention** | **8** | **Resistance exercise** | **3** |
| **Drotningsvik (2019) [62]*** | **protein 5.2 g/d**  **fish (blue whiting) protein hydrolysate 5.2 g** | **protein 0 g/d**  **non-caloric soft drink** | **Supplementation** | **Fish** | **No intervention** | **6** | **No instructions** | **0** |
| **Jonvik (2019) [63]*** | **protein 41 g/d**  **(casein 28.7 g, fat 0.3 g, carbohydrate 2.6 g), ×2 times at training days**  **(3 d/wk), ×1 time at non training day** | **protein 0.9 g/d**  **(protein 0.6 g, fat 2.4 g, carbohydrate 25.8 g),**  **×2 times at training days**  **(3 d/wk), ×1 time at non training day** | **Supplementation** | **Casein** | **No intervention** | **12** | **Other exercise** | **3** |
| **Nabuco (2018) [64]*** | **protein 15 g/d**  **hydrolyzed whey protein 35 g, at training days**  **(3 d/wk)** | **protein 0 g/d**  **maltodextrin** | **Supplementation** | **Whey** | **No intervention** | **12** | **Resistance exercise** | **3** |
| **Nahas (2019) [65]*** | **protein 1.2 g/kg/d**  **dietary plan** | **protein 0.8 g/kg/d**  **dietary plan** | **Meal** | **Mixture** | **No intervention** | **10** | **Resistance exercise** | **3** |
| **ten Haaf (2019) [66]*** | **protein 31 g/d**  **milk protein concentrate 36.8 g (protein 31 g), fat 1.1 g, lactose 14.5 g** | **protein 1.1 g/d**  **protein 1.1 g, fat 5.2 g, carbohydrates 36 g** | **Supplementation** | **Milk** | **No intervention** | **12** | **No instructions** | **0** |
| **Atherton (2020) [67]*** | **protein 17.1 g/d**  **(whey protein 40 g, carbohydrate 3.9 g, fat 2.8 g), at training days**  **(3 d/wk)** | **protin 8.6 g/d**  **(whey protein 20 g, carbohydrate 2.0 g, fat 1.4 g), at training days**  **(3 d/wk)** | **Supplementation** | **Whey** | **No intervention** | **10** | **Resistance exercise** | **3** |
| **Björkman (2020) [68]** | **protein 19.2 g/d**  **extra milk derived proteins (whey 48%) 20 g, ×2 tiems/d** | **protein 1.5 g/d**  **isocaloric placebo (whey 20%) 3.75 g, ×2 tiems/d** | **Supplementation** | **Whey** | **No intervention** | **52** | **Exercise recommended** | **0** |
| **Duarte (2020) [69]*** | **protein 29.6 g/d**  **whey (protein 87%) 34 g** | **protein 0.7 g/d**  **mixture (cocoa 30%, amylopectin70%, protein 2.1%) 35 g** | **Supplementation** | **Whey** | **No intervention** | **12** | **Resistance exercise** | **3** |
| **Dulac (2020) (Casein) [70]*** | **protein 30.3 g/d**  **(casein 10.1g, maltodextrin 8.5 g, lipid 0.3 g) ×3 times/d** | **protein 0 g/d**  **maltodextrin 19.4 g**  **×3 times/d** | **Supplementation** | **Casein** | **No intervention** | **12** | **Resistance exercise & other exercise** | **3** |
| **Dulac (2020) (Whey) [70]*** | **protein 30 g/d**  **(whey 10g, maltodextrin 8.4 g, lipid 0.4 g)**  **×3 times/d** | **protein 0 g/d**  **maltodextrin 19.4 g**  **×3 times/d** | **Supplementation** | **Whey** | **No intervention** | **12** | **Resistance exercise & other exercise** | **3** |
| **Forbes (2020) (WPC Female) [71]*** | **protein 1.7 g/kg/d**  **WPC (<80% protein) protein 2.0 g/kg/d, at training days (6 d/wk)** | **protein 0 g/d**  **non-caloric fruit flavored drink mixture** | **Supplementation** | **Whey** | **No intervention** | **6** | **Resistance exercise & other exercise** | **6** |
| **Forbes (2020) (WPC Male) [71]*** | **protein 2.1 g/kg/d**  **WPC (<80% protein) protein 2.4 g/kg/d, at training days (6 d/wk)** | **protein 0 g/d**  **non-caloric fruit flavored drink mixture** | **Supplementation** | **Whey** | **No intervention** | **6** | **Resistance exercise & other exercise** | **6** |
| **Forbes (2020) (WPI Female) [71]*** | **protein 1.7 g/kg/d**  **WPI (>90% protein)**  **protein 2.0 g/kg/d, at training days (6 d/wk)** | **protein 0 g/d**  **non-caloric fruit flavored drink mixture** | **Supplementation** | **Whey** | **No intervention** | **6** | **Resistance exercise & other exercise** | **6** |
| **Forbes (2020) (WPI Male) [71]*** | **protein 2.1 g/kg/d**  **WPI (>90% protein)**  **protein 2.4 g/kg/d, at training days (6 d/wk)** | **protein 0 g/d**  **non-caloric fruit flavored drink mixture** | **Supplementation** | **Whey** | **No intervention** | **6** | **Resistance exercise & other exercise** | **6** |
| **Krull (2020) [72]*** | **protein21 g/d**  **WPI** | **protein 0 g/d**  **sucrose** | **Supplementation** | **Whey** | **No intervention** | **24** | **Resistance exercise** | **3** |
| **Kim**  **(2021)**  **(Evening)[73]*** | **protein 10 g/d**  **(milk protein)**  **in the evening (6:00–10:00 p.m.)** | **protein 0 g/d carbohydrate**  **in the evening (6:00–10:00 p.m.)** | **Supplementation** | **Milk** | **No intervention** | **12** | **No instructions** | **0** |
| **Kim**  **(2021)**  **(Morning)[73]*** | **protein 10 g/d**  **(milk protein)**  **in the morning (6:00–10:00 a.m.)** | **protein 0 g/d carbohydrate**  **in the morning (6:00–10:00 a.m.)** | **Supplementation** | **Milk** | **No intervention** | **12** | **No instructions** | **0** |
| **Griffen (2022) (with RT) [74]*** | **protein 50 g/d**  **WPI 25 g ×2 times/d** | **protein 0 g/d maltodextrin** | **Supplementation** | **Whey** | **No intervention** | **12** | **Resistance exercise** | **2** |
| **Griffen (2022) (without RT) [74]*** | **protein 50 g/d**  **WPI 25 g ×2 times/d** | **protein 0 g/d maltodextrin** | **Supplementation** | **Whey** | **No intervention** | **12** | **No instructions** | **0** |
| **Sexton**  **(2021)**  **(Female)**  **[75]*** | **protein 30 g/d**  **peanut protein** | **protein 0 g/d**  **abandon the use of a placebo** | **Supplementation** | **Peanut** | **No intervention** | **10** | **Resistance exercise** | **2** |
| **Sexton**  **(2021)**  **(Male)**  **[75]*** | **protein 30 g/d**  **peanut protein** | **protein 0 g/d**  **abandon the use of a placebo** | **Supplementation** | **Peanut** | **No intervention** | **10** | **Resistance exercise** | **2** |
| **Ullevig**  **(2021)**  **[76]*** | **protein 20.6 g/d**  **dried egg white** | **protein 0 g/d**  **maltodextrin** | **Supplementation** | **Egg** | **No intervention** | **26** | **No instructions** | **0** |
| **McKenna**  **(2021)**  **[77]*** | **protein 1.6 g/kg/d**  **lean beef protein**  **30 g/d at non training day, 62 g/d at training days (3 d/wk)** | **protein 1.0 g/kg/d**  **lean beef protein**  **15 g/d at non training day, 31 g/d at training days (3 d/wk)** | **Meal + Supplementation** | **Beef** | **No intervention** | **10** | **Resistance exercise** | **3** |
| **Li(2021)**  **(Blend)**  **[78]*** | **protein 16.78 g/d**  **whey-soy (1:1 ratio) blended protein 8.39 g ×2 times/d** | **protein 0 g/d**  **abandon the use of a placebo** | **Supplementation** | **Mix** | **No intervention** | **26** | **No instructions** | **0** |
| **Li(2021)**  **(Soy)**  **[78]*** | **protein 17.60 g/d**  **soy protein**  **8.80 g ×2 times/d** | **protein 0 g/d**  **abandon the use of a placebo** | **Supplementation** | **Soy** | **No intervention** | **26** | **No instructions** | **0** |
| **Li(2021)**  **(Whey)**  **[78]*** | **protein 15.96 g/d**  **whey protein**  **7.98 g ×2 times/d** | **protein 0 g/d**  **abandon the use of a placebo** | **Supplementation** | **Whey** | **No intervention** | **26** | **No instructions** | **0** |
| **Mertz**  **(2021)**  **(Collagen)[79]*** | **protein 40 g/d**  **bovine collagen protein hydrolysate 20 g × 2 times/d** | **protein 0 g/d maltodextrin** | **Supplementation** | **Collagen** | **No intervention** | **52** | **No instructions** | **0** |
| **Mertz**  **(2021)**  **(Whey)**  **[79]*** | **protein 40 g/d**  **whey protein hydrolysate 20 g × 2 times/d** | **protein 0 g/d maltodextrin** | **Supplementation** | **Collagen** | **No intervention** | **52** | **No instructions** | **0** |
| **Nygård**  **(2021)**  **[80]*** | **protein 3 g/d**  **marine protein hydrolysate 1.5 g × 2 times/d** | **protein 0 g/d**  **gum arabic** | **Supplementation** | **Fish** | **No intervention** | **52** | **No instructions** | **0** |
| **Lamb**  **(2020)**  **[81]*** | **protein 30 g/d**  **peanut protein** | **protein 0 g/d**  **abandon the use of a placebo** | **Supplementation** | **Peanut** | **No intervention** | **7.7** | **Resistance exercise** | **2** |
| **Boutry-Regard**  **(2020)**  **[82]** | **protein 20 g/d**  **WPI** | **protein 0 g/d**  **carbohydrate** | **Supplementation** | **Whey** | **No intervention** | **12** | **No instructions** | **0** |

**All trials were used to create a forest plot to evaluate the effect of added protein intakes on muscle strength percentage changes compared with control groups; Studies marked with asterisk were also used to create spline models to evaluate the relationship between total protein intake and muscle strength percentage changes from baselines.**

References

1. Castaneda C, Charnley JM, Evans WJ, Crim MC. Elderly women accommodate to a low-protein diet with losses of body cell mass, muscle function, and immune response. Am J Clin Nutr 1995; 62: 30-39

2. Rankin JW, Goldman LP, Puglisi MJ, et al. Effect of post-exercise supplement consumption on adaptations to resistance training. J Am Coll Nutr 2004; 23: 322-330

3. Candow DG, Chilibeck PD, Facci M, et al. Protein supplementation before and after resistance training in older men. Eur J Appl Physiol 2006; 97: 548-556

4. Candow DG, Burke NC, Smith-Palmer T, et al. Effect of whey and soy protein supplementation combined with resistance training in young adults. Int J Sport Nutr Exerc Metab 2006; 16: 233-244

5. Kerksick CM, Rasmussen CJ, Lancaster SL, et al. The effects of protein and amino acid supplementation on performance and training adaptations during ten weeks of resistance training. J Strength Cond Res 2006; 20: 643-653

6. Cribb PJ, Williams AD, Stathis CG, et al. Effects of whey isolate, creatine, and resistance training on muscle hypertrophy. Med Sci Sports Exerc 2007; 39: 298-307

7. Hartman JW, Tang JE, Wilkinson SB, et al. Consumption of fat-free fluid milk after resistance exercise promotes greater lean mass accretion than does consumption of soy or carbohydrate in young, novice, male weightlifters. Am J Clin Nutr 2007; 86: 373-381

8. Hoffman JR, Ratamess NA, Kang J, et al. Effects of protein supplementation on muscular performance and resting hormonal changes in college football players. J Sports Sci Med 2007; 6: 85-92

9. Iglay HB, Thyfault JP, Apolzan JW, et al. Resistance training and dietary protein: effects on glucose tolerance and contents of skeletal muscle insulin signaling proteins in older persons. Am J Clin Nutr 2007; 85: 1005-1013

10. Hoffman JR, Ratamess NA, Tranchina CP, et al. Effect of protein-supplement timing on strength, power, and body-composition changes in resistance-trained men. Int J Sport Nutr Exerc Metab 2009; 19: 172-185

11. Hulmi JJ, Kovanen V, Selänne, H, et al. Acute and long-term effects of resistance exercise with or without protein ingestion on muscle hypertrophy and gene expression. Amino Acids 2009; 37: 297-308

12. Shinkai S, Watanabe N, Lee S, et al. Randomized controlled trial on the effects of resistance training with or without nutritional supplementation of soy peptide for the frail elderly. Jpn J Nutr Diet 2009; 67: 76-83

13. Verdijk LB, Jonkers RA, Gleeson BG, et al. Protein supplementation before and after exercise does not further augment skeletal muscle hypertrophy after resistance training in elderly men. Am J Clin Nutr 2009; 89: 608-616

14. Bemben MG, Witten MS, Carter JM, et al. The effects of supplementation with creatine and protein on muscle strength following a traditional resistance training program in middle-aged and older men. J Nutr Health Aging 2010: 14: 155-159

15. Josse AR, Tang JE, Tarnopolsky MA, et al. Body composition and strength changes in women with milk and resistance exercise. Med Sci Sports Exerc 2010; 42: 1122-1130

16. Arazi H, Hakimi M, Hoseini K. The effects of whey protein supplementation on performance and hormonal adaptations following resistance training in novice men. Baltic J Health Phys Act 2011; 3: 87-95

17. Deibert P, Solleder F, Konig D, et al. Soy protein based supplementation supports metabolic effects of resistance training in previously untrained middle aged males. Aging Male 2011; 14: 273-279

18. Alemán-Mateo H, Macías L, Esparza-Romero J, et al. Physiological effects beyond the significant gain in muscle mass in sarcopenic elderly men: evidence from a randomized clinical trial using a protein-rich food. Clin Interv Aging 2012; 7: 225-234

19. Erskine RM, Fletcher G, Hanson B, et al. Whey protein does not enhance the adaptations to elbow flexor resistance training. Med Sci Sports Exerc 2012; 44: 1791-1800

20. Farnfield MM, Breen L, Carey KA, et al. Activation of mTOR signalling in young and old human skeletal muscle in response to combined resistance exercise and whey protein ingestion. Appl Physiol Nutr Metab 2012; 37: 21-30

21. Hida A, Hasegawa Y, Mekata Y, et al. Effects of egg white protein supplementation on muscle strength and serum free amino acid concentrations. Nutrients 2012; 4: 1504-1517

22. Tieland M, Dirks ML, van der Zwaluw N, et al. Protein supplementation improves physical performance in frail elderly people: a randomized, double-blind, placebo-controlled trial. J Am Med Dir Assoc 2012; 13: 720-726

23. Tieland M, Dirks ML, van der Zwaluw N, et al. Protein supplementation increases muscle mass gain during prolonged resistance-type exercise training in frail elderly people: a randomized, double-blind, placebo-controlled trial. J Am Med Dir Assoc 2012; 13: 713-719

24. Weinheimer EM, Conley TB, Kobza VM, et al. Whey protein supplementation does not affect exercise training-induced changes in body composition and indices of metabolic syndrome in middle-aged overweight and obese adults. J Nutr 2012; 142: 1532-1539

25. Weisgarber KD, Candow DG, Vogt ES. Whey protein before and during resistance exercise has no effect on muscle mass and strength in untrained young adults. Int J Sport Nutr Exerc Metab 2012; 22: 463-469

26. Arnarson A, Gudny Geirsdottir O, Ramel A, et al. Effects of whey proteins and carbohydrates on the efficacy of resistance training in elderly people: double blind, randomised controlled trial. Eur J Clin Nutr 2013; 67: 821-826

27. Chalé A, Cloutier GJ, Hau C, et al. Efficacy of whey protein supplementation on resistance exercise-induced changes in lean mass, muscle strength, and physical function in mobility-limited older adults. J Gerontol A Biol Sci Med Sci 2013; 68: 682-690

28. Herda AA, Herda TJ, Costa PB, et al. Muscle performance, size, and safety responses after eight weeks of resistance training and protein supplementation: a randomized, double-blinded, placebo-controlled clinical trial. J Strength Cond Res 2013; 27: 3091-3100

29. Leenders M, Verdijk LB, Van der Hoeven L, et al. Protein supplementation during resistance-type exercise training in the elderly. Med Sci Sports Exerc 2013; 45: 542-552

30. Volek JS, Volk BM, Gomez AL, et al. Whey protein supplementation during resistance training augments lean body mass. J Am Coll Nutr 2013; 32: 122-135

31. Alemán-Mateo H, Carreón VR, Macías L, et al. Nutrient-rich dairy proteins improve appendicular skeletal muscle mass and physical performance, and attenuate the loss of muscle strength in older men and women subjects: a single-blind randomized clinical trial. Clin Interv Aging 2014; 9: 1517-1525

32. Babault N, Deley G, Le Ruyet P, et al. Effects of soluble milk protein or casein supplementation on muscle fatigue following resistance training program: a randomized, double-blind, and placebo-controlled study. J Int Soc Sports Nutr 2014; 11: 36

33. Farup J, Rahbek SK, Vendelbo MH, et al. Whey protein hydrolysate augments tendon and muscle hypertrophy independent of resistance exercise contraction mode. Scand J Med Sci Sports 2014; 24: 788-798

34. Figueroa A, Wong A, Kinsey A, et al. Effects of milk proteins and combined exercise training on aortic hemodynamics and arterial stiffness in young obese women with high blood pressure. Am J Hypertens 2014; 27: 338-344

35. Gryson C, Ratel S, Rance M, et al. Four-month course of soluble milk proteins interacts with exercise to improve muscle strength and delay fatigue in elderly participants. J Am Med Dir Assoc 2014; 15: 958.e1-9

36. Mori H, Niwa M. Effect of nutritional care and whey protein supplementation on the body composition and physical function in older adults after combined resistance and aerobic exercise. Jpn J Nutr Diet 2014; 72: 12-20

37. Negro M, Vandoni M, Ottobrini S, et al. Protein supplementation with low fat meat after resistance training: effects on body composition and strength. Nutrients 2014; 6: 3040-3049

38. Babault N, Païzis C, Deley G, et al. Pea proteins oral supplementation promotes muscle thickness gains during resistance training: a double-blind, randomized, placebo-controlled clinical trial vs. whey protein. J Int Soc Sports Nutr 2015; 12: 3

39. Hulmi JJ, Laakso M, Mero AA, et al. The effects of whey protein with or without carbohydrates on resistance training adaptations. J Int Soc Sports Nutr 2015; 12: 48

40. Ormsbee MJ, Kinsey AW, Eddy WR, et al. The influence of nighttime feeding of carbohydrate or protein combined with exercise training on appetite and cardiometabolic risk in young obese women. Appl Physiol Nutr Metab 2015; 40: 37-45

41. Snijders T, Res PT, Smeets JS, et al. Protein ingestion before sleep increases muscle mass and strength gains during Prolonged resistance-type exercise training in healthy young men. J Nutr 2015; 145: 1178-1184

42. Zhu K, Kerr DA, Meng X, et al. Two-year whey protein supplementation did not enhance muscle mass and physical function in well-nourished healthy older postmenopausal women. J Nutr 2015; 145: 2520-2526

43. Maltais ML, Ladouceur JP, Dionne IJ. The effect of resistance training and different sources of postexercise protein supplementation on muscle mass and physical capacity in sarcopenic elderly men. J Strength Cond Res 2016; 30: 1680-1687

44. Paoli A, Pacelli QF, Cancellara P, et al. Protein supplementation does not further increase latissimus dorsi muscle fiber hypertrophy after eight weeks of resistance training in novice subjects, but partially counteracts the fast-to-slow muscle fiber transition. Nutrients 2016; 8: 331

45. Reidy PT, Borack MS, Markofski MM, et al. Protein supplementation has minimal effects on muscle adaptations during resistance exercise training in young men: a double-blind randomized clinical trial. J Nutr 2016; 146: 1660-1669

46. Taylor LW, Wilborn C, Roberts MD, et al. Eight weeks of pre- and postexercise whey protein supplementation increases lean body mass and improves performance in Division III collegiate female basketball players. Appl Physiol Nutr Metab 2016; 41: 249-254

47. Thomson RL, Brinkworth GD, Noakes M, et al. Muscle strength gains during resistance exercise training are attenuated with soy compared with dairy or usual protein intake in older adults: a randomized controlled trial. Clin Nutr 2016; 35: 27-33

48. Hwang PS, Andre TL, McKinley-Barnard SK, et al. Resistance training-induced elevations in muscular strength in trained men are maintained after 2 weeks of detraining and not differentially affected by whey protein supplementation. J Strength Cond Res 2017; 31: 869-881

49. Niccoli S, Kolobov A, Bon T, et al. Whey protein supplementation improves rehabilitation outcomes in hospitalized geriatric patients: a double blinded, randomized controlled trial. J Nutr Gerontol Geriatr 2017; 36: 149-165

50. Ottestad I, Lovstad AT, Gjevestad GO, et al. Intake of a protein-enriched milk and effects on muscle mass and strength. A 12-week randomized placebo controlled trial among community-dwelling older adults. J Nutr Health Aging 2017; 21: 1160-1169

51. Rossato LT, Nahas PC, de Branco FMS, et al. Higher protein intake does not improve lean mass gain when compared with RDA recommendation in postmenopausal women following resistance exercise protocol: a randomized clinical trial. Nutrients 2017; 9: 1007

52. Vorup J, Pedersen MT, Brahe LK, et al. Effect of small-sided team sport training and protein intake on muscle mass, physical function and markers of health in older untrained adults: a randomized trial. PLoS One 2017; 12: e0186202

53. Bhasin S, Apovian CM, Travison TG, et al. Effect of protein intake on lean body mass in functionally limited older men: a randomized clinical trial. JAMA Intern Med 2018; 178: 530-541

54. Mitchell CJ, D’Souza DF, Mitchell SM, et al. Impact of dairy protein during limb immobilization and recovery on muscle size and protein synthesis; a randomized controlled trial. J Appl Physiol 2018; 124: 717-728

55. Nobuco HCG, Tomeleri CM, Sugihara Junior P, et al. Effects of whey protein supplementation pre- or post-resistance training on muscle mass, muscular strength, and functional capacity in pre-conditioned older women: a randomized clinical trial. Nutrients 2018; 10: 563

56. Orsatti FL, Maestá N, de Oliveira EP, et al. Adding soy protein to milk enhances the effect of resistance training on muscle strength in postmenopausal women. J Diet Suppl 2018; 15: 140-152

57. Park Y, Choi JE, Hwang HS. Protein supplementation improves muscle mass and physical performance in undernourished prefrail and frail elderly subjects: a randomized, double-blind, placebo-controlled trial. Am J Clin Nutr 2018; 108: 1026-1033

58. Sharp MH, Lowery RP, Shields KA, et al. The effects of beef, chicken, or whey protein after workout on body composition and muscle performance. J Strength Cond Res 2018; 32: 2233-2242

59. Sugihara Junior P, Ribeiro AS, Nabuco HCG, et al. Effects of whey protein supplementation associated with resistance training on muscular strength, hypertrophy, and muscle quality in preconditioned older women. Int J Sport Nutr Exerc Metab 2018; 28: 528-535

60. Bartholomae E, Incollingo A, Vizcaino M, et al. Mung bean protein supplement improves muscular strength in healthy, underactive vegetarian adults. Nutrients 2019; 11: 2423

61. Centner C, Zdzieblik D, Roberts L, et al. Effects of blood flow restriction training with protein supplementation on muscle mass and strength in older men. J Sports Sci Med 2019; 18: 471-478

62. Drotningsvik A, Oterhals Å, Flesland O, et al. Fish protein supplementation in older nursing home residents: a randomised, double-blind, pilot study. Pilot Feasibility Stud 2019; 5: 35

63. Jonvik KL, Paulussen KJM, Danen SL, et al. Protein supplementation does not augment adaptations to endurance exercise training. Med Sci Sports Exerc 2019; 51: 2041-2049

64. Nabuco HCG, Tomeleri CM, Fernandes RR, et al. Effect of whey protein supplementation combined with resistance training on body composition, muscular strength, functional capacity, and plasma-metabolism biomarkers in older women with sarcopenic obesity: A randomized, double-blind, placebo-controlled trial. Clin Nutr ESPEN 2019; 32: 88-95

65. Nahas PC, Rossato LT, Martins FM, et al. Moderate increase in protein intake promotes a small additional improvement in functional capacity, but not in muscle strength and lean mass quality, in postmenopausal women following resistance exercise: a randomized clinical trial. Nutrients 2019; 11: 1323

66. ten Haaf DSM, Eijsvogels TMH, Bongers C, et al. Protein supplementation improves lean body mass in physically active older adults: a randomized placebo-controlled trial. J Cachexia Sarcopenia Muscle 2019; 10: 298-310

67. Atherton C, McNaughton LR, Close GL, et al. Post-exercise provision of 40 g of protein during whole body resistance training further augments strength adaptations in elderly males. Res Sports Med 2020; 28: 469-483

68. Björkman MP, Suominen MH, Kautiainen H, et al. Effect of protein supplementation on physical performance in older people with sarcopenia-a randomized controlled trial. J Am Med Dir Assoc 2020; 21: 226-232

69. Duarte NM, Cruz AL, Silva DC, et al. Intake of whey isolate supplement and muscle mass gains in young healthy adults when combined with resistance training: a blinded randomized clinical trial (pilot study). Sports Med Phys Fitness 2020; 60: 75-84

70. Dulac MC, Pion CH, Lemieux FC, et al. Effects of slow- v. fast-digested protein supplementation combined with mixed power training on muscle function and functional capacities in older men. Br J Nutr 2020; 125: 1017-1033

71. Forbes J, Bell GJ. Whey protein isolate or concentrate combined with concurrent training does not augment performance, cardiorespiratory fitness, or strength adaptations. Sports Med Phys Fitness 2020; 60: 832-840

72. Krull MR, Howell CR, Partin RE, et al. Protein Supplementation and Resistance Training in Childhood Cancer Survivors. Med Sci Sports Exerc 2020; 52: 2069-2077

73. Kim HK, Chijiki H, Fukazawa M, Okubo J, Ozaki M, Nanba T, et al. Supplementation of Protein at Breakfast Rather Than at Dinner and Lunch Is Effective on Skeletal Muscle Mass in Older Adults. Frontiers in nutrition. 2021;8:797004.

74. Griffen C, Duncan M, Hattersley J, Weickert MO, Dallaway A, Renshaw D. Effects of resistance exercise and whey protein supplementation on skeletal muscle strength, mass, physical function, and hormonal and inflammatory biomarkers in healthy active older men: a randomised, double-blind, placebo-controlled trial. Experimental gerontology. 2022 Feb;158:111651.

75. Sexton CL, Smith MA, Smith KS, Osburn SC, Godwin JS, Ruple BA, et al. Effects of Peanut Protein Supplementation on Resistance Training Adaptations in Younger Adults. Nutrients. 2021 Nov 9;13(11).

76. Ullevig SL, Zuniga K, Austin Lobitz C, Santoyo A, Yin Z. Egg protein supplementation improved upper body muscle strength and protein intake in community-dwelling older adult females who attended congregate meal sites or adult learning centers: A pilot randomized controlled trial. Nutrition and health. 2021 Nov 3:2601060211051592.

77. McKenna C, Salvador A, Hughes R, Scaroni S, Alamilla R, Askow A, et al. Higher protein intake during resistance training does not potentiate strength, but modulates gut microbiota, in middle-aged adults: a randomized control trial. American Journal of Physiology-Endocrinology and Metabolism. 2021 03/08;320.

78. Li C, Meng H, Wu S, Fang A, Liao G, Tan X, et al. Daily Supplementation With Whey, Soy, or Whey-Soy Blended Protein for 6 Months Maintained Lean Muscle Mass and Physical Performance in Older Adults With Low Lean Mass. Journal of the Academy of Nutrition and Dietetics. 2021 Jun;121(6):1035-48.e6.

79. Mertz KH, Reitelseder S, Bechshoeft R, Bulow J, Højfeldt G, Jensen M, et al. The effect of daily protein supplementation, with or without resistance training for 1 year, on muscle size, strength, and function in healthy older adults: A randomized controlled trial. The American journal of clinical nutrition. 2021 Apr 6;113(4):790-800.

80. Nygård LK, Mundal I, Dahl L, Šaltytė Benth J, Rokstad AMM. Limited Benefit of Marine Protein Hydrolysate on Physical Function and Strength in Older Adults: A Randomized Controlled Trial. Marine drugs. 2021 Jan 27;19(2).

81. Lamb DA, Moore JH, Smith MA, Vann CG, Osburn SC, Ruple BA, et al. The effects of resistance training with or without peanut protein supplementation on skeletal muscle and strength adaptations in older individuals. Journal of the International Society of Sports Nutrition. 2020 Dec 14;17(1):66.

82. Boutry-Regard C, Vinyes-Parés G, Breuillé D, Moritani T. Supplementation with Whey Protein, Omega-3 Fatty Acids and Polyphenols Combined with Electrical Muscle Stimulation Increases Muscle Strength in Elderly Adults with Limited Mobility: A Randomized Controlled Trial. Nutrients. 2020 Jun 23;12(6).
