# Supplementary material for: Synergistic Effect of Increased Total Protein Intake and Strength Training on Muscle Strength: A Dose-Response Meta-analysis of Randomized Controlled Trials
Source: Sports Med Open. 2022 Sep 4;8:110. doi: 10.1186/s40798-022-00508-w (PMC9441410; doi:10.1186/s40798-022-00508-w)
Supplement: Supplementary file 4 — Additional file 4. Risk-of-bias assessment, Funnel plots of studies with or without resistance training for changes in muscle strength, Forest plot assessing the effect of protein supplementation on changes in muscle strength. [file 40798_2022_508_MOESM4_ESM.docx]

**Additional file 4**

**Synergistic effect of increased total protein intake and strength training on muscle strength:**

**A dose–response meta-analysis from randomized controlled trials**

Ryoichi Tagawa^1 #^

Daiki Watanabe^2, 3 #^

Kyoko Ito^1^

Takeru Otsuyama^1^

Kyosuke Nakayama^1^

Chiaki Sanbongi^1^

Motohiko Miyachi^2, 3 *^

^1^ Nutrition and Food Function Research Department, Food Microbiology and Function Research Laboratories, R&D Division, Meiji Co., Ltd., 1-29-1 Nanakuni, Hachioji, Tokyo 192-0919, Japan

^2^ Faculty of Sport Sciences, Waseda University, 2-579-15 Mikajima, Tokorozawa-city, Saitama 359-1192, Japan

^3^ Department of Physical Activity Research, National Institute of Health and Nutrition, National Institutes of Biomedical Innovation, Health and Nutrition, 1-23-1 Toyama, Shinjuku-ku, Tokyo 162-8636, Japan

# RT and DW equally contributed to the manuscript and are joint first authors.

* Corresponding Author: Motohiko Miyachi

Email: cardiovascular0327@mac.com


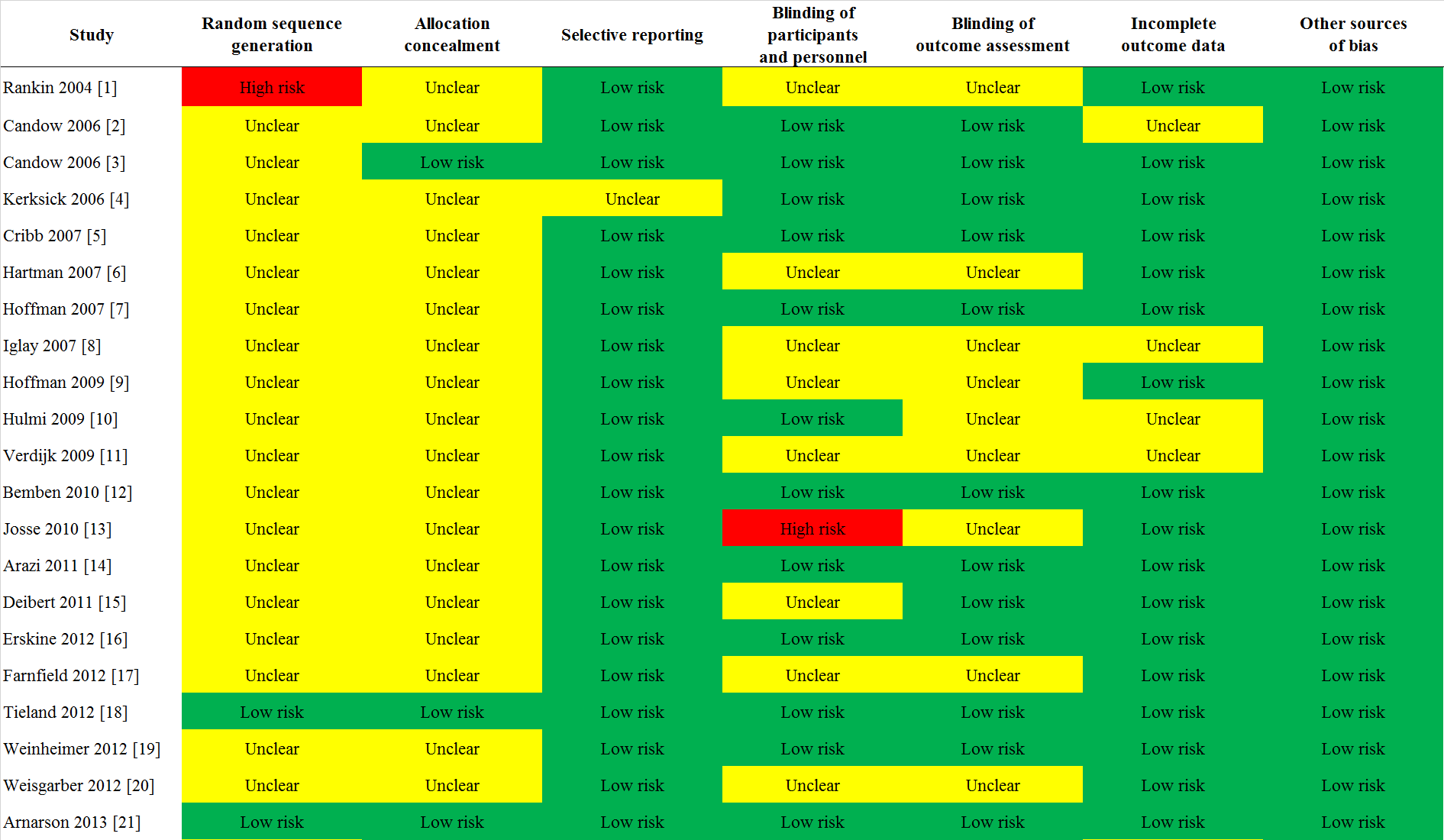


*Supplementary Figure S1* **Risk-of-bias assessment (1/4)**


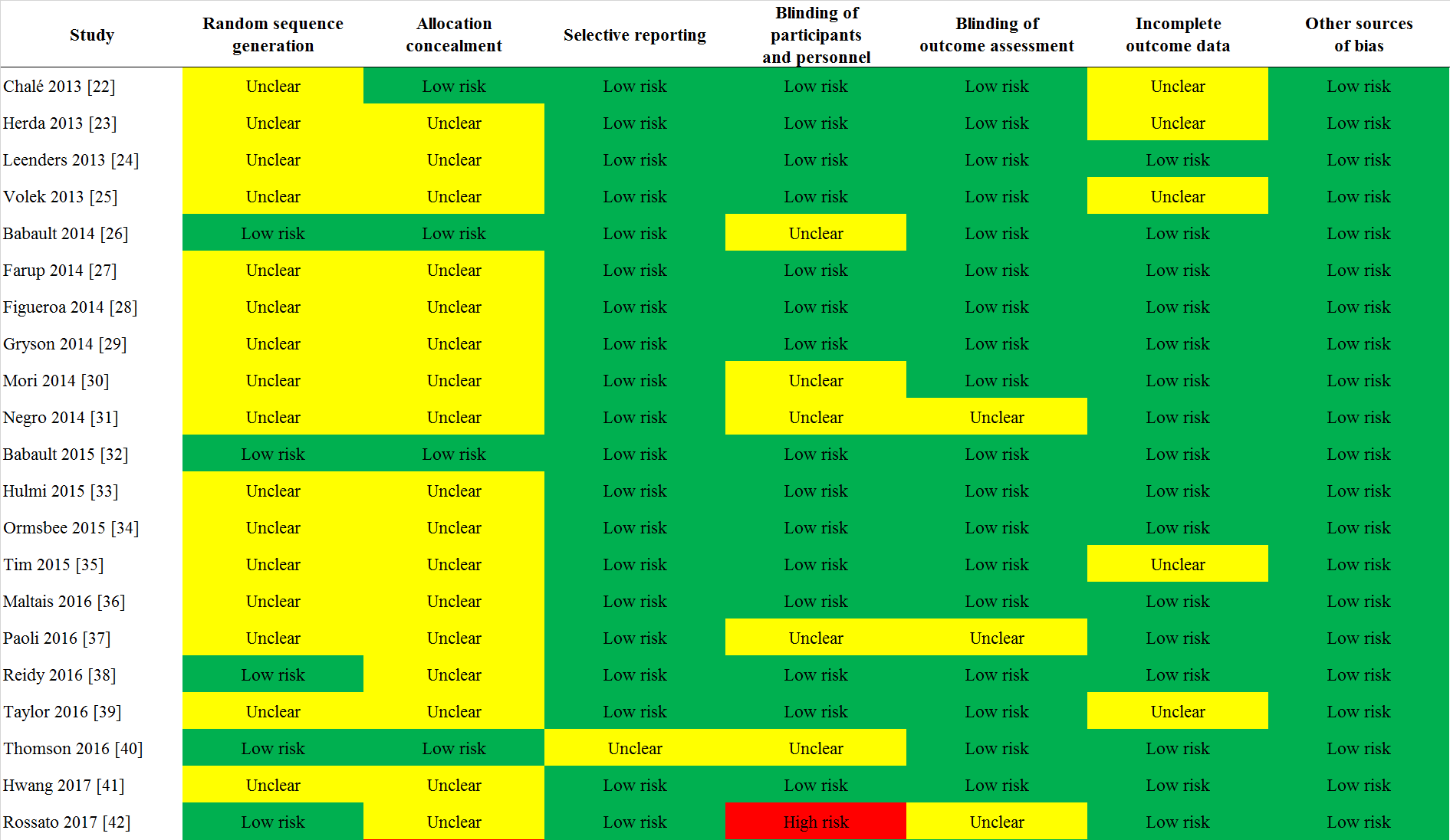


*Supplementary Figure S1* **Risk-of-bias assessment (2/4)**


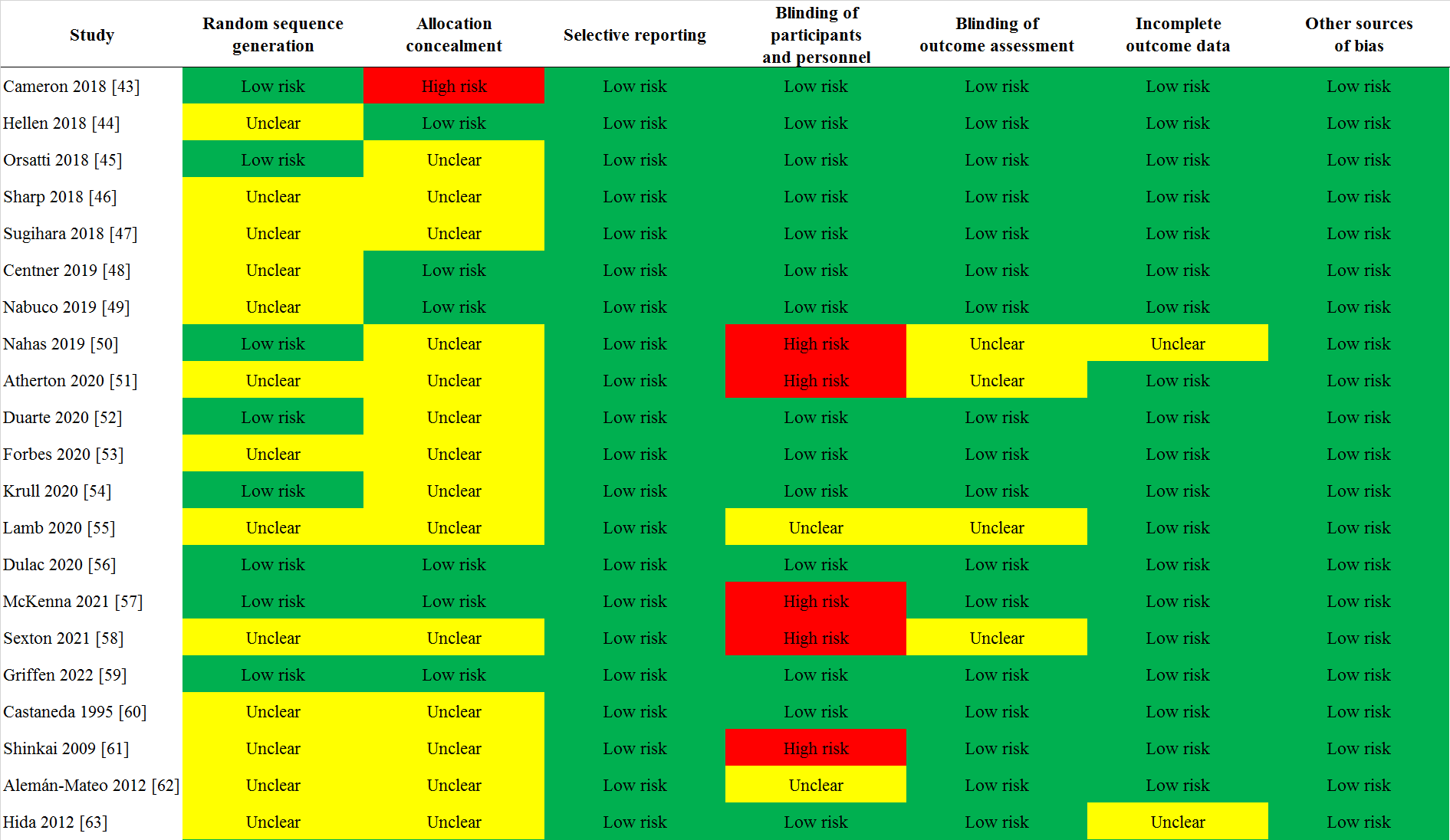


*Supplementary Figure S1* **Risk-of-bias assessment (3/4)**

**
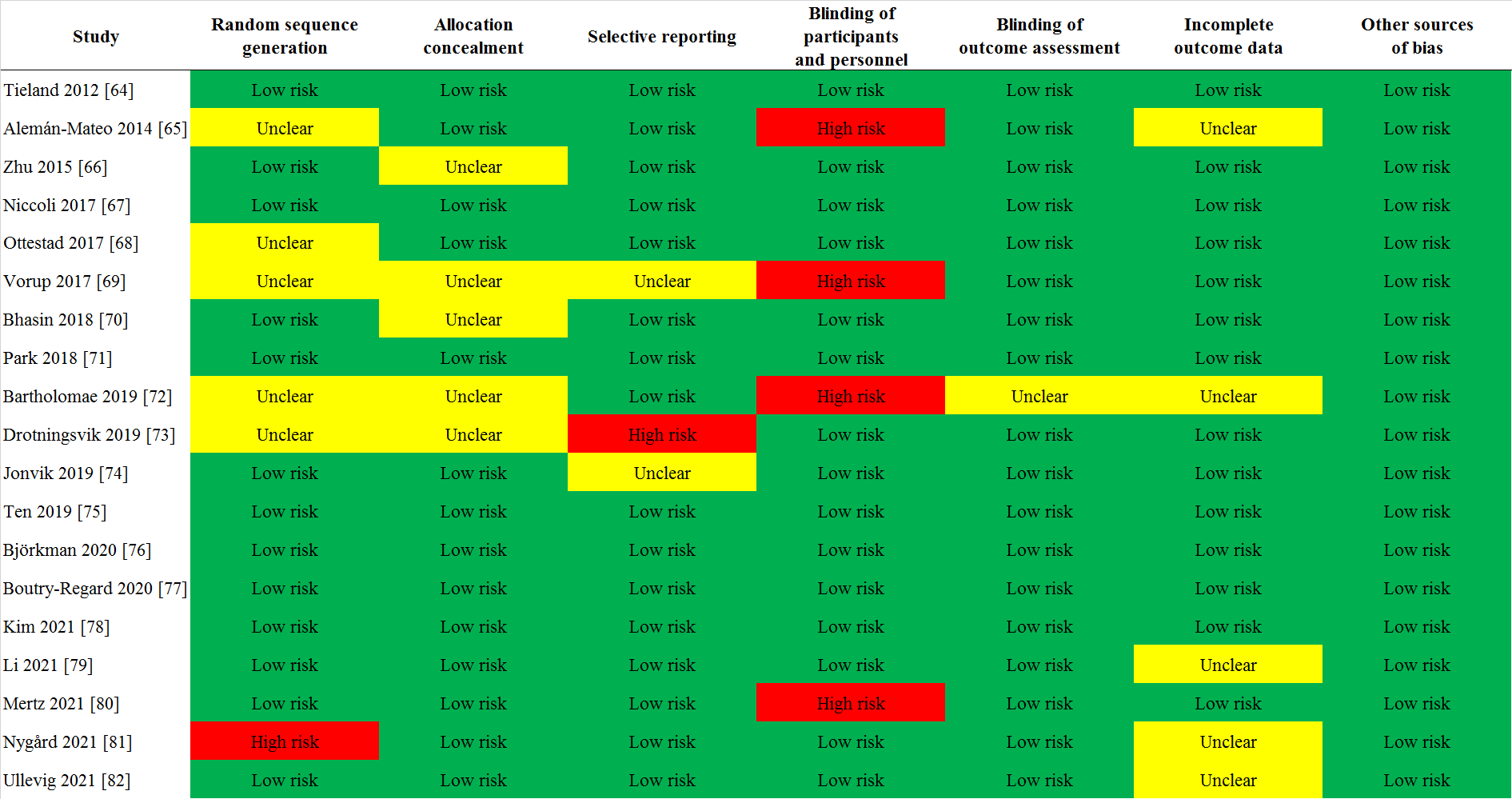
**

*Supplementary Figure S1* **Risk-of-bias assessment (4/4)**


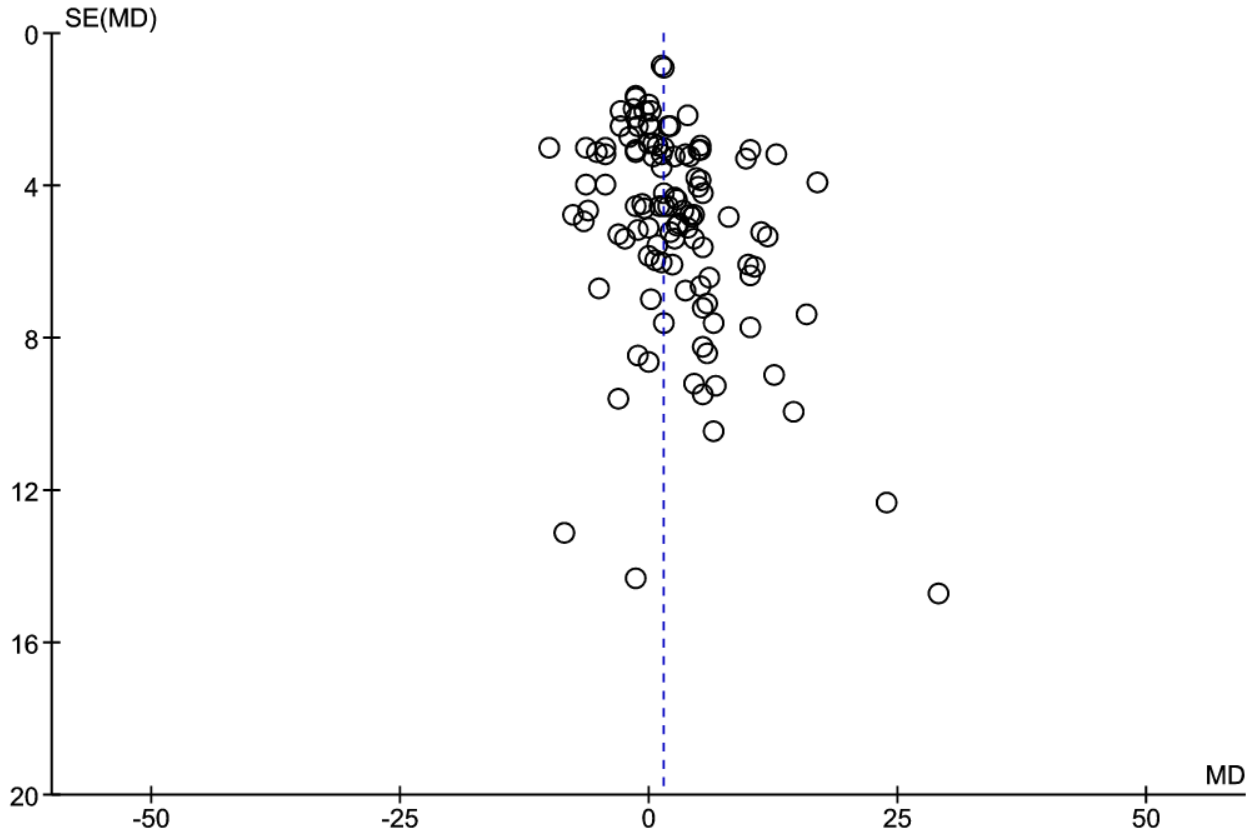


*Supplementary Figure S2 (A)* **Funnel plot of all included studies for changes in muscle strength.**

A random-effects model was used.

*Abbreviations*: MD, mean difference between groups; SE, standard error.


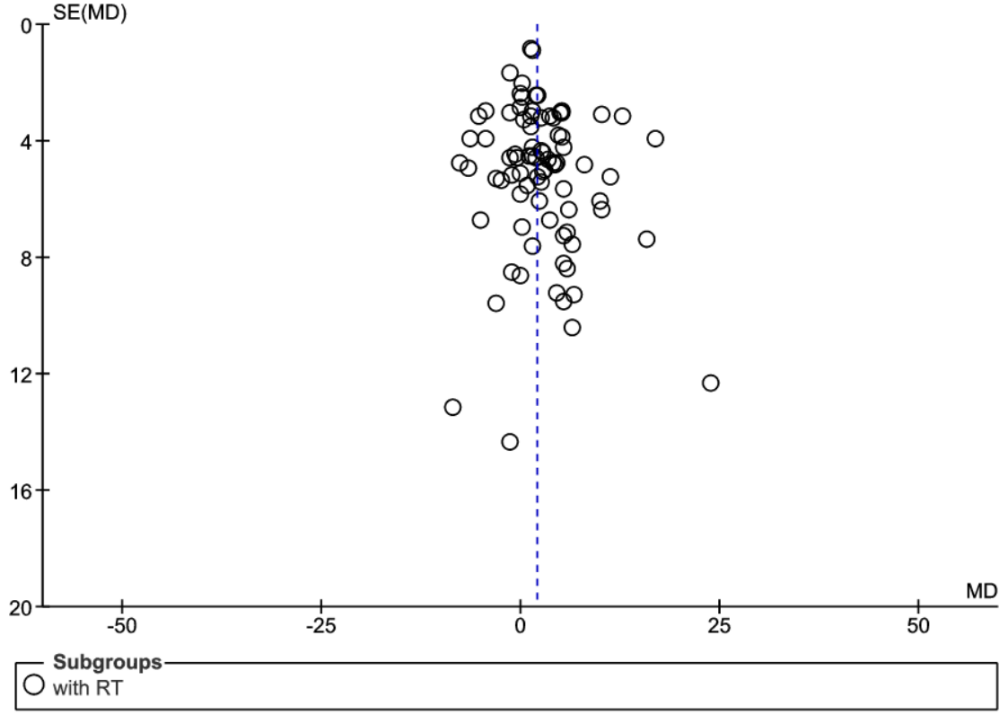

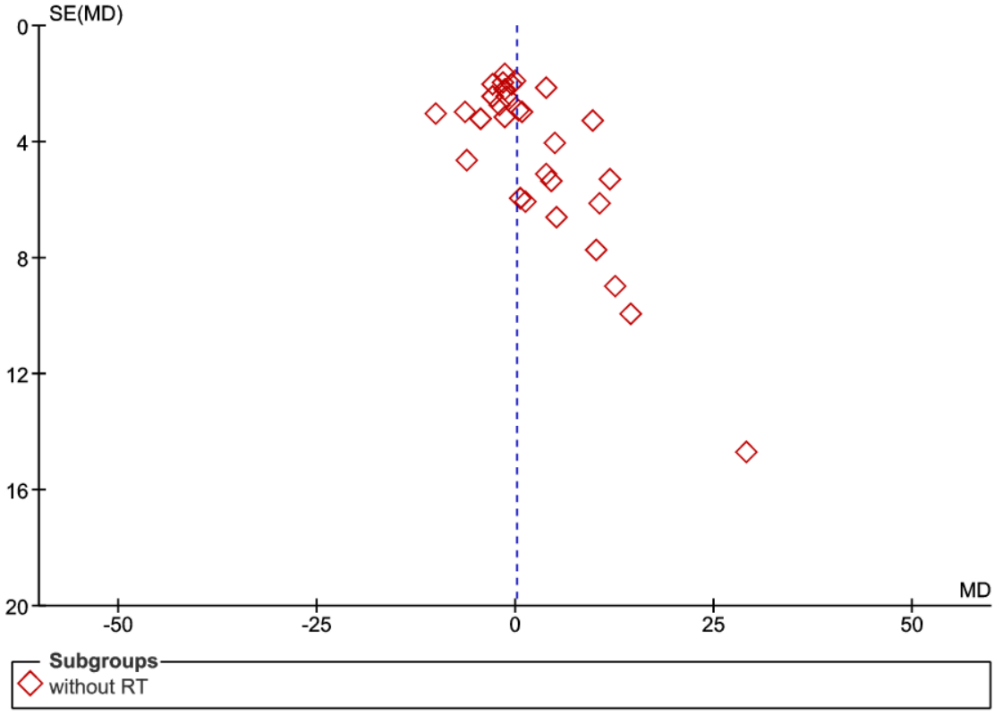


*Supplementary Figure S2 (B)* **Funnel plots of studies with (left) or without (right) resistance training for changes in muscle strength.**

A random-effects model was used.

*Abbreviations*: MD, mean difference between groups; SE, standard error.


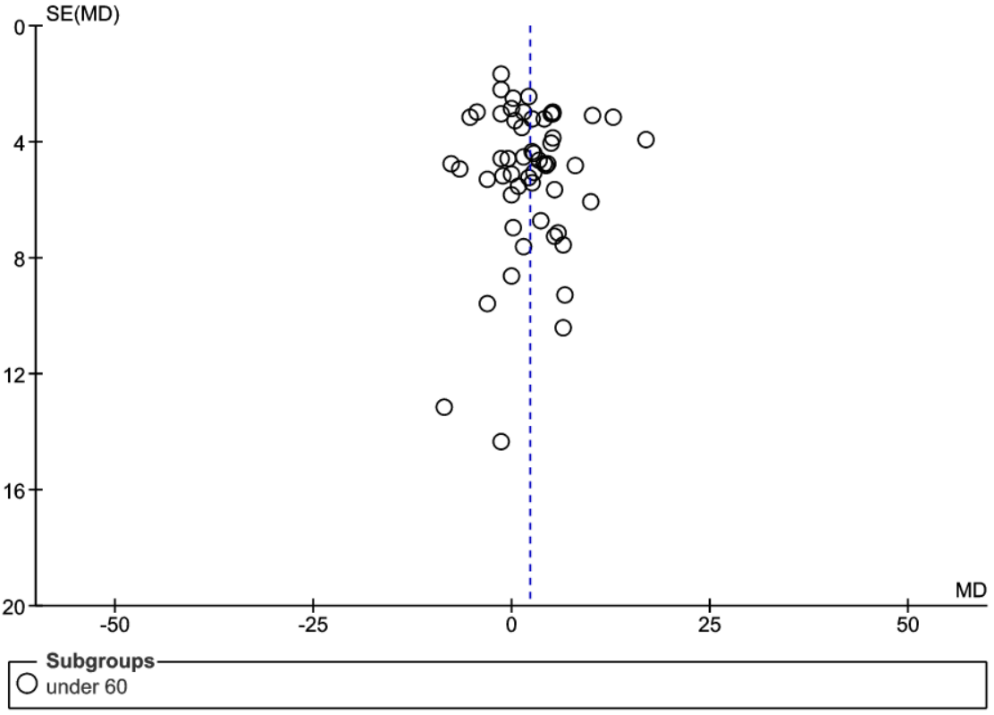

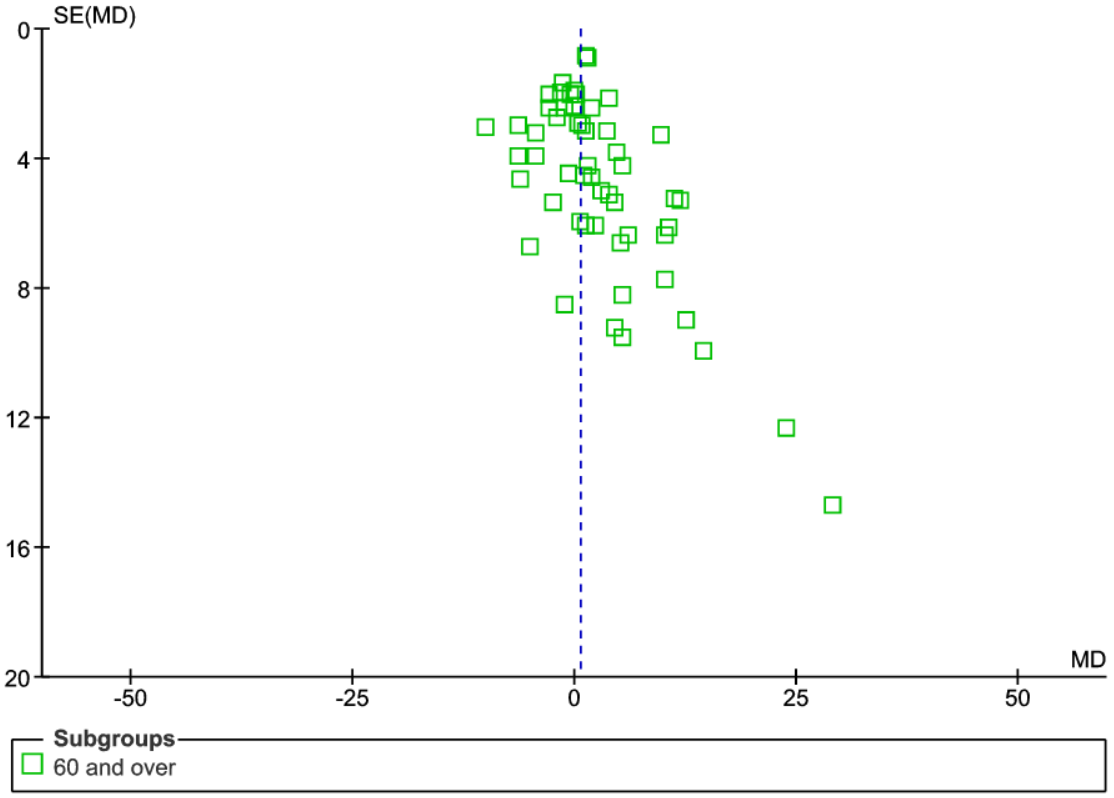


Supplementary Figure S2 (C) **Funnel plots of studies with participants aged under 60 (left) or 60 and over (right) for changes in muscle strength.**

A random-effects model was used.

Abbreviations: MD, mean difference between groups; SE, standard error.


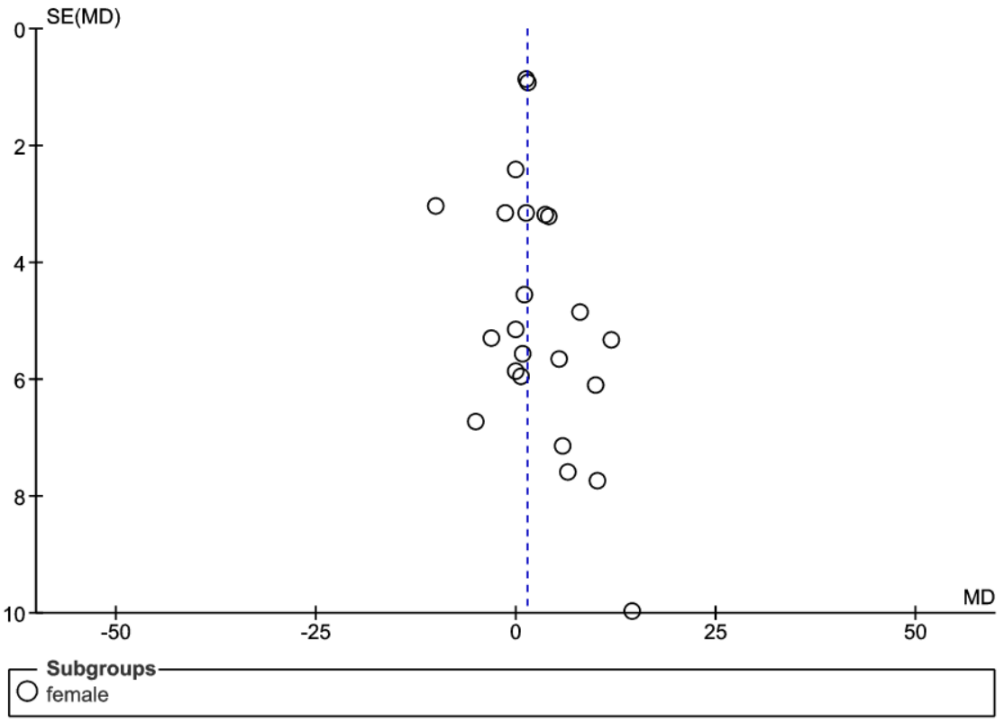

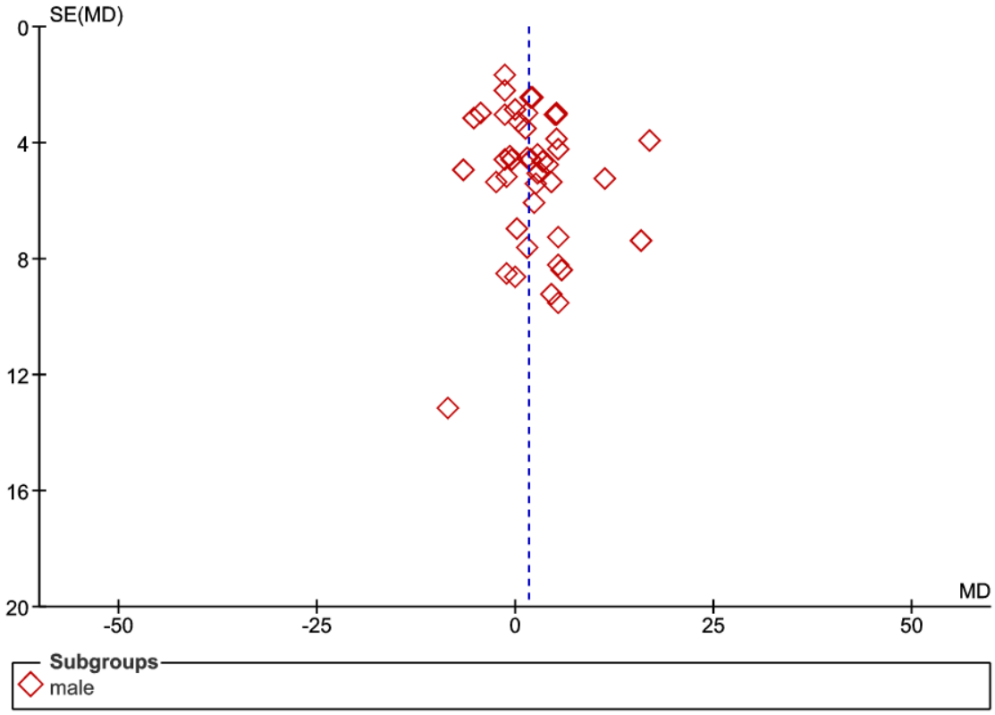


Supplementary Figure S2 (D) **Funnel plots of studies with female (left) or male (right) participants for changes in muscle strength.**

A random-effects model was used.

Abbreviations: MD, mean difference between groups; SE, standard error.


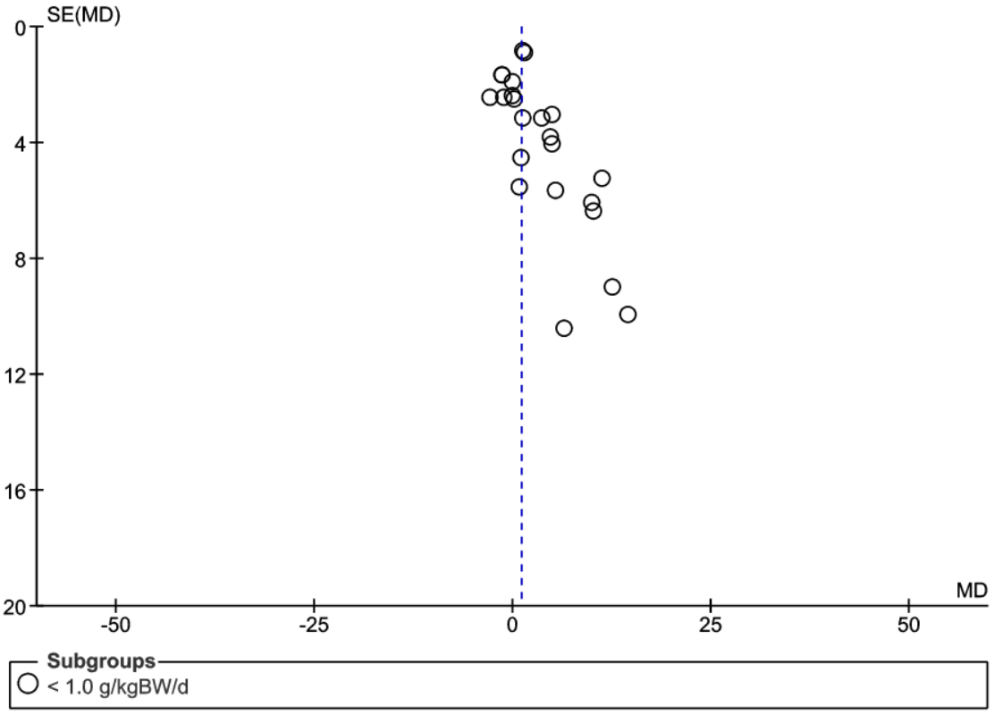

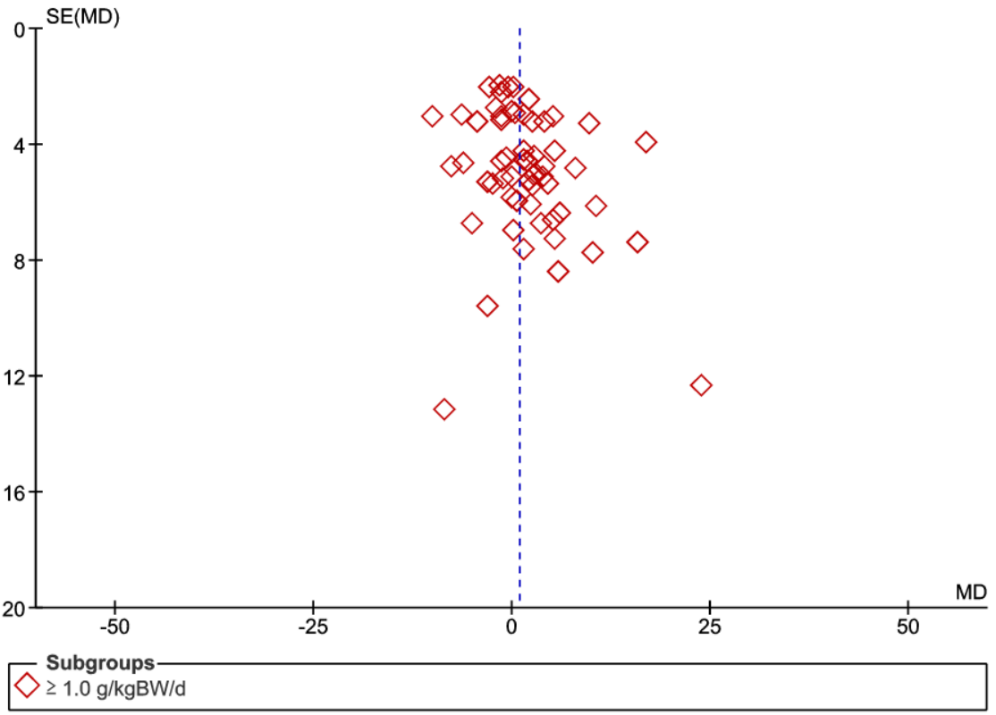


Supplementary Figure S2 (E) **Funnel plots of studies with baseline protein intake less than 1.0** **g/kg/day (left) or 1.0** **g/kg/day and over (right) for changes in muscle strength.**

A random-effects model was used.

Abbreviations: MD, mean difference between groups; SE, standard error.


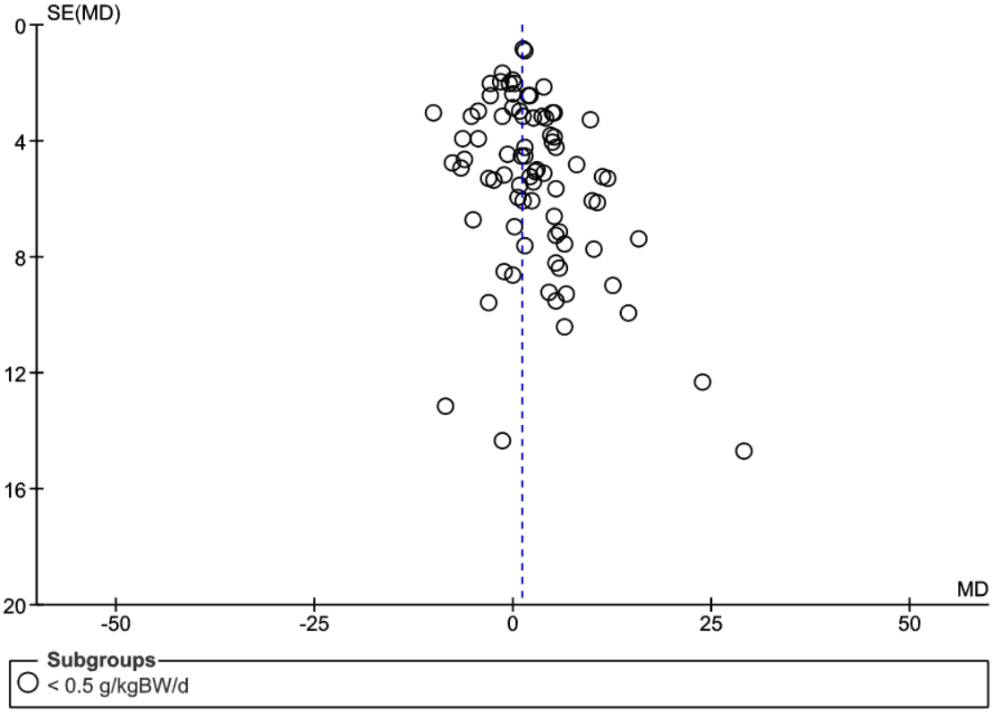

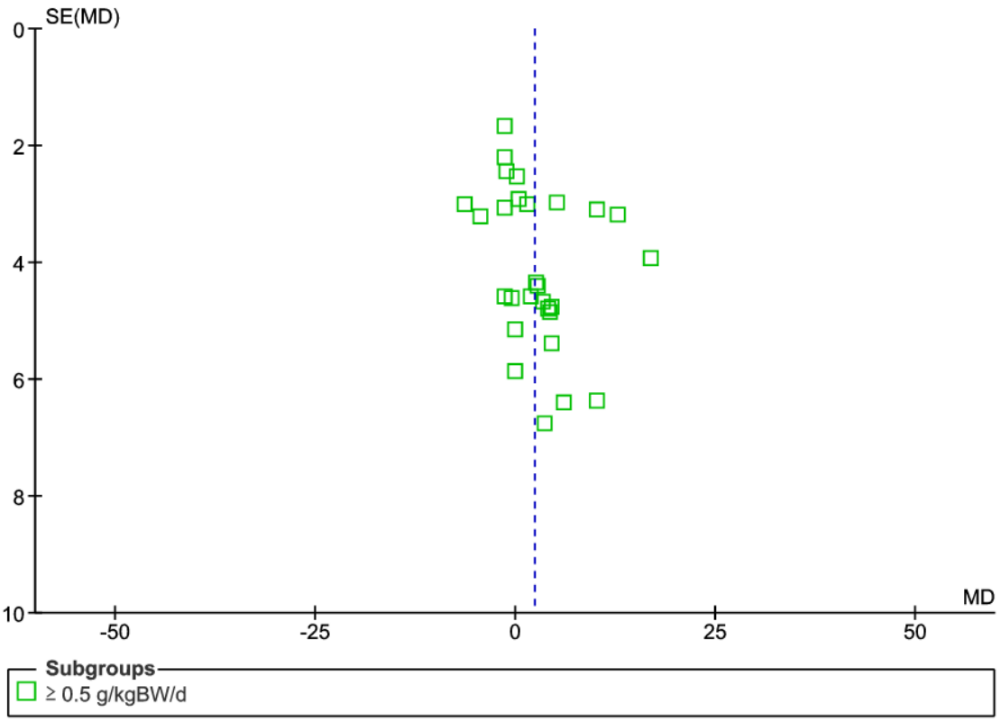


Supplementary Figure S2 (F) **Funnel plots of studies with added protein intake less than 0.5** **g/kg/day (left) or 0.5** **g/kg/day and over (right) for changes in muscle strength.**

A random-effects model was used.

Abbreviations: MD, mean difference between groups; SE, standard error.


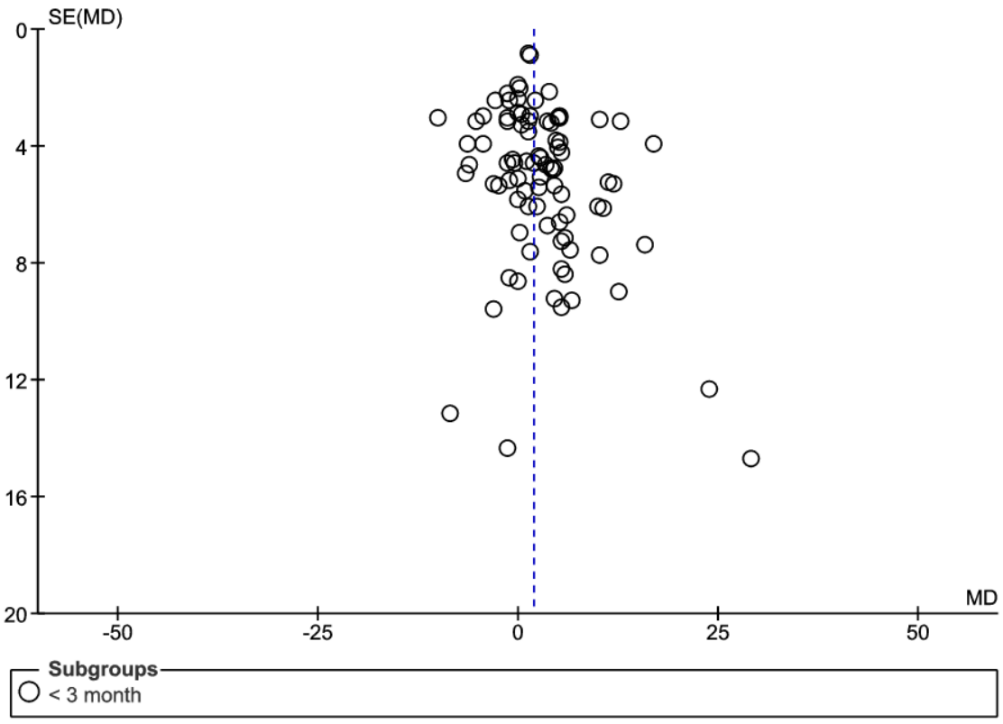

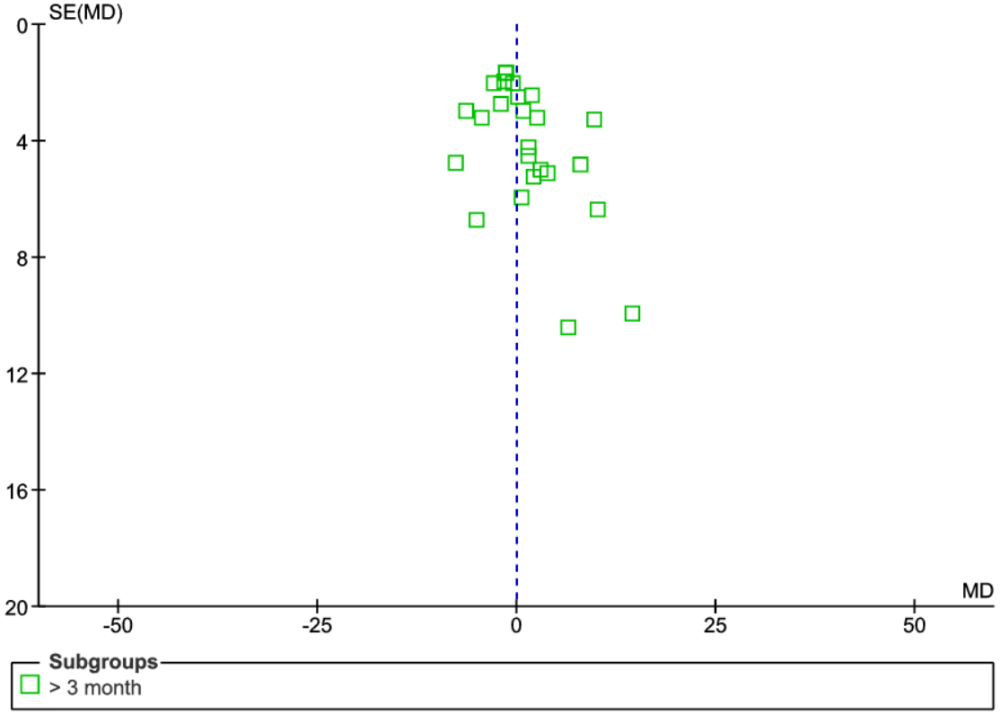


Supplementary Figure S2 (G) **Funnel plots of studies with trial period less than than 3 months (left) or 3 month and over (right) for changes in muscle strength.**

A random-effects model was used.

Abbreviations: MD, mean difference between groups; SE, standard error.


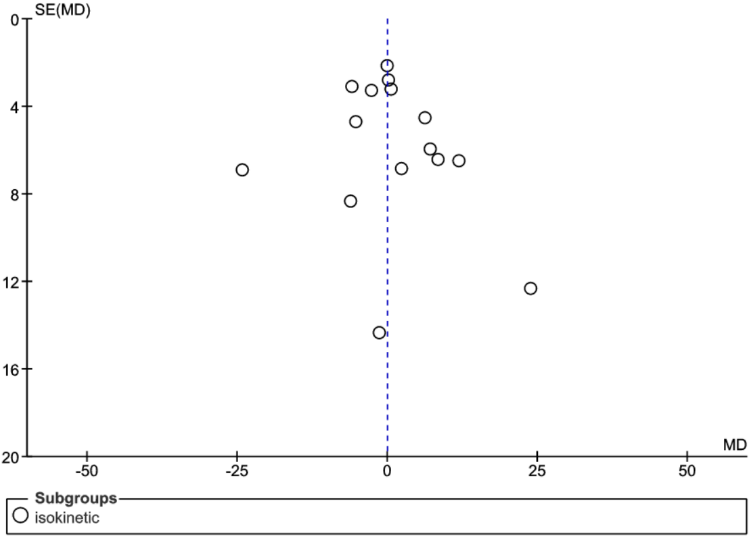

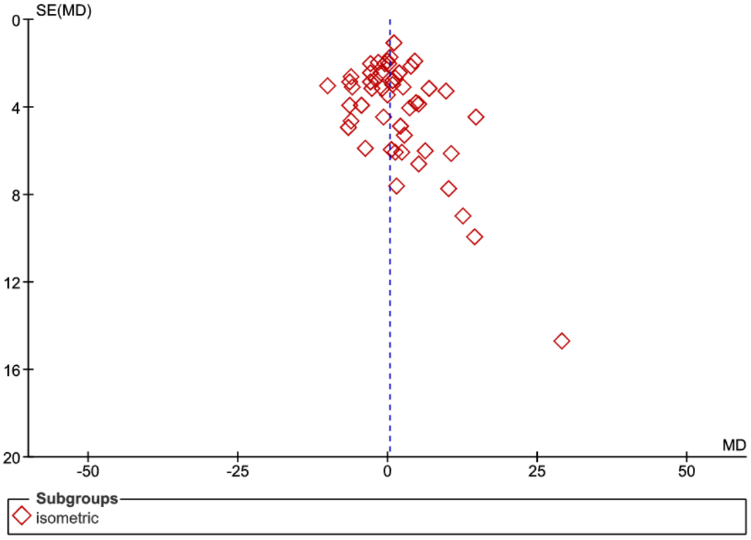

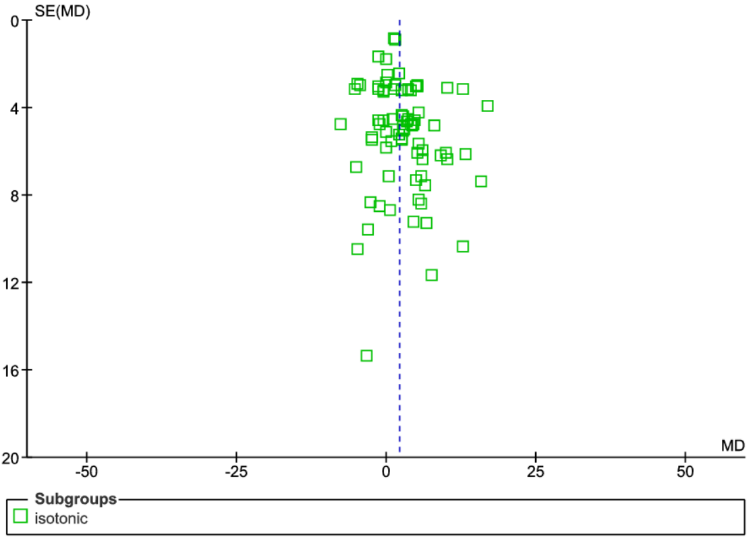


Supplementary Figure S2 (H) **Funnel plots for changes in isokinetic (top left), isometric (top right) or isotonic (bottom center) muscle strength.**

A random-effects model was used. Abbreviations: MD, mean difference between groups; SE, standard error.


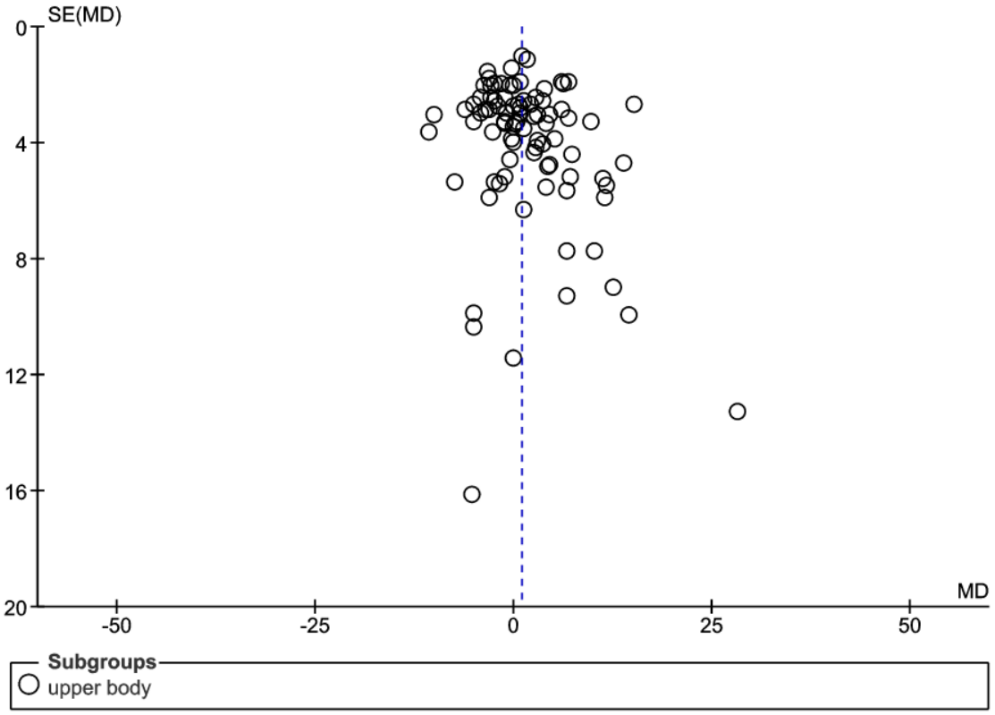

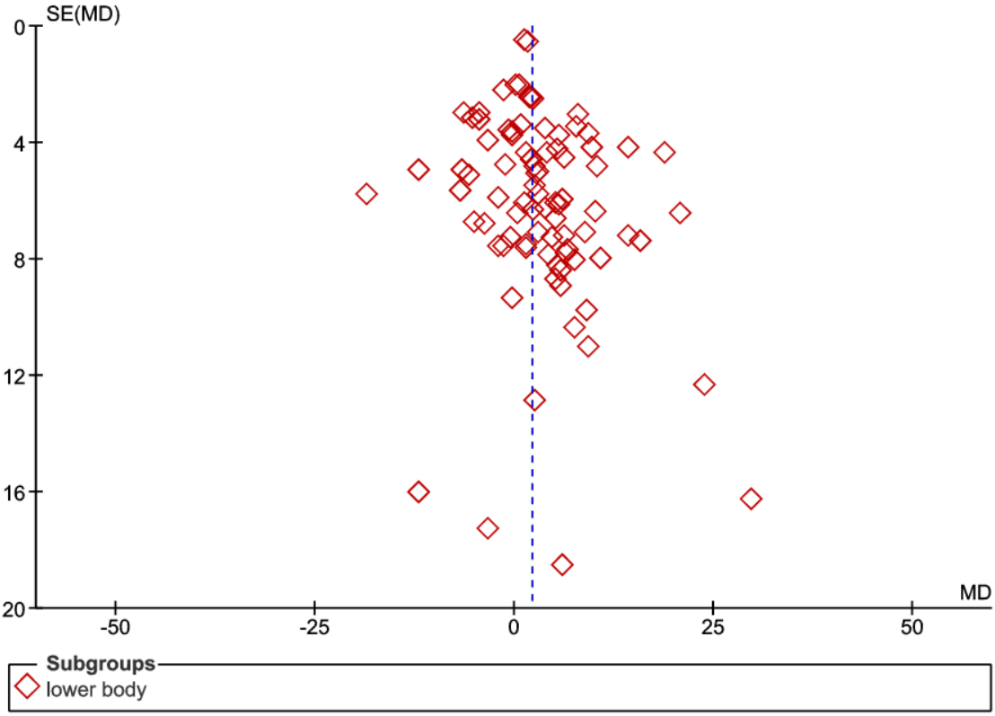


Supplementary Figure S2 (I) **Funnel plots for changes in upper-body (left) or lower-body (right) muscle strength.**

A random-effects model was used.

Abbreviations: MD, mean difference between groups; SE, standard error.


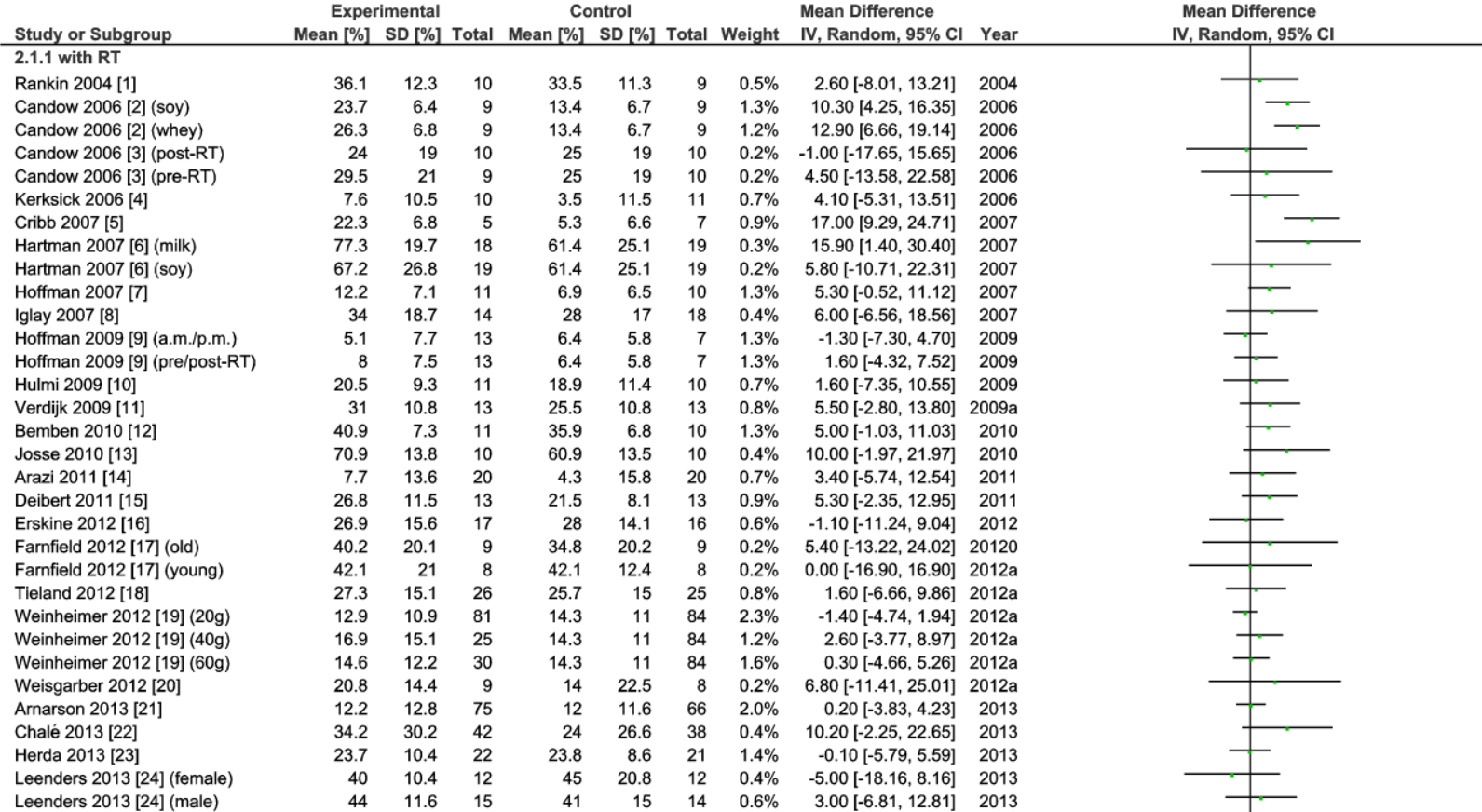


*Supplementary Figure S3* **Forest plot assessing the effect of protein supplementation on changes in muscle strength (1/5)**

A random-effects model was used. *Abbreviations*: SD, Standard Deviation; CI, confidence interval; RT, resistance training.

*
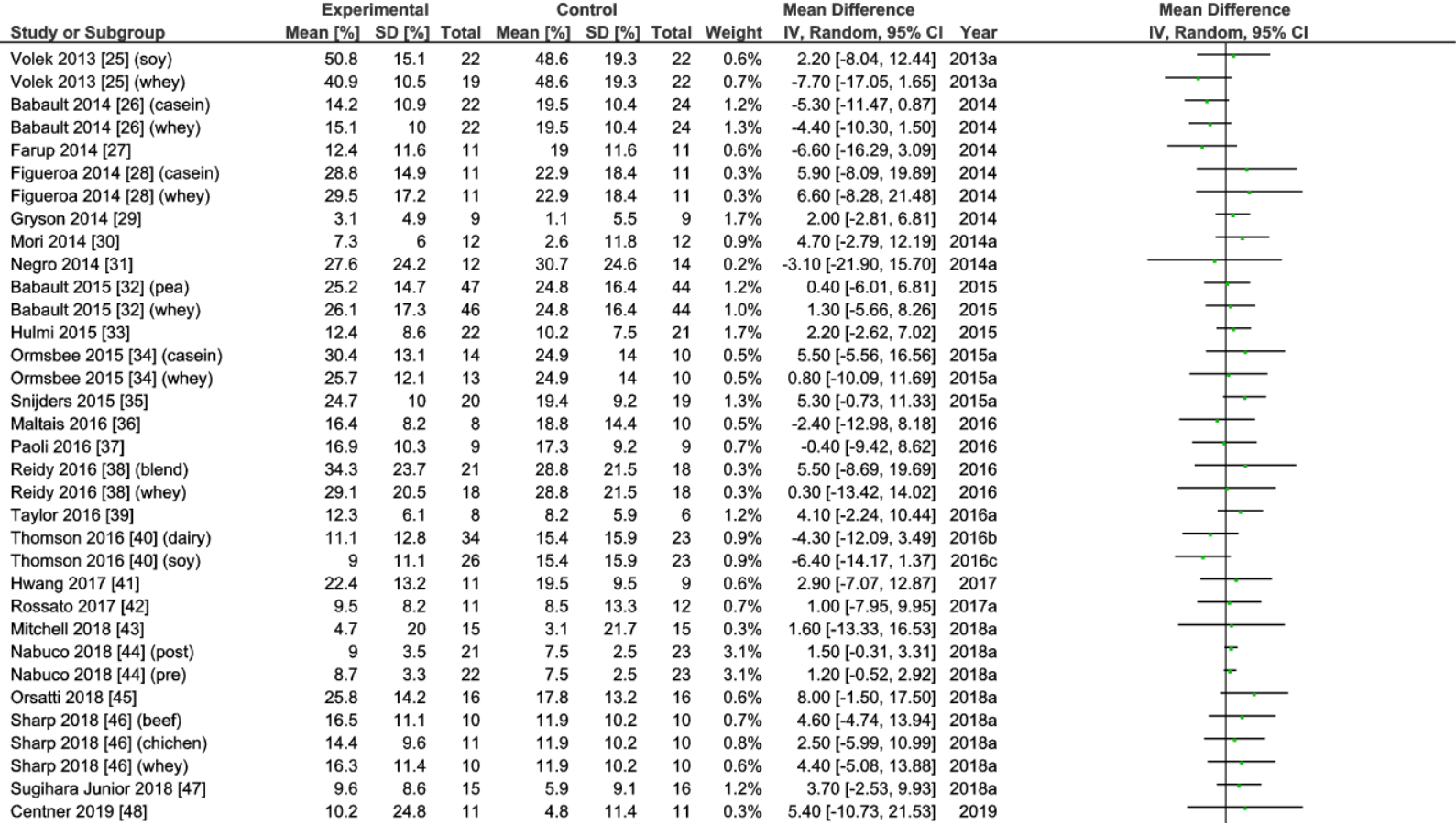
*

*Supplementary Figure S3* **Forest plot assessing the effect of protein supplementation on changes in muscle strength (2/5)**

A random-effects model was used. *Abbreviations*: SD, Standard Deviation; CI, confidence interval.


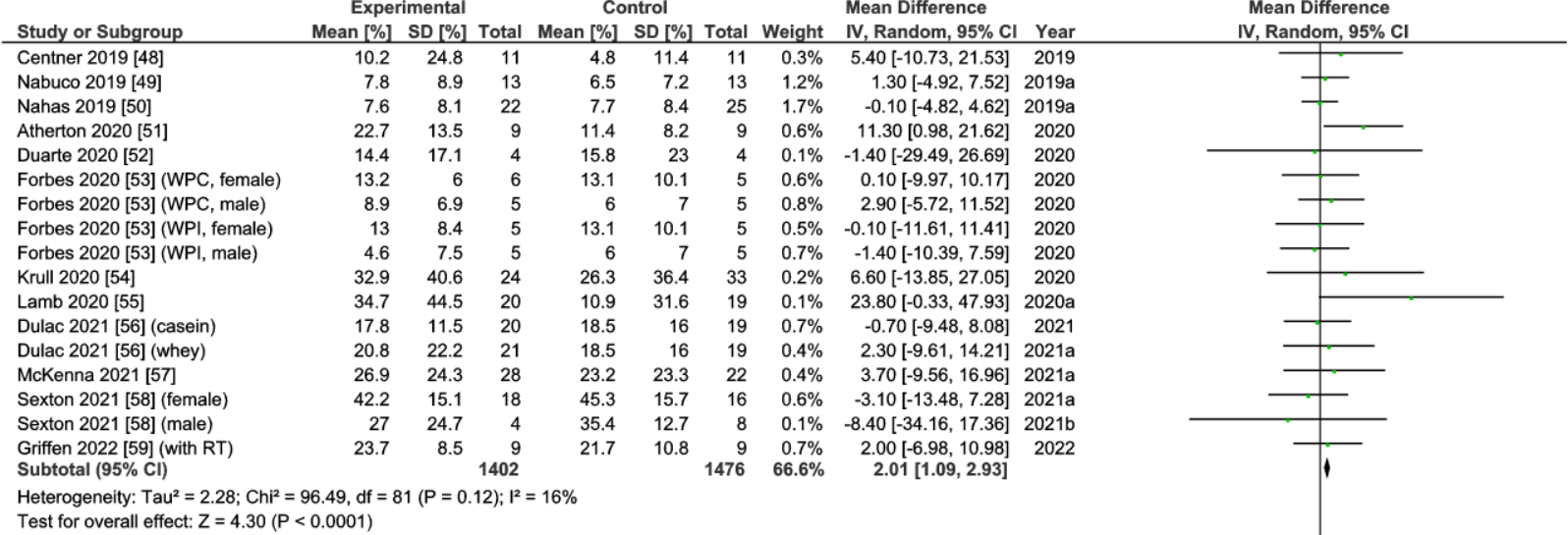


*Supplementary Figure S3* **Forest plot assessing the effect of protein supplementation on changes in muscle strength (3/5)**

A random-effects model was used. *Abbreviations*: SD, Standard Deviation; CI, confidence interval.


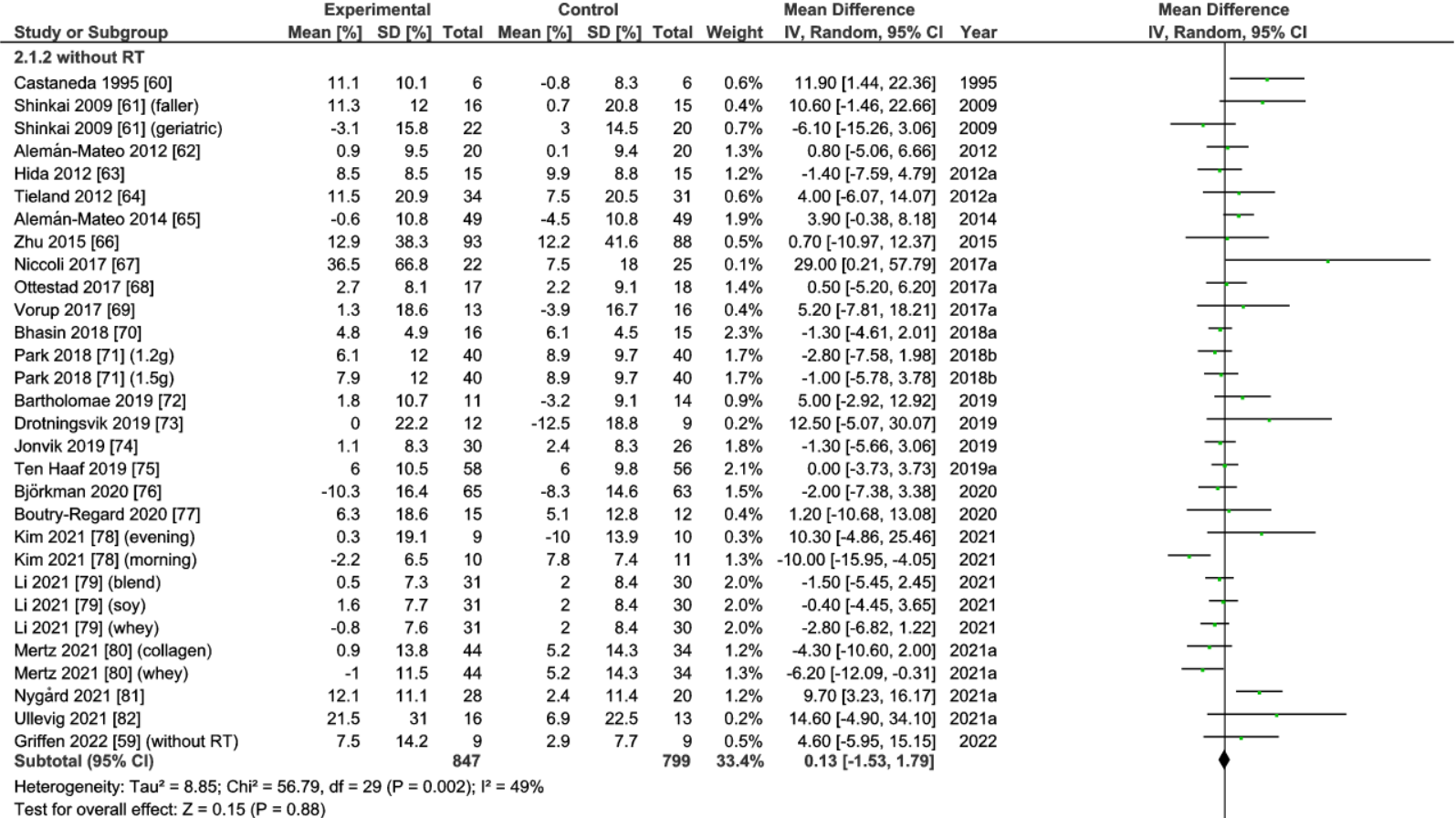


*Supplementary Figure S3* **Forest plot assessing the effect of protein supplementation on changes in muscle strength (4/5)**

A random-effects model was used. *Abbreviations*: SD, Standard Deviation; CI, confidence interval; RT, resistance training.


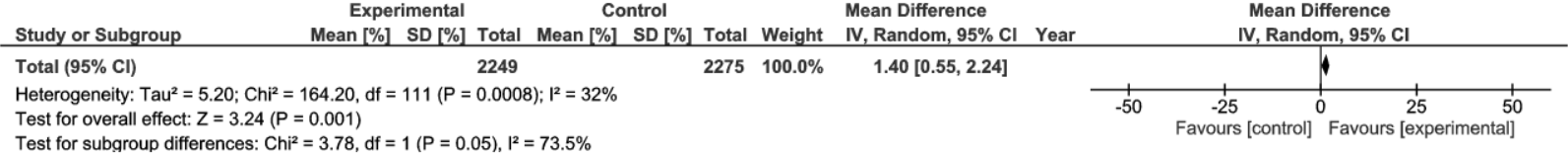


*Supplementary Figure S3* **Forest plot assessing the effect of protein supplementation on changes in muscle strength (5/5)**

A random-effects model was used. *Abbreviations*: SD, Standard Deviation; CI, confidence interval.

References

1. Rankin JW, Goldman LP, Puglisi MJ, Nickols-Richardson SM, Earthman CP, Gwazdauskas FC. Effect of post-exercise supplement consumption on adaptations to resistance training. J Am Coll Nutr. 2004 Aug;23(4):322-30.

2. Candow DG, Burke NC, Smith-Palmer T, Burke DG. Effect of whey and soy protein supplementation combined with resistance training in young adults. International journal of sport nutrition and exercise metabolism. 2006 Jun;16(3):233-44.

3. Candow DG, Chilibeck PD, Facci M, Abeysekara S, Zello GA. Protein supplementation before and after resistance training in older men. Eur J Appl Physiol. 2006 Jul;97(5):548-56.

4. Kerksick CM, Rasmussen CJ, Lancaster SL, Magu B, Smith P, Melton C, et al. The effects of protein and amino acid supplementation on performance and training adaptations during ten weeks of resistance training. J Strength Cond Res. 2006 Aug;20(3):643-53.

5. Cribb PJ, Williams AD, Stathis CG, Carey MF, Hayes A. Effects of whey isolate, creatine, and resistance training on muscle hypertrophy. Medicine and science in sports and exercise. 2007 Feb;39(2):298-307.

6. Hartman JW, Tang JE, Wilkinson SB, Tarnopolsky MA, Lawrence RL, Fullerton AV, et al. Consumption of fat-free fluid milk after resistance exercise promotes greater lean mass accretion than does consumption of soy or carbohydrate in young, novice, male weightlifters. The American journal of clinical nutrition. 2007 Aug;86(2):373-81.

7. Hoffman JR, Ratamess NA, Kang J, Falvo MJ, Faigenbaum AD. Effects of protein supplementation on muscular performance and resting hormonal changes in college football players. Journal of sports science & medicine. 2007;6(1):85-92.

8. Iglay HB, Apolzan JW, Gerrard DE, Eash JK, Anderson JC, Campbell WW. Moderately increased protein intake predominately from egg sources does not influence whole body, regional, or muscle composition responses to resistance training in older people. The journal of nutrition, health & aging. 2009 Feb;13(2):108-14.

9. Hoffman JR, Ratamess NA, Tranchina CP, Rashti SL, Kang J, Faigenbaum AD. Effect of protein-supplement timing on strength, power, and body-composition changes in resistance-trained men. International journal of sport nutrition and exercise metabolism. 2009 Apr;19(2):172-85.

10. Hulmi JJ, Tannerstedt J, Selänne H, Kainulainen H, Kovanen V, Mero AA. Resistance exercise with whey protein ingestion affects mTOR signaling pathway and myostatin in men. Journal of applied physiology (Bethesda, Md : 1985). 2009 May;106(5):1720-9.

11. Verdijk LB, Jonkers RA, Gleeson BG, Beelen M, Meijer K, Savelberg HH, et al. Protein supplementation before and after exercise does not further augment skeletal muscle hypertrophy after resistance training in elderly men. The American journal of clinical nutrition. 2009 Feb;89(2):608-16.

12. Bemben MG, Witten MS, Carter JM, Eliot KA, Knehans AW, Bemben DA. The effects of supplementation with creatine and protein on muscle strength following a traditional resistance training program in middle-aged and older men. The journal of nutrition, health & aging. 2010 Feb;14(2):155-9.

13. Josse AR, Tang JE, Tarnopolsky MA, Phillips SM. Body composition and strength changes in women with milk and resistance exercise. Medicine and science in sports and exercise. 2010 Jun;42(6):1122-30.

14. Arazi H, Hakimi M, Hoseini K. The Effects of Whey Protein Supplementation on Performance and Hormonal Adaptations Following Resistance Training in Novice Men. Baltic Journal of Health and Physical Activity. 2011;3(2):87-95.

15. Deibert P, Solleder F, Konig D, Vitolins MZ, Dickhuth HH, Gollhofer A, et al. Soy protein based supplementation supports metabolic effects of resistance training in previously untrained middle aged males. Aging Male. 2011 Dec;14(4):273-9.

16. Erskine RM, Fletcher G, Hanson B, Folland JP. Whey protein does not enhance the adaptations to elbow flexor resistance training. Medicine and science in sports and exercise. 2012 Sep;44(9):1791-800.

17. Farnfield MM, Breen L, Carey KA, Garnham A, Cameron-Smith D. Activation of mTOR signalling in young and old human skeletal muscle in response to combined resistance exercise and whey protein ingestion. Applied physiology, nutrition, and metabolism = Physiologie appliquee, nutrition et metabolisme. 2012 Feb;37(1):21-30.

18. Tieland M, Dirks ML, van der Zwaluw N, Verdijk LB, van de Rest O, de Groot LC, et al. Protein supplementation increases muscle mass gain during prolonged resistance-type exercise training in frail elderly people: a randomized, double-blind, placebo-controlled trial. Journal of the American Medical Directors Association. 2012 Oct;13(8):713-9.

19. Weinheimer EM, Conley TB, Kobza VM, Sands LP, Lim E, Janle EM, et al. Whey protein supplementation does not affect exercise training-induced changes in body composition and indices of metabolic syndrome in middle-aged overweight and obese adults. The Journal of nutrition. 2012 Aug;142(8):1532-9.

20. Weisgarber KD, Candow DG, Vogt ES. Whey protein before and during resistance exercise has no effect on muscle mass and strength in untrained young adults. International journal of sport nutrition and exercise metabolism. 2012 Dec;22(6):463-9.

21. Arnarson A, Gudny Geirsdottir O, Ramel A, Briem K, Jonsson PV, Thorsdottir I. Effects of whey proteins and carbohydrates on the efficacy of resistance training in elderly people: double blind, randomised controlled trial. Eur J Clin Nutr. 2013 Aug;67(8):821-6.

22. Chale A, Cloutier GJ, Hau C, Phillips EM, Dallal GE, Fielding RA. Efficacy of whey protein supplementation on resistance exercise-induced changes in lean mass, muscle strength, and physical function in mobility-limited older adults. J Gerontol A Biol Sci Med Sci. 2013 Jun;68(6):682-90.

23. Herda AA, Herda TJ, Costa PB, Ryan ED, Stout JR, Cramer JT. Muscle performance, size, and safety responses after eight weeks of resistance training and protein supplementation: a randomized, double-blinded, placebo-controlled clinical trial. J Strength Cond Res. 2013 Nov;27(11):3091-100.

24. Leenders M, Verdijk LB, Van der Hoeven L, Van Kranenburg J, Nilwik R, Wodzig WK, et al. Protein supplementation during resistance-type exercise training in the elderly. Medicine and science in sports and exercise. 2013 Mar;45(3):542-52.

25. Volek JS, Volk BM, Gomez AL, Kunces LJ, Kupchak BR, Freidenreich DJ, et al. Whey protein supplementation during resistance training augments lean body mass. J Am Coll Nutr. 2013;32(2):122-35.

26. Babault N, Deley G, Le Ruyet P, Morgan F, Allaert FA. Effects of soluble milk protein or casein supplementation on muscle fatigue following resistance training program: a randomized, double-blind, and placebo-controlled study. Journal of the International Society of Sports Nutrition. 2014;11:36.

27. Farup J, Rahbek SK, Vendelbo MH, Matzon A, Hindhede J, Bejder A, et al. Whey protein hydrolysate augments tendon and muscle hypertrophy independent of resistance exercise contraction mode. Scandinavian journal of medicine & science in sports. 2014 Oct;24(5):788-98.

28. Figueroa A, Wong A, Kinsey A, Kalfon R, Eddy W, Ormsbee MJ. Effects of milk proteins and combined exercise training on aortic hemodynamics and arterial stiffness in young obese women with high blood pressure. American journal of hypertension. 2014 Mar;27(3):338-44.

29. Gryson C, Ratel S, Rance M, Penando S, Bonhomme C, Le Ruyet P, et al. Four-month course of soluble milk proteins interacts with exercise to improve muscle strength and delay fatigue in elderly participants. Journal of the American Medical Directors Association. 2014 Dec;15(12):958.e1-9.

30. Mori H, Niwa M. Effect of Nutritional Care and Whey Protein Supplementation on the Body Composition and Physical Function in Older Adults after Combined Resistance and Aerobic Exercise. Jpn J Nutr Diet. 2014;72(1):12-20.

31. Negro M, Vandoni M, Ottobrini S, Codrons E, Correale L, Buonocore D, et al. Protein supplementation with low fat meat after resistance training: effects on body composition and strength. Nutrients. 2014 Aug 4;6(8):3040-9.

32. Babault N, Païzis C, Deley G, Guérin-Deremaux L, Saniez MH, Lefranc-Millot C, et al. Pea proteins oral supplementation promotes muscle thickness gains during resistance training: a double-blind, randomized, Placebo-controlled clinical trial vs. Whey protein. Journal of the International Society of Sports Nutrition. 2015;12(1):3.

33. Hulmi JJ, Laakso M, Mero AA, Hakkinen K, Ahtiainen JP, Peltonen H. The effects of whey protein with or without carbohydrates on resistance training adaptations. Journal of the International Society of Sports Nutrition. 2015;12:48.

34. Ormsbee MJ, Kinsey AW, Eddy WR, Madzima TA, Arciero PJ, Figueroa A, et al. The influence of nighttime feeding of carbohydrate or protein combined with exercise training on appetite and cardiometabolic risk in young obese women. Applied physiology, nutrition, and metabolism = Physiologie appliquee, nutrition et metabolisme. 2015 Jan;40(1):37-45.

35. Snijders T, Res PT, Smeets JS, van Vliet S, van Kranenburg J, Maase K, et al. Protein Ingestion before Sleep Increases Muscle Mass and Strength Gains during Prolonged Resistance-Type Exercise Training in Healthy Young Men. The Journal of nutrition. 2015 Jun;145(6):1178-84.

36. Maltais ML, Ladouceur JP, Dionne IJ. The Effect of Resistance Training and Different Sources of Postexercise Protein Supplementation on Muscle Mass and Physical Capacity in Sarcopenic Elderly Men. J Strength Cond Res. 2016 Jun;30(6):1680-7.

37. Paoli A, Pacelli QF, Cancellara P, Toniolo L, Moro T, Canato M, et al. Protein Supplementation Does Not Further Increase Latissimus Dorsi Muscle Fiber Hypertrophy after Eight Weeks of Resistance Training in Novice Subjects, but Partially Counteracts the Fast-to-Slow Muscle Fiber Transition. Nutrients. 2016 Jun 1;8(6).

38. Reidy PT, Borack MS, Markofski MM, Dickinson JM, Deer RR, Husaini SH, et al. Protein Supplementation Has Minimal Effects on Muscle Adaptations during Resistance Exercise Training in Young Men: A Double-Blind Randomized Clinical Trial. The Journal of nutrition. 2016 Sep;146(9):1660-9.

39. Taylor LW, Wilborn C, Roberts MD, White A, Dugan K. Eight weeks of pre- and postexercise whey protein supplementation increases lean body mass and improves performance in Division III collegiate female basketball players. Applied physiology, nutrition, and metabolism = Physiologie appliquee, nutrition et metabolisme. 2016 Mar;41(3):249-54.

40. Thomson RL, Brinkworth GD, Noakes M, Buckley JD. Muscle strength gains during resistance exercise training are attenuated with soy compared with dairy or usual protein intake in older adults: A randomized controlled trial. Clin Nutr. 2016 Feb;35(1):27-33.

41. Hwang PS, Andre TL, McKinley-Barnard SK, Morales Marroquin FE, Gann JJ, Song JJ, et al. Resistance Training-Induced Elevations in Muscular Strength in Trained Men Are Maintained After 2 Weeks of Detraining and Not Differentially Affected by Whey Protein Supplementation. J Strength Cond Res. 2017 Apr;31(4):869-81.

42. Rossato LT, Nahas PC, de Branco FMS, Martins FM, Souza AP, Carneiro MAS, et al. Higher Protein Intake Does Not Improve Lean Mass Gain When Compared with RDA Recommendation in Postmenopausal Women Following Resistance Exercise Protocol: A Randomized Clinical Trial. Nutrients. 2017 Sep 12;9(9).

43. Mitchell CJ, D'Souza RF, Mitchell SM, Figueiredo VC, Miller BF, Hamilton KL, et al. Impact of dairy protein during limb immobilization and recovery on muscle size and protein synthesis; a randomized controlled trial. Journal of applied physiology (Bethesda, Md : 1985). 2018 Mar 1;124(3):717-28.

44. Nabuco HCG, Tomeleri CM, Sugihara Junior P, Fernandes RR, Cavalcante EF, Antunes M, et al. Effects of Whey Protein Supplementation Pre- or Post-Resistance Training on Muscle Mass, Muscular Strength, and Functional Capacity in Pre-Conditioned Older Women: A Randomized Clinical Trial. Nutrients. 2018 May 3;10(5).

45. Orsatti FL, Maestá N, de Oliveira EP, Nahas Neto J, Burini RC, Nunes PRP, et al. Adding Soy Protein to Milk Enhances the Effect of Resistance Training on Muscle Strength in Postmenopausal Women. Journal of dietary supplements. 2018 Mar 4;15(2):140-52.

46. Sharp MH, Lowery RP, Shields KA, Lane JR, Gray JL, Partl JM, et al. The Effects of Beef, Chicken, or Whey Protein After Workout on Body Composition and Muscle Performance. J Strength Cond Res. 2018 Aug;32(8):2233-42.

47. Sugihara Junior P, Ribeiro AS, Nabuco HCG, Fernandes RR, Tomeleri CM, Cunha PM, et al. Effects of Whey Protein Supplementation Associated With Resistance Training on Muscular Strength, Hypertrophy, and Muscle Quality in Preconditioned Older Women. International journal of sport nutrition and exercise metabolism. 2018 Sep 1;28(5):528-35.

48. Centner C, Zdzieblik D, Roberts L, Gollhofer A, König D. Effects of Blood Flow Restriction Training with Protein Supplementation on Muscle Mass And Strength in Older Men. Journal of sports science & medicine. 2019 Sep;18(3):471-8.

49. Nabuco HCG, Tomeleri CM, Sugihara PJ, Fernandes RR, Cavalcante EF, Dos Santos L, et al. Effect of whey protein supplementation combined with resistance training on cellular health in pre-conditioned older women: A randomized, double-blind, placebo-controlled trial. Arch Gerontol Geriatr. 2019 May - Jun;82:232-7.

50. Nahas PC, Rossato LT, Martins FM, Souza AP, de Branco FMS, Carneiro MAS, et al. Moderate Increase in Protein Intake Promotes a Small Additional Improvement in Functional Capacity, But Not in Muscle Strength and Lean Mass Quality, in Postmenopausal Women Following Resistance Exercise: A Randomized Clinical Trial. Nutrients. 2019 Jun 13;11(6).

51. Atherton C, McNaughton LR, Close GL, Sparks A. Post-exercise provision of 40 g of protein during whole body resistance training further augments strength adaptations in elderly males. Research in sports medicine (Print). 2020 Oct-Dec;28(4):469-83.

52. Duarte NM, Cruz AL, Silva DC, Cruz GM. Intake of whey isolate supplement and muscle mass gains in young healthy adults when combined with resistance training: a blinded randomized clinical trial (pilot study). The Journal of sports medicine and physical fitness. 2020 Jan;60(1):75-84.

53. Forbes SC, Bell GJ. Whey protein isolate or concentrate combined with concurrent training does not augment performance, cardiorespiratory fitness, or strength adaptations. The Journal of sports medicine and physical fitness. 2020 Jun;60(6):832-40.

54. Krull MR, Howell CR, Partin RE, Lanctot J, Phipps S, Klosky JL, et al. Protein Supplementation and Resistance Training in Childhood Cancer Survivors. Medicine and science in sports and exercise. 2020 Oct;52(10):2069-77.

55. Lamb DA, Moore JH, Smith MA, Vann CG, Osburn SC, Ruple BA, et al. The effects of resistance training with or without peanut protein supplementation on skeletal muscle and strength adaptations in older individuals. Journal of the International Society of Sports Nutrition. 2020 Dec 14;17(1):66.

56. Dulac MC, Pion CH, Lemieux FC, Pinheiro Carvalho L, El Hajj Boutros G, Bélanger M, et al. Effects of slow- v. fast-digested protein supplementation combined with mixed power training on muscle function and functional capacities in older men. The British journal of nutrition. 2021 May 14;125(9):1017-33.

57. McKenna C, Salvador A, Hughes R, Scaroni S, Alamilla R, Askow A, et al. Higher protein intake during resistance training does not potentiate strength, but modulates gut microbiota, in middle-aged adults: a randomized control trial. American Journal of Physiology-Endocrinology and Metabolism. 2021 03/08;320.

58. Sexton CL, Smith MA, Smith KS, Osburn SC, Godwin JS, Ruple BA, et al. Effects of Peanut Protein Supplementation on Resistance Training Adaptations in Younger Adults. Nutrients. 2021 Nov 9;13(11).

59. Griffen C, Duncan M, Hattersley J, Weickert MO, Dallaway A, Renshaw D. Effects of resistance exercise and whey protein supplementation on skeletal muscle strength, mass, physical function, and hormonal and inflammatory biomarkers in healthy active older men: a randomised, double-blind, placebo-controlled trial. Experimental gerontology. 2022 Feb;158:111651.

60. Castaneda C, Charnley JM, Evans WJ, Crim MC. Elderly women accommodate to a low-protein diet with losses of body cell mass, muscle function, and immune response. The American journal of clinical nutrition. 1995 Jul;62(1):30-9.

61. Shinkai Shoji KH, Watanabe Naoki, Lee Sangyoon, Saito Kyoko, Suzuki Takao Randomized Controlled Trial on the Effects of Resistance Training with or without Nutritional Supplementation of Soy Peptide for the Frail Elderly. Jpn J Nutr Diet. 2009;67(2):76-83.

62. Aleman-Mateo H, Macias L, Esparza-Romero J, Astiazaran-Garcia H, Blancas AL. Physiological effects beyond the significant gain in muscle mass in sarcopenic elderly men: evidence from a randomized clinical trial using a protein-rich food. Clin Interv Aging. 2012;7:225-34.

63. Hida A, Hasegawa Y, Mekata Y, Usuda M, Masuda Y, Kawano H, et al. Effects of egg white protein supplementation on muscle strength and serum free amino acid concentrations. Nutrients. 2012 Oct 19;4(10):1504-17.

64. Tieland M, van de Rest O, Dirks ML, van der Zwaluw N, Mensink M, van Loon LJ, et al. Protein supplementation improves physical performance in frail elderly people: a randomized, double-blind, placebo-controlled trial. Journal of the American Medical Directors Association. 2012 Oct;13(8):720-6.

65. Alemán-Mateo H, Carreón VR, Macías L, Astiazaran-García H, Gallegos-Aguilar AC, Enríquez JR. Nutrient-rich dairy proteins improve appendicular skeletal muscle mass and physical performance, and attenuate the loss of muscle strength in older men and women subjects: a single-blind randomized clinical trial. Clin Interv Aging. 2014;9:1517-25.

66. Zhu K, Kerr DA, Meng X, Devine A, Solah V, Binns CW, et al. Two-Year Whey Protein Supplementation Did Not Enhance Muscle Mass and Physical Function in Well-Nourished Healthy Older Postmenopausal Women. The Journal of nutrition. 2015 Nov;145(11):2520-6.

67. Niccoli S, Kolobov A, Bon T, Rafilovich S, Munro H, Tanner K, et al. Whey Protein Supplementation Improves Rehabilitation Outcomes in Hospitalized Geriatric Patients: A Double Blinded, Randomized Controlled Trial. Journal of nutrition in gerontology and geriatrics. 2017 Oct-Dec;36(4):149-65.

68. Ottestad I, Lovstad AT, Gjevestad GO, Hamarsland H, Saltyte Benth J, Andersen LF, et al. Intake of a Protein-Enriched Milk and Effects on Muscle Mass and Strength. A 12-Week Randomized Placebo Controlled Trial among Community-Dwelling Older Adults. The journal of nutrition, health & aging. 2017;21(10):1160-9.

69. Vorup J, Pedersen MT, Brahe LK, Melcher PS, Alstrom JM, Bangsbo J. Effect of small-sided team sport training and protein intake on muscle mass, physical function and markers of health in older untrained adults: A randomized trial. PLoS One. 2017;12(10):e0186202.

70. Bhasin S, Apovian CM, Travison TG, Pencina K, Moore LL, Huang G, et al. Effect of Protein Intake on Lean Body Mass in Functionally Limited Older Men: A Randomized Clinical Trial. JAMA internal medicine. 2018 Apr 1;178(4):530-41.

71. Park Y, Choi JE, Hwang HS. Protein supplementation improves muscle mass and physical performance in undernourished prefrail and frail elderly subjects: a randomized, double-blind, placebo-controlled trial. The American journal of clinical nutrition. 2018 Nov 1;108(5):1026-33.

72. Bartholomae E, Incollingo A, Vizcaino M, Wharton C, Johnston CS. Mung Bean Protein Supplement Improves Muscular Strength in Healthy, Underactive Vegetarian Adults. Nutrients. 2019 Oct 11;11(10).

73. Drotningsvik A, Oterhals Å, Flesland O, Nygård O, Gudbrandsen OA. Fish protein supplementation in older nursing home residents: a randomised, double-blind, pilot study. Pilot and feasibility studies. 2019;5:35.

74. Jonvik KL, Paulussen KJM, Danen SL, Ceelen IJM, Horstman AM, Wardenaar FC, et al. Protein Supplementation Does Not Augment Adaptations to Endurance Exercise Training. Medicine and science in sports and exercise. 2019 Oct;51(10):2041-9.

75. Ten Haaf DSM, Eijsvogels TMH, Bongers C, Horstman AMH, Timmers S, de Groot L, et al. Protein supplementation improves lean body mass in physically active older adults: a randomized placebo-controlled trial. Journal of cachexia, sarcopenia and muscle. 2019 Apr;10(2):298-310.

76. Björkman MP, Suominen MH, Kautiainen H, Jyväkorpi SK, Finne-Soveri HU, Strandberg TE, et al. Effect of Protein Supplementation on Physical Performance in Older People With Sarcopenia-A Randomized Controlled Trial. Journal of the American Medical Directors Association. 2020 Feb;21(2):226-32.e1.

77. Boutry-Regard C, Vinyes-Parés G, Breuillé D, Moritani T. Supplementation with Whey Protein, Omega-3 Fatty Acids and Polyphenols Combined with Electrical Muscle Stimulation Increases Muscle Strength in Elderly Adults with Limited Mobility: A Randomized Controlled Trial. Nutrients. 2020 Jun 23;12(6).

78. Kim HK, Chijiki H, Fukazawa M, Okubo J, Ozaki M, Nanba T, et al. Supplementation of Protein at Breakfast Rather Than at Dinner and Lunch Is Effective on Skeletal Muscle Mass in Older Adults. Frontiers in nutrition. 2021;8:797004.

79. Li C, Meng H, Wu S, Fang A, Liao G, Tan X, et al. Daily Supplementation With Whey, Soy, or Whey-Soy Blended Protein for 6 Months Maintained Lean Muscle Mass and Physical Performance in Older Adults With Low Lean Mass. Journal of the Academy of Nutrition and Dietetics. 2021 Jun;121(6):1035-48.e6.

80. Mertz KH, Reitelseder S, Bechshoeft R, Bulow J, Højfeldt G, Jensen M, et al. The effect of daily protein supplementation, with or without resistance training for 1 year, on muscle size, strength, and function in healthy older adults: A randomized controlled trial. The American journal of clinical nutrition. 2021 Apr 6;113(4):790-800.

81. Nygård LK, Mundal I, Dahl L, Šaltytė Benth J, Rokstad AMM. Limited Benefit of Marine Protein Hydrolysate on Physical Function and Strength in Older Adults: A Randomized Controlled Trial. Marine drugs. 2021 Jan 27;19(2).

82. Ullevig SL, Zuniga K, Austin Lobitz C, Santoyo A, Yin Z. Egg protein supplementation improved upper body muscle strength and protein intake in community-dwelling older adult females who attended congregate meal sites or adult learning centers: A pilot randomized controlled trial. Nutrition and health. 2021 Nov 3:2601060211051592.
